# Relaxed Evolution in the Tyrosine Aminotransferase Gene *Tat* in Old World Fruit Bats (Chiroptera: Pteropodidae)

**DOI:** 10.1371/journal.pone.0097483

**Published:** 2014-05-13

**Authors:** Bin Shen, Tao Fang, Tianxiao Yang, Gareth Jones, David M. Irwin, Shuyi Zhang

**Affiliations:** 1 Institute of Molecular Ecology and Evolution, Institutes for Advanced Interdisciplinary Research, East China Normal University, Shanghai, China; 2 School of Biological Sciences, University of Bristol, Bristol, United Kingdom; 3 Department of Laboratory Medicine and Pathobiology, University of Toronto, Toronto, Canada; Vanderbilt University, United States of America

## Abstract

Frugivorous and nectarivorous bats fuel their metabolism mostly by using carbohydrates and allocate the restricted amounts of ingested proteins mainly for anabolic protein syntheses rather than for catabolic energy production. Thus, it is possible that genes involved in protein (amino acid) catabolism may have undergone relaxed evolution in these fruit- and nectar-eating bats. The tyrosine aminotransferase (TAT, encoded by the *Tat* gene) is the rate-limiting enzyme in the tyrosine catabolic pathway. To test whether the *Tat* gene has undergone relaxed evolution in the fruit- and nectar-eating bats, we obtained the *Tat* coding region from 20 bat species including four Old World fruit bats (Pteropodidae) and two New World fruit bats (Phyllostomidae). Phylogenetic reconstructions revealed a gene tree in which all echolocating bats (including the New World fruit bats) formed a monophyletic group. The phylogenetic conflict appears to stem from accelerated TAT protein sequence evolution in the Old World fruit bats. Our molecular evolutionary analyses confirmed a change in the selection pressure acting on *Tat*, which was likely caused by a relaxation of the evolutionary constraints on the *Tat* gene in the Old World fruit bats. Hepatic TAT activity assays showed that TAT activities in species of the Old World fruit bats are significantly lower than those of insectivorous bats and omnivorous mice, which was not caused by a change in TAT protein levels in the liver. Our study provides unambiguous evidence that the *Tat* gene has undergone relaxed evolution in the Old World fruit bats in response to changes in their metabolism due to the evolution of their special diet.

## Introduction

In mammals, the liver is the major organ involved with amino acid metabolism. In addition to protein synthesis, tyrosine (Tyr) is catabolized to acetoacetate and fumarate for energy production by the tyrosine catabolic pathway, which includes four steps that are catalyzed in turn by tyrosine aminotransferase (TAT), 4-hydroxyphenylpyruvic acid dioxygenase (HPD), homogentisate 1,2-dioxygenase (HGD) and fumarylacetoacetase (FAH) [1]. TAT (encoded by the *Tat* gene) is the rate-limiting enzyme in this pathway [2] and catalyzes the conversion of tyrosine to *p*-hydroxyphenylpyruvate. The *Tat* gene consists of 11 exons [3], [4] and its expression is regulated by glucocorticoids, glucagon and insulin [5], [6]. Since the accumulation of tyrosine in blood causes toxic effects to tissues and organs [7], the breakdown of tyrosine by TAT is very important for human health. Deficiency of TAT, which is caused by genetic mutations in the *Tat* gene, in humans leads to tyrosinemia type II syndrome characterized by elevated blood tyrosine levels, mental retardation, etc [8]. To date, more than 15 distinct mutations [8], [9], [10], [11], [12], [13], [14], [15] have been identified in humans that cause tyrosinemia type II.

Bats (Order: Chiroptera) are the second largest mammalian order (after rodents) with ∼1200 species identified to date [16]. In mammals, extant bats exhibit perhaps the most diverse food habits including insectivory, carnivory, piscivory, sanguivory, frugivory and nectarivory [17], [18], which are considered to have evolved (in some cases several times independently) from an insectivorous bat ancestor [19]. Two lineages of bats have independently evolved a diet comprising mainly of fruit and/or nectar, the Old World fruit bats from the family Pteropodidae belonging to the suborder Yinpterochiroptera, and the New World fruit bats from the family Phyllostomidae belonging to the suborder Yangochiroptera [18], [20], [21]. The diets of bats in these two lineages is rich in carbohydrate but poor in nitrogen, as fruits contain only 0.2–1.4% (dry weight) protein [17] and nectar also consists of only a trace amount of amino acids [22]. To adapt to such diets, behavioral [23], physiological [24], [25], [26], [27], [28], biochemical [29] and recently molecular evolutionary [30], [31], [32] adaptations have been revealed in these fruit- and nectar-eating bats in relation to blood glucose regulation and carbohydrate metabolism.

Both Old World and New World fruit bats fuel their metabolism mostly by using carbohydrates [33], [34], [35], [36]. On the other hand, due to the restricted protein content of their diet, these fruit- and nectar-eating bats likely allocate the ingested proteins mainly for the synthesis of new proteins rather than for energy production via catabolism [34], [35]. Thus, it is possible that both Old World and New World fruit bats have reduced their reliance on protein catabolism for energy supply. Recently, Pan et al. (2013) found that the expression levels of enzymes involved in the phenylalanine (phenylalanine hydroxylase, PAH) and tyrosine (HPD, HGD and FAH) catabolic pathway in the Old World fruit bat *Cynopterus sphinx* are significantly lower than those in the insectivorous bats *Rhinolophus ferrumequinum* and *Myotis ricketti* [37]. Moreover, relaxation of the evolutionary constraints on the genes encoding PAH, HPD and HGD were detected in Old World fruit bats, including some amino acid substitutions that should cause adverse effects to their enzymatic activity [37]. All these results strongly indicated that the Old World fruit bats have reduced their reliance on the phenylalanine and tyrosine catabolic pathway in relation to their special diet and associated metabolism, leading us to speculate that the *Tat* gene, which is involved in the rate-limiting step of tyrosine catabolism, may have undergone relaxed evolution in fruit- and nectar-eating bats.

To test our hypothesis, we obtained the coding sequences of the *Tat* gene from a taxonomically wide range of bats (including four Old World fruit bats and two New World fruit bats), and examined the molecular evolution of this gene in bats and other mammals. To test results predicted from these molecular evolutionary analyses, we conducted biochemical and Western blot assays to determine the enzymatic activity and expression levels of TAT in representative species of frugivorous and insectivorous bats.

## Materials and Methods

### Ethics Statement

Protocols including bat species collection and tissue sampling in China were approved by the Animal Ethics Committee of East China Normal University (ID No: AR2012/03001). The Neotropical bat species were sampled in Mexico during April, 2010 for our previous study [38], with assistance from Professor Rodrigo A. Medellín and Dr. Rafael Avila-Flores of the Instituto de Ecología, Universidad Nacional Autónoma de México, under the scientific collecting license (No. FAUT-0250) provided by the Secretaría de Medio Ambiente y Recursos Naturales (México). The purchase, sacrifice and tissue sampling of mice were conducted under an approved protocol of the Animal Ethics Committee of East China Normal University (ID No: 20090219 and 20101002). No bat species used for this study are considered endangered. Bats were sacrificed by quick decapitation. No *in vivo* experiments involved bats or mice in our study.

### Taxonomic Coverage

We studied the evolution of the *Tat* gene in 20 bat species covering nine of the 17 extant chiropteran families. From the suborder Yinpterochiroptera, we included four Old World fruit bats from the family Pteropodidae (*Cynopterus sphinx*, *Rousettus leschenaultii*, *Eonycteris spelaea* and *Pteropus vampyrus*). Also from this suborder, we included six insectivorous bats from sister families to the Old World fruit bats: *Rhinolophus ferrumequinum* and *R. pusillus* (Rhinolophidae), *Hipposideros armiger* and *H. pratti* (Hipposideridae) and *Megaderma spasma* and *M. lyra* (Megadermatidae). From the suborder Yangochiroptera, we included two New World fruit bats *Artibeus lituratus* and *Leptonycteris yerbabuenae* (Phyllostomidae) and seven insectivorous bats from four families: *Mormoops megalophylla* and *Pteronotus parnellii* (Mormoopidae), *Myotis ricketti*, *Scotophilus kuhlii* and *Pipistrellus abramus* (Vespertilionidae), *Miniopterus fuliginosus* (Miniopteridae), *Tadarida plicata* (Molossidae). We also sequenced the *Tat* gene of one sanguivorous species *Desmodus rotundus* (Phyllostomidae), a close relative of the New World fruit bats. All new *Tat* sequences were deposited to GenBank and accession numbers are KJ161833–KJ161851.

We also incorporated available published sequences for our analyses. We obtained the *Tat* coding sequence of the frugivorous bat *P. vampyrus* from the Ensembl database and coding sequences of eight other mammal species from GenBank: *Homo sapiens* (NM_000353), *Mus musculus* (NM_146214), *Rattus norvegicus* (NM_012668), *Bos taurus* (XM_005218654), *Canis lupus familiaris* (XM_536796), *Ailuropoda melanoleuca* (XM_002920948), *Sus scrofa* (XM_003126884) and *Equus caballus* (XM_001498000). Details of all bat species and their corresponding accession numbers are listed in Table S1.

### Isolation, Amplification and Sequencing

*Tat* coding region sequences were determined for 19 bat species. Total RNA was isolated from liver tissue (stored at −80°C) of euthanized bats using Trizol reagent (Invitrogen). Following the standard protocol, 5 ug total RNA was reverse-transcribed into cDNA by SuperScript III Reverse Transcriptase kit (Invitrogen). Two pairs of primers were designed based on sequences spanning untranslated regions and exons (Figure S1A) to amplify the coding sequences of the *Tat* gene (See Table S2 for information on the primers and the corresponding bat species). Polymerase Chain Reactions (PCR) were conducted using Premix Ex Taq (TaKaRa) with the following conditions: denaturation at 95°C for 5 min, 32 amplification cycles (95°C for 30 s, 60°C for 30 s, 72°C for 1.5 min), and a final extension at 72°C for 10 min. All PCR products were isolated using 1% agarose gels and purified with Gel Extraction Kits (Qiagen), ligated into pGEM-T easy vector (Promega), cloned and sequenced using the Terminator kits (Applied Biosystems) on an ABI 3730 DNA sequencer.

### Phylogenetic Reconstruction

The nucleotide sequences of 28 species were aligned using ClustalX [39] and checked for accuracy by eye, and coding sequences were translated to amino acids using MEGA4 [40]. The first 23 amino acids that show limited sequence conservation at the amino-termini of TAT (Figure S2) were removed from the dataset prior to phylogenetic reconstruction and subsequent molecular evolutionary analyses.

Before phylogenetic reconstruction and molecular evolutionary analyses, we first examined whether there was evidence of recombination in our dataset, as recombination is known to cause adverse effects to the power and accuracy of phylogenetic reconstruction [41] and molecular evolutionary analyses (e.g., the detection of positive selection) [42]. We utilized GARD [43] in the HyPhy package [44] to detect evidence for statistically supported recombination breakpoints (thus recombination) in our dataset.

A Bayesian phylogenetic tree was reconstructed based on the aligned nucleotide sequences using MrBayes 3.1.2 [45] with the K80+ Gamma (Γ) nucleotide substitution model that was selected as the best fit by jModelTest0.1 [46]. For the Bayesian analysis, 10,000,000 generations of MCMC were performed with sampling frequency set as every 100th generation. The first 2,000,000 generations were discarded as burn-in, since the standard deviations of the split frequencies were stable below 0.01 after 2,000,000 generations of MCMC performance. All other options and priors were the default settings of the MrBayes 3.1.2. Maximum-likelihood analysis was also conducted using RaxML v7.0.4 [47] under the General Time Reversible (GTR)+Γ nucleotide substitution model with four discrete rate categories using the rapid hill-climbing algorithm. Two hundred replicates of RaxML searches were performed with a complete random starting tree and nodal supports were determined by non-parametric bootstrapping with 1,000 RaxML bootstrap replicates. Finally, a neighbor-joining (NJ) tree was reconstructed from MEGA4 using Kimura 2-parameter algorithm with 2,000 bootstrap replicates. Besides, synonymous and nonsynonymous substitution trees of *Tat* gene were also conducted in MEGA4, using Kumar Model [48] with 2,000 bootstrap replicates.

To assess the relative support for reconstructed gene tree versus the accepted species topology, when phylogenetic conflicts occurred between gene tree and accepted species tree, we also reconstructed a constrained nucleotide tree using maximum-likelihood analysis in RaxML v7.0.4. Site-wise log-likelihood values were calculated using RaxML v7.0.4 for the reconstructed gene tree and species topology and used to conduct an approximately unbiased test [49] in CONSEL [50]. The nodes forced in the species topology were (*C. sphinx*, *R. leschenaultii*, *E. spelaea*, *P. vampyrus*), (*R. ferrumequinum*, *R. pusillus*, *H. armiger*, *H. pratti*, *M. spasma*, *M. lyra*) and (*M. megalophylla*, *P. parnellii*, *D. rotundus*, *A. lituratus*, *L. yerbabuenae*, *M. ricketti*, *S. kuhlii*, *P. abramus*, *M. fuliginosus*, *T. plicata*).

### Molecular Evolutionary Analyses

For the selection tests on the *Tat* gene, we estimated the rate of nonsynonymous substitutions (d_N_) and synonymous substitutions (d_S_) using PAML CODEML [51] with a species topology of 28 mammals based on the accepted species relationships inferred from multi-gene dataset [21], [52], [53], [54]. We conducted a branch model test using the two-ratio model, where the d_N_/d_S_ ratio (termed as omega or ω) was allowed to vary between the fixed branch (foreground) and other branches (background). Firstly, to test the selective pressure acting on the *Tat* gene in fruit- and nectar-eating bats, separate two-ratio models were tested with the foreground branch set as the ancestral branch leading to the Old World fruit bats and the New World fruit bats. In addition, to compare the evolutionary patterns of the *Tat* gene between frugivorous and insectivorous bats, we also conducted two-ratio model tests for the ancestral branches leading to Chiroptera (all bats), Yinpterochiroptera, yinpterochiropteran echolocating bats and Yangochiroptera. In all cases, the one-ratio model in which an equal d_N_/d_S_ ratio was assumed among all branches was used as a null model.

To further confirm the results from the two-ratio model tests, we used “TestBranchDNDS.bf” in the HyPhy package to examine whether *Tat* sequences in the ancestral branches leading to the Old World fruit bats, the New World fruit bats and the four insectivorous bats analysed with two-ratio models evolved under different selection pressures compared with other branches. The analyses were conducted under a nucleotide substitution model named “012232” selected by the Datamonkey web server (http://www.datamonkey.org/), using the complete site-to-site rate variation model, four rate classes and a polarity/charge/hydrophobicity-based amino acid model.

Finally, the pairwise relative rate test in the HyPhy package was used to test for significant differences of the synonymous and nonsynonymous rates of the *Tat* gene between fruit- and nectar-eating bats and insectivorous bats. For analysis, mammalian species of human, mouse, rat, cow, pig, horse, dog and panda were used as outgroup respectively to test significant differences of the synonymous and nonsynonymous rates of *Tat* gene between all possible pairs of bat species. The Muse-Gaut 94 codon model [55] was used for analyses.

### TAT Activity Assays

Hepatic TAT activity was determined and compared between frugivorous and insectivorous bats. Six representative bat species were used: *C. sphinx* and *R. leschenaultii* (frugivorous bats from the Old World fruit bats, n = 3 and 4, respectively), *R. ferrumequinum* and *H. armiger* (yinpterochiropteran insectivorous bats, n = 5 and 3, respectively) and *M. ricketti* and *T. plicata* (yangochiropteran insectivorous bats, n = 4 and 3, respectively). Mouse (n = 3) was used as an omnivorous mammal outgroup. Adult individuals of these six bat species were sampled from the wild in China. Bat individuals were sacrificed humanely after capture and the liver and other major tissues were collected and immediately stored in liquid nitrogen. C57BL/6 mice (8 weeks old) were purchased from Shanghai Slac Laboratory Animal Co. Ltd. (Shanghai, China). Mice were sacrificed humanely by quick cervical dislocation, with the liver and other major tissues collected and stored in liquid nitrogen.

Liver tissue (∼100 mg) was homogenized using a Precellys 24 grinder (Bertin technologies, France) with 4 volumes of ice-cold 0.14 mol/L KCl. The homogenate was centrifuged at 20,000 g for 30 min at 0°C using Heraeus Multifuge X1R centrifuge (Thermo Electron LED GmbH, Langenselbold, Germany), and the supernatant was used as a crude enzyme solution. The crude enzyme solution was stored in 0∼4°C for at least 36 h before enzyme assays to inactivate endogenous enzymes that catalyze the degeneration of *p*-hydroxyphenylpyruvate [56]. The protein concentration of each enzyme extract was assessed using one aliquot of each crude enzyme solution using the Quick Start Bradford protein assay kit (Bio-Rad, USA) according to the manufacturer’s protocol.

TAT activity was measured using the methods described by Diamondstone (1966) [56]. This fixed-time assay was based on the strong alkali-catalyzed conversion of the reaction product *p*-hydroxyphenylpyruvate to *p*-hydroxybenzaldehyde. The standard reaction mixture contained 2.8 ml of 0.2 mol/L potassium phosphate buffer (pH 7.3) with 19.2 umol L-tyrosine (Sigma), 0.1 ml of a buffer solution of 0.3 mol/L α-ketoglutarate (Sigma), 0.1 ml of a buffer solution of 1.3 mmol/L pyridoxal phosphate (Sigma) and 0.2 ml of 8- to 10-fold diluted crude enzyme solution, in a total volume of 3.2 ml.

The crude enzyme solution was first preincubated with L-tyrosine and pyridoxal phosphate in a water bath at 37°C for 1 h. The reaction was started by the addition of α-ketoglutarate and was allowed to proceed for 10 min at 37°C in a water bath. The reaction was then stopped by the addition of 0.2 ml of 10 mol/L NaOH. Optical density of the solution was determined at 331 nm against a null-time control (adding NaOH before the α-ketoglutarate) using a Unico UV-2800H ultraviolet spectrophotometer after 30 min at room temperature. An extinction coefficient of 19,900/M per cm was used for the molar absorbance of *p*-hydroxybenzaldehyde. Enzyme activity was expressed as nmol of the reaction product (*p*-hydroxybenzaldehyde) formed/mg protein per min (mean ± SD). Statistical significance of the TAT activity among species was determined by one-way ANOVA, followed by Fisher’s least significant difference (LSD) *post hoc* test. A *P*-value<0.05 was considered as significant.

### Western Blot Validation

Western blot assays were conducted to examine the expression levels of TAT among the same individuals of species that were used for the enzyme assays (*C. sphinx*, *R. leschenaultii*, *R. ferrumequinum*, *H. armiger*, *M. ricketti*, *T. plicata* and mouse, n = 3 for each species). Liver tissues (100 mg) of the six species of bats and mouse were homogenized using a Precellys 24 grinder (Bertin technologies, France) with 1 ml of lysis buffer [2.2% sodiumdodecyl sulfate (SDS), 62.5 mmol/L Tris-HCl, pH 6.8]. Homogenates were boiled for 10 min at 100°C using AccuBlock Digital Dry Bath (Labnet international, Inc.) and cooled on ice before a quick spin. Each homogenate was diluted 30- to 40-fold with ddH_2_O, and the protein content of each sample was determined using the Quick Start Bradford protein assay kit (Bio-Rad, USA) following the standard protocol. For each species, 20 ug of protein was loaded and separated by 10% SDS-polyacrylamide gel electrophoresis (PAGE) using an SE 260 electrophoresis unit (GE Healthcare). After electrophoresis, the proteins in the SDS-PAGE were transferred onto a polyvinylidene fluoride (PVDF) membrane (Millipore). Reversible Ponceau staining of the membrane was conducted before blocking to examine for equal gel loading and transfer efficiency [57]. The protein-bearing PVDF membranes were then blocked with blocking solution (5% skimmed milk and 1% bovine serum albumin) for 1 h and then reacted with 1∶200 diluted anti-TAT monoclonal antibody (sc-376292, Santa Cruz Biotechnology, Inc.) in TBS-T. After washing, the blots were treated with 1∶5000 diluted secondary antibody (anti-mouse IgG, Santa Cruz Biotechnology, Inc.) following the manufacturer’s protocol. Images of the PVDF membrane were acquired with the ImageQuant LAS-4000 (Amersham Biosciences), and band intensities were quantified with the ImageQuant TL software (version 7.0, Amersham Biosciences). The intensity of each band was normalized using the corresponding Ponceau-stained protein band and the results were expressed as means ± SD. Statistical significance of the TAT expression level among species was determined by one-way ANOVA, followed by Fisher’s least significant difference (LSD) *post hoc* test. *P*-value<0.05 were considered significant.

## Results

In this study, we sequenced the *Tat* coding region and compared the evolution of the sequences between frugivorous and insectivorous bats (for convenience, the sanguivorous *D. rotundus* grouped with insectivorous bats as it feeds on animals). Our final *Tat* gene sequence dataset comprised 28 taxa, including four Old World fruit bats, two New World fruit bats and 14 insectivorous bat species. The alignment of the *Tat* coding sequence spanned 1362 nucleotides, encoding 454 amino acids (Figure S2), of which 182 amino acids (∼43.8%) were variable in bats and other mammalian groups (Figure S3). Our sequence alignment showed that the amino-terminal 40 aa of TAT were not conserved among bats and other mammal species (Figure S2). Besides, the amino-termini of TAT sequence in four Old World fruit bats are quite different from that of most insectivorous bats and other mammals, resulting a shorter TAT sequence (447 aa) as compare with other species (such as 454 aa in human TAT) (Figure S2). After alignment and comparison of *Tat* gene sequences of the four Old World fruit bats with that of human, we found that the shorter TAT sequence in Old World fruit bats was caused by a genomic deletion of seven amino acids from positions 3 to 9 in the first exon of the gene (Figure S1B). In addition the deletion in the amino-terminus of the TAT sequences in the four Old World fruit bats, these sequences also contained 21 shared amino acid changes (A39P, K51R, F53Y, V65M, K66E, V83L, V93I, T94I, M97L, V164A, L171P, K232D, S258T, L263I, S268R, D307N, L317M, G355E, R395Q, V397F and A398V, amino acid position refers to the human TAT sequence) (Figure S2 and Figure S4), indicating a large variation in the TAT sequence of species of this bat lineage. No similar amino acid changes were observed in the two New World fruit bat species (Figure S2 and Figure S4).

No evidence for recombination breakpoints (thus recombination) were detected in our *Tat* dataset, thus the potential adverse influence of recombination to phylogenetic reconstruction and subsequent molecular evolutionary analyses could be excluded.

**Figure 1:**
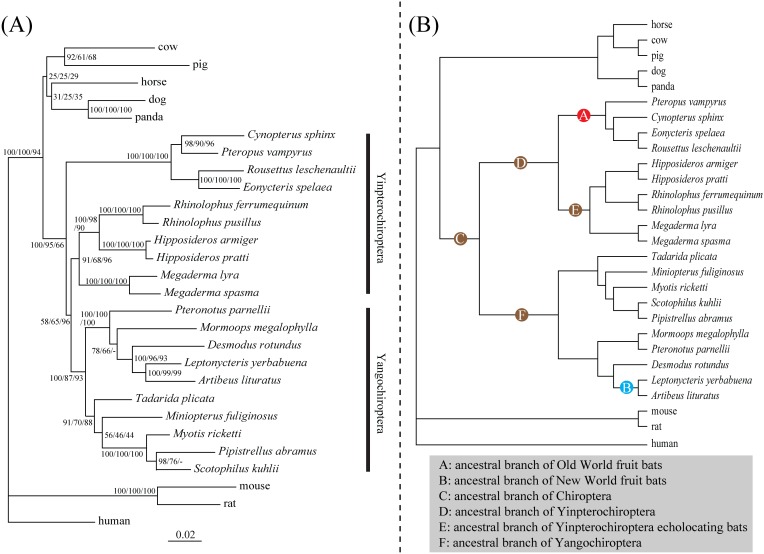
Unconstrained Bayesian phylogenetic tree and species topology. (A) Unconstrained Bayesian phylogenetic tree based on *Tat* gene coding sequences. Values on the nodes indicate statistical supports from Bayesian, maximum-likelihood and neighbor-joining, respectively. (B) The species tree of 28 mammals based on accepted species relationships (see Materials and Methods for references). Six branches tested by the two-ratio model tests and the TestBranchDNDS tests are marked by A, B, C, D, E and F, respectively.

Our phylogenetic reconstruction analyses based on the *Tat* nucleotide sequences from 28 species revealed trees where the main groupings agreed with the accepted mammalian species relationships based on multi-gene phylogenies (Figure 1B) [21], [52], [53], [54]. Rodents (mouse and rat) and human grouped together in the superorder Euarchontoglires, and members of carnivores, artiodactyls, perrisodactyls and bats grouped together in the superorder Laurasiatheria (Figure 1A). Moreover, the monophyly of Chiroptera was supported by our Bayesian and maximum likelihood (ML) approaches with high nodal supports [Bayesian posterior probability (BPP) of 100% and ML bootstrap 95%] and by neighbor-joining (NJ) method with moderate bootstrap value (NJ bootstrap 63%) (Figure 1A). However, a conflicting phylogenetic signal was found within clade of Chiroptera, where all of our phylogenetic reconstruction analyses recovered a tree (termed as ‘gene tree’) in which the yinpterochiropteran insectivorous bats (*R. ferrumequinum* and *R. pusillus* from Rhinolophidae, *Hipposideros armiger* and *H. pratti* from Hipposideridae and *Megaderma spasma* and *M. lyra* from Megadermatidae) are grouped with echolocating yangochiropteran insectivorous bats (including the New World fruit bats) instead of their close relatives from the family Pteropodidae (58% BPP, 65% ML bootstrap and 98% NJ bootstrap) (Figure 1A). To check whether the phylogenetic conflict between the accepted species tree and the gene tree is caused by the amino acid substitutions found in the four Old World fruit bats, we repeated phylogenetic reconstructions based on synonymous and nonsynonymous substitutions respectively using nucleotide dataset removing codons for 21 shared amino acid changes in the four Old World fruit bats. Our phylogenetic reconstruction analyses based on synonymous substitutions revealed an accepted species tree that the Old World fruit bats are grouped with the yinpterochiropteran insectivorous bats to form the suborder Yinpterochiroptera (Figure S5A). However, the phylogenetic reconstruction analyses based on nonsynonymous substitutions still revealed a tree in which the yinpterochiropteran insectivorous bats are grouped with echolocating yangochiropteran insectivorous bats (including the New World fruit bats), while the Old World fruit bats are grouped with other outgroup mammals (Figure S5B). These results indicated that the phylogenetic conflict between the accepted species tree and the reconstructed gene tree might be caused by accelerated evolutionary rate of the *Tat* gene in the Old World fruit bats.

**Figure 2:**
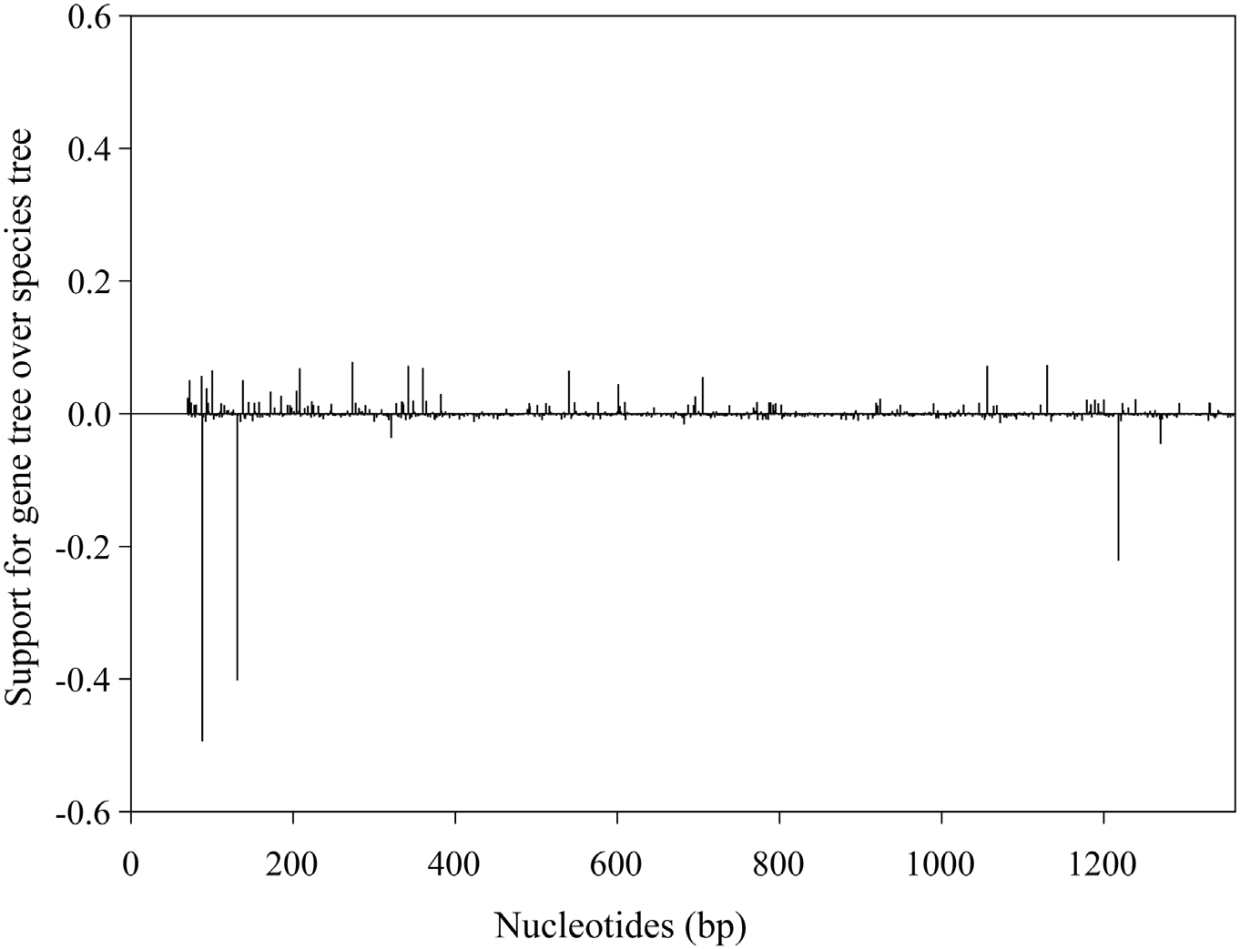
Relative support for gene tree over species tree along the *Tat* gene open reading frame. Values are the difference between site-wise negative log-likelihood scores for the species tree minus the site-wise negative log-likelihood scores for the gene tree. Positive values indicate greater support for gene tree, and negative values indicate greater support for species tree.

To access the relative support for the gene tree versus the species tree, we conducted an approximately unbiased test. Our results indicated greater support for the reconstructed gene tree over the species tree, although this difference was not significant (*P* = 0.650 and 0.350 for gene tree and species tree, respectively). To further test the difference between the gene tree and the species tree, we conducted a parametric bootstrapping analyses with simulated sequences based on a null hypothesis (constrained species tree) (see Methods S1). Similar to the results of the approximately unbiased test, the results of the parametric bootstrapping analyses showed that the recovered gene tree could not reject the null hypothesis (species tree) (*P*-value = 0.295, Figure S6). Spectral analyses of site-wise log-likelihood values revealed a mixed phylogenetic signal that support both the gene tree and species tree, with the phylogenetic signals supporting the gene tree distributed across the entire *Tat* open reading frame (Figure 2) and not due to localized region yielding an unexpected result. This result may explain why the nonsynonymous tree based on nucleotide dataset removing codons for 21 shared amino acid changes in the four Old World fruit bats is still inconsistent with the accepted species tree.

In order to detect changes in the selection pressure acting on the *Tat* gene in frugivorous and insectivorous bats, a number of two-ratio model tests were conducted on the six branches marked A∼F in Figure 1B. Detailed results of the two-ratio model tests are shown in Table 1. Our results showed that a two-ratio model which designed the ancestral branch leading to the Old World fruit bats (branch marked as A in Figure 1B) as foreground was a significantly better fit to the dataset than the null (one-ratio) model [likelihood ratio test (LRT) statistic (2Δ*ℓ*) = 25.398, df = 1, *P*<0.001] (Table 1). This two-ratio model estimates a larger value for ω on the ancestral branch for Old World fruit bats than for all other branches (0.585 versus 0.153, respectively), indicating a change in the selection pressure acting on the *Tat* gene on this bat lineage. In accordance with these results, our TestBranchDNDS tests also showed that the d_N_/d_S_ values are significantly different in the Old World fruit bats compared with other branches (*P* = 0.021). These results thus concur with the results of TAT sequence alignment that showed an extensive amino acid changes in the sequences from species of Old World fruit bats (Figure S2). However, no such change in selection pressure was detected on the ancestral branch for the New World fruit bats, as the two-ratio model test that set the ancestral branch of New World fruit bats (branch marked as B in Figure 1B) as the foreground showed no significant better fit than the one ratio model (2Δ*ℓ* = 6.321, df = 1, *P*>0.05) (Table 1). Similarly, our results with the TestBranchDNDS tests showed that the d_N_/d_S_ values in the New World fruit bats are not significantly different from that of other branches (*P* = 0.094). For the insectivorous bats, our results from the two-ratio model tests, which set the ancestral branches leading to Chiroptera (all bats), Yinpterochiroptera, yinpterochiropteran echolocating bats and Yangochiroptera (branches marked with C, D, E and F in Figure 1B respectively), indicated that no change occurred in selection pressure on the *Tat* gene in those major focal branches of insectivorous bats (Table 1). These results were confirmed by our TestBranchDNDS tests where the d_N_/d_S_ values of these branches are not significantly different from those of other branches (*P* = 0.727, 0.996, 0.578 and 0.986, respectively). Our results of pairwise relative rate tests showed that nonsynonymous rates but not synonymous rates of the *Tat* gene are significantly different between Old World fruit bats and insectivorous bats (Table S3). Taken together, these results indicated that the *Tat* gene has undergone purifying selection in insectivorous bats and the New World fruit bats but has undergone a strong change in selection pressure in the Old World fruit bats, which is caused by significant nonsynonymous rate change of the gene in bats of this lineage.

**Table 1:** Results of two-ratio model tests of selection pressure on the *Tat* gene in bats.

Model^a^	np^b^	*ℓ*	ω_0_ c	ω_Fix_ c	ModelCompared	2Δ*ℓ*	*P*-value^d^
0. One ratio: ω_0_	55	−8406.72	0.165	= ω_0_			
1. Two ratios: ω_0_, ω_A_	56	−8394.02	0.153	**0.585**	1 vs. 0	25.398	**<0.001**
2. Two ratios: ω_0,_ ω_B_	56	−8403.56	0.166	0.0001	2 vs. 0	6.321	>0.05
3. Two ratios: ω_0,_ ω_C_	56	−8406.72	0.165	0.169	3 vs. 0	0.001	>0.05
4. Two ratios: ω_0,_ ω_D_	56	−8406.72	0.165	0.0001	4 vs. 0	0.001	>0.05
5. Two ratios: ω_0,_ ω_E_	56	−8406.51	0.165	0.078	5 vs. 0	0.422	>0.05
6. Two ratios: ω_0,_ ω_F_	56	−8406.62	0.164	0.237	6 vs. 0	0.187	>0.05

a See Figure 1B for branch labels.

b np, number of parameters.

c ω_Fix_ (ω_A_, ω_B_, ω_C_, ω_D_, ω_E_ and ω_F_) and ω_0_, are the ω ratios for branches A, B, C, D, E, F and other branches, respectively.

d The *P*-value of each test was multiplied by six (tested branches) to correct for multiple testing.

**Figure 3:**
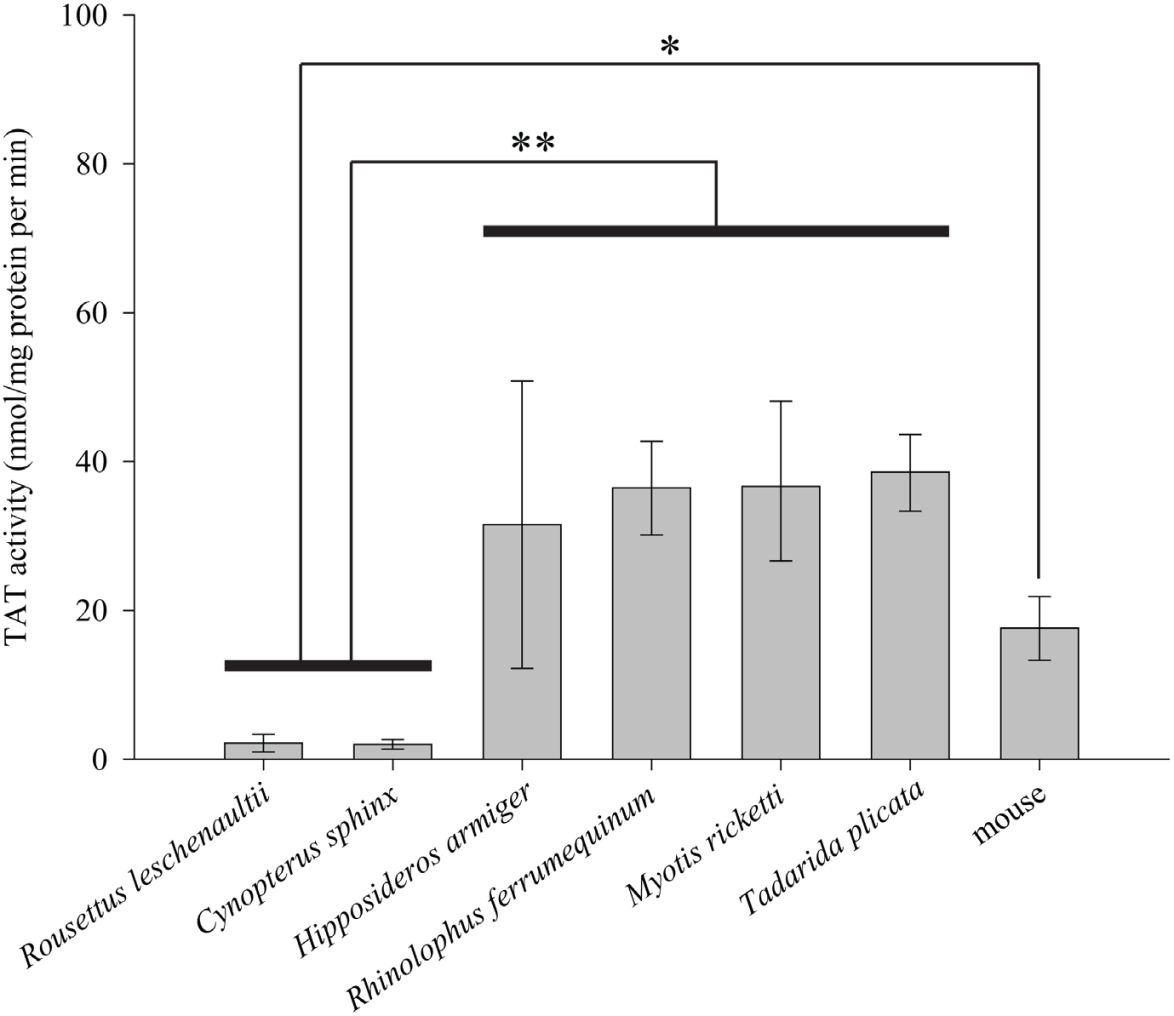
Hepatic tyrosine aminotransferase activities among six representative bat species and mice. The hepatic tyrosine aminotransferase activity was expressed as nmol of *p*-hydroxybenzaldehyde formed/mg protein per min (mean ± SD). For each species, at least three individuals were used for replication. Statistical significance (*P<0.05 and **P<0.001) is determined by one-way ANOVA, followed by Fisher’s least significant difference (LSD) *post hoc* tests.

To examine and compare the TAT activity between the Old World frugivorous bats and insectivorous bats, we conducted enzyme assays for two representative species of Old World frugivorous bats (*C. sphinx* and *Rousettus leschenaultii*), four insectivorous bats (*R. ferrumequinum*, *H. armiger*, *M. ricketti* and *Tadarida plicata*) and the omnivorous mouse outgroup. Our results showed that the TAT activities in the two Old World frugivorous bats were significantly lower than measured for the insectivorous bats (*P*<0.001, one-way ANOVA, LSD *post hoc* test) and mouse (*P*<0.05, one-way ANOVA, LSD *post hoc* test) (Figure 3). TAT activities in the two Old World frugivorous bats, *C. sphinx* (2.0±0.7) and *R. leschenaultii* (2.2±1.2), were almost 15- to 20-fold lower than those of the four insectivorous bats (31.5±19.3, 36.5±6.3, 36.7±11.5 and 38.6±5.0 for *H. armiger*, *R. ferrumequinum*, *M. ricketti* and *T. plicata*, respectively) and almost 10-fold lower than that of the omnivorous mouse (17.6±4.2) (Figure 3).

**Figure 4:**
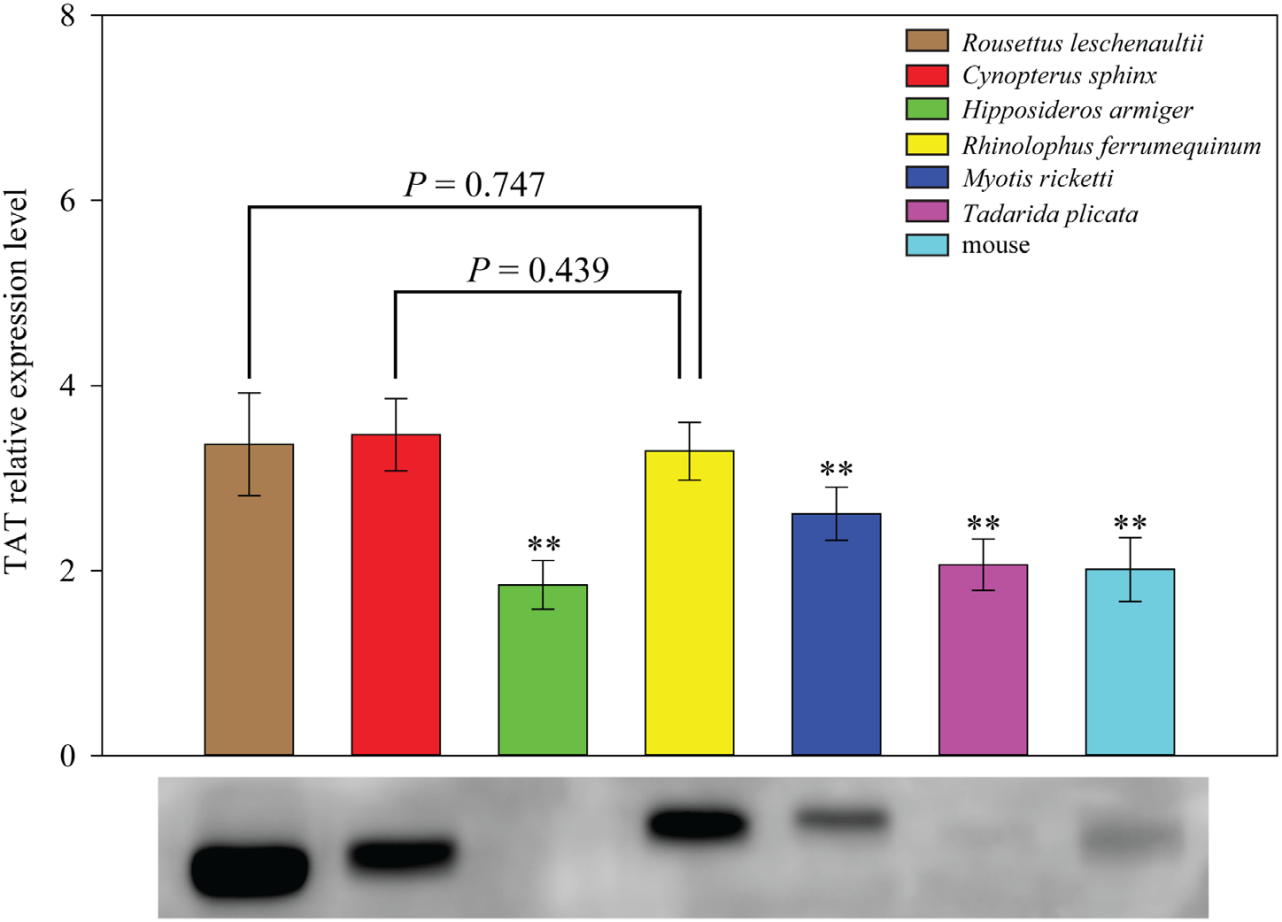
Expression pattern of tyrosine aminotransferase protein. Protein expressions of tyrosine aminotransferase in the livers of six representative bat species and mice were determined by Western blotting. For each species, three individuals were used for replication. Relative protein levels are presented as mean ± SD. The lowest level of a detectable protein is considered as 1. Western blotting against the tyrosine aminotransferase antibody on the PVDF membrane is shown beneath the histogram. Statistic significance (**P<0.001) is determined by one-way ANOVA, followed by Fisher’s least significant difference (LSD) *post hoc* tests.

To determine whether the extremely low TAT activity in the two Old World frugivorous bats is caused by a reduction in their hepatic TAT protein level, we performed Western blot assays to examine the TAT protein expression level in these six species of bats and the mouse. Western blots showed that the TAT protein levels in *C. sphinx* and *R. leschenaultii*, which exhibited extremely low TAT activities, are significantly higher than three of four examined insectivorous bats (*H. armiger*, *M. ricketti* and *T. plicata*) as well as the mouse (*P*<0.001, one-way ANOVA, LSD *post hoc* test), but not significantly different from that of the insectivorous bat *R. ferrumequinum* (*P* = 0.439 and 0.747, respectively, one-way ANOVA, LSD *post hoc* test) (Figure 4). These results strongly suggest that the low TAT activity in the two Old World frugivorous bats is not the result of reduced protein levels of hepatic TAT. Taken together, our results show that the *Tat* gene has undergone relaxed evolution that results in an enzyme that has lowered TAT enzymatic activity in Old World fruit bats.

## Discussion

Our phylogenetic reconstruction analyses based on the *Tat* nucleotide sequences revealed a conflicting phylogenetic signal between the reconstructed gene tree and the established species tree within the clade of Chiroptera. All three of our phylogenetic reconstruction methods recovered a gene tree in which yinpterochiropteran insectivorous bats are grouped with echolocating yangochiropteran insectivorous bats instead of with their closer related Old World fruit bats, although nodal support from Bayesian and maximum likelihood methods are not very high (Figure 1A). Conflicts between recovered gene trees and species trees can be caused by several reasons. First, it is possible that the phylogenetic conflict is caused by recombination in the *Tat* dataset, but this could be ruled out as our results of recombination detection tests showed no evidence for recombination. Second, phylogenetic conflicts may arise from the involvement of paralogous sequences in either the Old World fruit bats or insectivorous bats, due to a duplication of the *Tat* gene. The inclusion of paralogous gene in datasets readily leads to serious conflicts between reconstructed gene trees and species trees [58]. However, we argue that this possibility is also highly unlikely. To our knowledge, no evidence of a duplication of the *Tat* gene has been reported among mammals. In contrast, clear evidence has shown that the *Tat* gene is a single-copy gene in the rat [4]. We conducted genomic blast search analyses of the *Tat* gene through genomes of the eight mammal outgroups we studied and two bat species, *Pteropus vampyrus* and *Myotis lucifugus*, in the Ensembl database. However, no evidence of the *Tat* gene duplication was found in these mammals and the two bat species, indicating the *Tat* gene is also highly likely a single-copy gene in mammals. Moreover, if the observed phylogenetic conflicts were really caused by the involvement of paralogous sequences, then a single dominant phylogenetic signal should have been found in our data. However, some mixed phylogenetic signals, which support both the gene tree and the species tree, were found by our spectral analyses of site-wise log-likelihood values (Figure 2).

On the other hand, the possibility that the phylogenetic conflicts may have been caused by the convergent and/or parallel evolution of the *Tat* sequences among insectivorous bats should be considered. Similar phylogenetic conflicts between gene tree and species tree have recently been reported for many bat genes. Molecular evolutionary studies have revealed that many key genes involved in hearing have undergone convergent evolution among distantly-related echolocating bats in relation to their capacity for laryngeal echolocation and its associated high-frequency hearing [59], [60], [61], [62], [63]. Thus, it is reasonable to speculate that the observed clustering of the insectivorous bats may be caused by convergent evolution of the *Tat* gene in insectivorous bats, potentially as a consequence of their relatively high-protein content insect diets. However, the results of explicit tests for convergence (see Methods S1) showed that the posterior probabilities of convergence between the branches of yinpterochiropteran insectivorous bats and yangochiropteran insectivorous bats were not markedly higher than probabilities of divergence (Figure S7). These results indicate that there was no evidence for convergent evolution in the *Tat* sequence among insectivorous bats.

Alternatively, the phylogenetic conflict may have been caused by accelerated evolution of the *Tat* gene in the Old World fruit bats, which resulted from a relaxation of evolutionary constraints. Indeed, an elevated ω value in the ancestral branch for the Old World fruit bats was revealed by both our two-ratio model tests and the TestBranchDNDS tests. Consistent with these results, a total of 21 amino acid changes were shared by the four species of the Old World fruit bats (Figure S2). If the observed amino acid changes are a reflection of the accumulation of deleterious mutations caused by relaxed evolution, then a lesion of TAT enzymatic function should be expected in the Old World fruit bats. Our assays for TAT enzymatic activity showed that the TAT proteins from two Old World frugivorous bats have much lower levels of activity than those of insectivorous bats or the omnivorous mouse (Figure 3). However, other possibilities that may explain the changes in TAT activity in the Old World fruit bats also need to be discussed. First, it is possible that the unique amino-terminal structure of TAT in the Old World fruit bats (Figure S2), i.e., the presence of the 7 amino acid deletion (Figure S1B), may effects the activity of TAT. This possibility, however, could be ruled out, as biochemical studies have clearly shown that the amino-terminal sequences of TAT has no effects to the activity of TAT [64], [65]. Second, it is possible that the reduced TAT activity in Old World fruit bats are caused by a low vitamin B_12_ content in these bat species, as fruits, which fruit bats mainly fed on, are known to be devoid of vitamin B_12_ [66]. Deficiency of vitamin B_12_ adversely affects the regulation of TAT and results in abnormal TAT activity [67]. However, this possibility can also be ruled out as physiological studies has revealed that the vitamin B_12_ content of the serum, liver and kidney of a captured wild Old World frugivorous bat (*R. aegyptiacus*) are not significantly different from those of other mammalian species [68]. It is also possible that reduced TAT expression levels in these Old World frugivorous bats may cause the low level of TAT activity. However, this possibility is also be ruled out as our Western blot results clearly showed that the TAT protein levels in the two examined Old World frugivorous bats are higher than most of the examined insectivorous bats and the mouse. Thus, we argue that the reduced TAT activity in species of the Old World fruit bats is caused by relaxation of the evolutionary constraints on the *Tat* gene. Taken together, our results showed that the observed phylogenetic conflict between the reconstructed gene tree and the species tree is more likely to be caused by relaxed evolution on the *Tat* gene, which resulted in a reduction of activity of TAT in Old World fruit bats. However, since the species tree could not reject the reconstructed gene tree, thus we are unable to rule out the possibility that the phylogenetic conflict may also be caused by other unknown factors in addition to the relaxed evolution of the *Tat* gene in Old World fruit bats.

Thus, our results strongly supported our hypothesis that the *Tat* gene, which has undergone strong purifying selection across all insectivorous bats and other mammalian species, has surprisingly undergone a relaxation of its evolution in the Old World fruit bats. The relaxed evolution of the *Tat* gene in the Old World fruit bats is likely related to the high carbohydrate but low protein composition of their diets and changes in metabolism associated with these changes. With a carbohydrate rich diet, Old World fruit bats should fuel their metabolism mainly by carbohydrates [33], an energy source that can rapidly be absorb by passive paracellular pathway [26]. A reliance on carbohydrates should largely reduce their need for catabolism of amino acids for energy production compared with their insectivorous relatives. A recently published study has shown that genes encoding the other three enzymes (PAH, HPD and HGD) involved in phenylalanine and tyrosine catabolic pathway have also undergone relaxed evolution in the Old World fruit bats [37], although further studies on changes in enzymatic activity are needed to confirm this conclusion. It appears that the entire phenylalanine and tyrosine catabolic pathway has undergone a relaxation of their evolutionary constraints in bats of this lineage. However, in contrast to the enzymes PAH, HPD, HGD and FAH, all of which exhibited reductions in protein expression levels, hepatic TAT expression in Old World fruit bats is not reduced. Further studies are needed to determine why the *Tat* gene has undergone relaxed evolution but still retains a relatively high level of hepatic expression in Old World fruit bats. Since their diets have low protein content, fruit- and nectar-eating bats may preserve products generated from dietary proteins for protein synthesis rather than for catabolism [34], [35]. Thus it is possible that amino acids, and especially the essential amino acids such as tyrosine and phenylalanine which cannot be synthesized *de novo*, have escaped from catabolic pathway and are mainly used for protein synthesis. Moreover, tyrosine is also a precursor for melanin [69], catecholamines (adrenaline, noradrenaline and dopamine) [1], [70] and thyroid hormones [71], which are all involved in important physiological functions. Thus it is plausible that, in addition to protein synthesis, the restricted tyrosine content of the Old World fruit bats diet may be preserved for the use in the syntheses of these critical components. Taken together, diet associated changes in metabolism likely have largely reduced the importance of the tyrosine catabolic pathway in Old World fruit bats resulting in relaxed evolution of the *Tat* gene in bat species of this lineage.

Alignment of TAT sequences revealed a total of 21 amino acid changes shared by Old World fruit bats. To examine potential influence of these amino acid changes on the function of TAT in Old World fruit bats, we collected and mapped reported human amino acid mutations that cause tyrosinemia type II (R119W, C151Y, L201R, P220S, L273P, G362V and R443Q/W) [9], [10], [13], [14], [15] into the sequence alignment (Figure S2). None of 21 amino acid changes in the Old World fruit bats occurred at any of these amino acid positions (Figure S2), suggesting that additional amino acid positions affect the enzymatic function of TAT. In terms of amino acid properties, most of the 21 amino acid changes in the Old World fruit bats are conservative, however a few positions had more radical substitutions. At position 355 (Figure S2) the replacement of glycine (G), which is conserved across insectivorous bats and mammals to glutamate (E) may have dramatic effect on TAT function, considering the unique properties of glycine [72]. In addition, the leucine (L) to proline (P) replacement at position 171 is close to residue Phe169 (Figure S2), which is known to be involved in TAT cofactor pyridoxal-5′-phosphate pyridoxal ring stacking [73]. We also predicted whether these 21 amino acid changes in the Old World fruit bats will cause a neutral or deleterious effect on TAT protein function using PROVEAN (Protein Variation Effect Analyzer) v1.1.3 [74] based on comparisons with homologous amino acid sequences. Of these 21 amino acid changes, three amino acid substitutions were predicted to be deleterious mutations (V164A, L171P and V397F with scores of −3.221, −6.172 and −3.164, respectively). Further studies are needed to determine how these amino acid changes affect the enzymatic activity of TAT in Old World fruit bats, and the results of these studies may have implications for mutation screening for human tyrosinemia type II.

In contrast to the Old World fruit bats, relaxed evolution in the *Tat* gene was not found in the New World fruit bats, despite them evolving food habits quite similar to those of the Old World fruit bats. None of the amino acid changes observed in the Old World fruit bats was found to be shared by the two species of New World fruit bats (Figure S2), even in the species of *Artibeus lituratus*, belonging to the subfamily Stenodermatinae, which is predominantly frugivorous like the Old World fruit bats [20]. This difference may be explained in several ways. First, it may simply reflect the fact that the Old World fruit bats have evolved their frugivorous diet earlier than in New World fruit bats (at least 28 mya versus almost 20 mya) [21]. Thus, it is possible that New World fruit bats still retain an active TAT enzyme due to shorter period of relaxed evolution since their divergence from their insectivorous ancestor in the family Phyllostomidae [53]. Second, the evolutionary discrepancy between the Old World fruit bats and the New World fruit bats may be caused by differences in food availability when each group radiated. Food plant species diversity and plant food resource stability is much greater in the Neotropical region than in the Palaeotropical region [18], which may have enabled New World fruit bats to gain sufficient proteins by mixing different fruit species [75]. It is interesting to note that several other molecular evolutionary studies focused on genes involved in metabolism have revealed similar evolutionary discrepancies between the Old and New World fruit bats [30], [31], [32]. For example, the *Slc2a4* gene, which encodes the glucose transporter 4 (GLUT4) that plays a pivotal role in blood glucose homeostasis, has undergone adaptive changes only in the Old World fruit bats but not in the New World fruit bats [30]. Similarly, the gene *Agxt*, which encodes alanine-glyoxylate aminotransferase and plays an important role in plant-derived glyoxylate detoxifying, has also undergone positive selection only in the Old World fruit bats [31].

In conclusion, our studies, combining computational and experimental analyses, show that the *Tat* gene has undergone relaxed evolution in the Old World fruit bats, likely as a consequence of the evolution of the frugivourous diet and associated metabolic changes. This study provide unambiguous evidence of how food habits render effects on the genomic evolution in mammals [76], [77]. Further research is needed to delineate whether the genes encoding other aminotransferases, especially those for essential amino acids, have also undergone similar relaxed evolution in the Old World fruits and/or the New World fruit bats in response to the change in their diets.

## Supporting Information

Figure S1 **Schematic of** ***Tat*** **gene exons.** (A) A cartoon illustrating the primer pair designations for the *Tat* gene coding sequence amplification. Two pairs of primers are designed based on sequences spanning untranslated regions and exons. Primer sequences based on untranslated regions and exons are colored in black and red, respectively. Only exon 1 and exon 11 of the *Tat* gene are shown. (B) Genomic deletion of seven amino acids in four Old World fruit bats. Seven amino acids from positions 3 to 9 in the first exon of the *Tat* gene are deleted in the four Old World fruit bats. The human *Tat* gene sequence is used as reference.
(PDF)

Figure S2 **Alignment of the full amino acid sequences of the** ***Tat*** **gene from 28 mammals.** Twenty-one amino acid changes in the Old World fruit bats are highlighted by red and the changes are marked on the top of the alignment in blue. Seven amino acid mutations reported in humans with tyrosinemia type II are marked and indicated by asterisks (*), with mutation in red, on the top of the alignment. The first 23 amino acids of the amino-termini are not conserved and were removed from the dataset prior to phylogenetic reconstruction and molecular evolutionary analyses (see Materials and Methods) and are highlighted by the red box. Species belonging to the Old World fruit bats and the New World fruit bats are highlighted in green and blue, respectively.
(PDF)

Figure S3 **Variable amino acid sites in the sequences of the** ***Tat*** **gene from 28 mammals.** Variable sites from the alignment shown in Fig. S1 are shown. Species belonging to the Old World fruit bats and the New World fruit bats are highlighted in green and blue, respectively.
(PDF)

Figure S4 **Alignment of the full nucleic acid sequences and corresponding amino acids of the** ***Tat*** **gene from 28 mammals.** Twenty-one amino acid changes in the Old World fruit bats are highlighted by red and the changes are marked on the top of the alignment in red. Species belonging to the Old World fruit bats and the New World fruit bats are highlighted in green and blue, respectively.
(PDF)

Figure S5 **Neighbor-Joining (NJ) trees based on synonymous and nonsynonymous substitutions using the** ***Tat*** **gene nucleotide dataset removing codons for 21 shared amino acid changes in the Old World fruit bats, under the Kumar model.** (A) NJ tree based on synonymous substitutions. (B) NJ tree based on nonsynonymous substitutions. Codons corresponding to amino acids at positions 39, 51, 53, 65, 66, 83, 93, 94, 97, 164, 171, 232, 258, 263, 268, 307, 317, 355, 395, 397 and 398 are removed prior to phylogenetic reconstruction analyses. Values on the nodes are Neighbor-Joining bootstrap values. Branch lengths are based on the number of synonymous substitutions per synonymous site for (A) and the number of nonsynonymous substitutions per nonsynonymous site for (B).
(PDF)

Figure S6 **Results of the parametric bootstrapping analysis.** (A) Constrained species tree based on the *Tat* gene coding sequences as the null hypothesis (log *L* = −8528.75). Two hundred simulated datasets were generated based on this tree topology. (B) Unconstrained maximum-likelihood tree based on the *Tat* gene coding sequences as the alternative hypothesis (log *L* = −8528.52). (C) Distribution of the differences in maximum likelihood values under the assumption that the null hypothesis (constrained species tree) is correct. The observed difference of maximum likelihood values (–log Λ = 0.2319) is not significant (*P*-value = 0.295), thus, the constrained species tree could not be rejected by the unconstrained maximum-likelihood tree.
(PDF)

Figure S7 **Plot of total posterior probabilities of divergence versus convergence for all pairs of branches in the tree.** Pairwise comparison for the branches of Yinpterochiroptera echolocating bats versus branches of the Yangochiroptera bats, the ancestral branch of the Old World fruit bats versus the branches of Yinpterochiroptera echolocating bats, the ancestral branch of the Old World fruit bats versus branches of Yangochiroptera bats and the ancestral branch of the Old World fruit bats versus the ancestral branch of the New World fruit bats are highlighted.
(PDF)

Table S1 **Species analyzed in this study.**
(DOC)

Table S2 **Primers used for amplifying Tat coding sequences by PCR.**
(DOC)

Table S3 **Results of pairwise relative rate tests.**
(DOC)

Methods S1 (DOC)
