# Supplementary material for: Relaxed Evolution in the Tyrosine Aminotransferase Gene Tat in Old World Fruit Bats (Chiroptera: Pteropodidae)
Source: PLoS One. 2014 May 13;9(5):e97483. doi: 10.1371/journal.pone.0097483 (PMC4019583; doi:10.1371/journal.pone.0097483)
Supplement: Figure S1 — Schematic of Tat gene exons. (A) A cartoon illustrating the primer pair designations for the Tat gene coding sequence amplification. Two pairs of primers are designed based on sequences spanning untranslated regions and exons. Primer sequences based on untranslated regions and exons are colored in black and red, respectively. Only exon 1 and exon 11 of the Tat gene are shown. (B) Genomic deletion of seven amino acids in four Old World fruit bats. Seven amino acids from positions 3 to 9 in the first exon of the Tat gene are deleted in the four Old World fruit bats. The human Tat gene sequence is used as reference. (PDF) [file pone.0097483.s001.pdf]

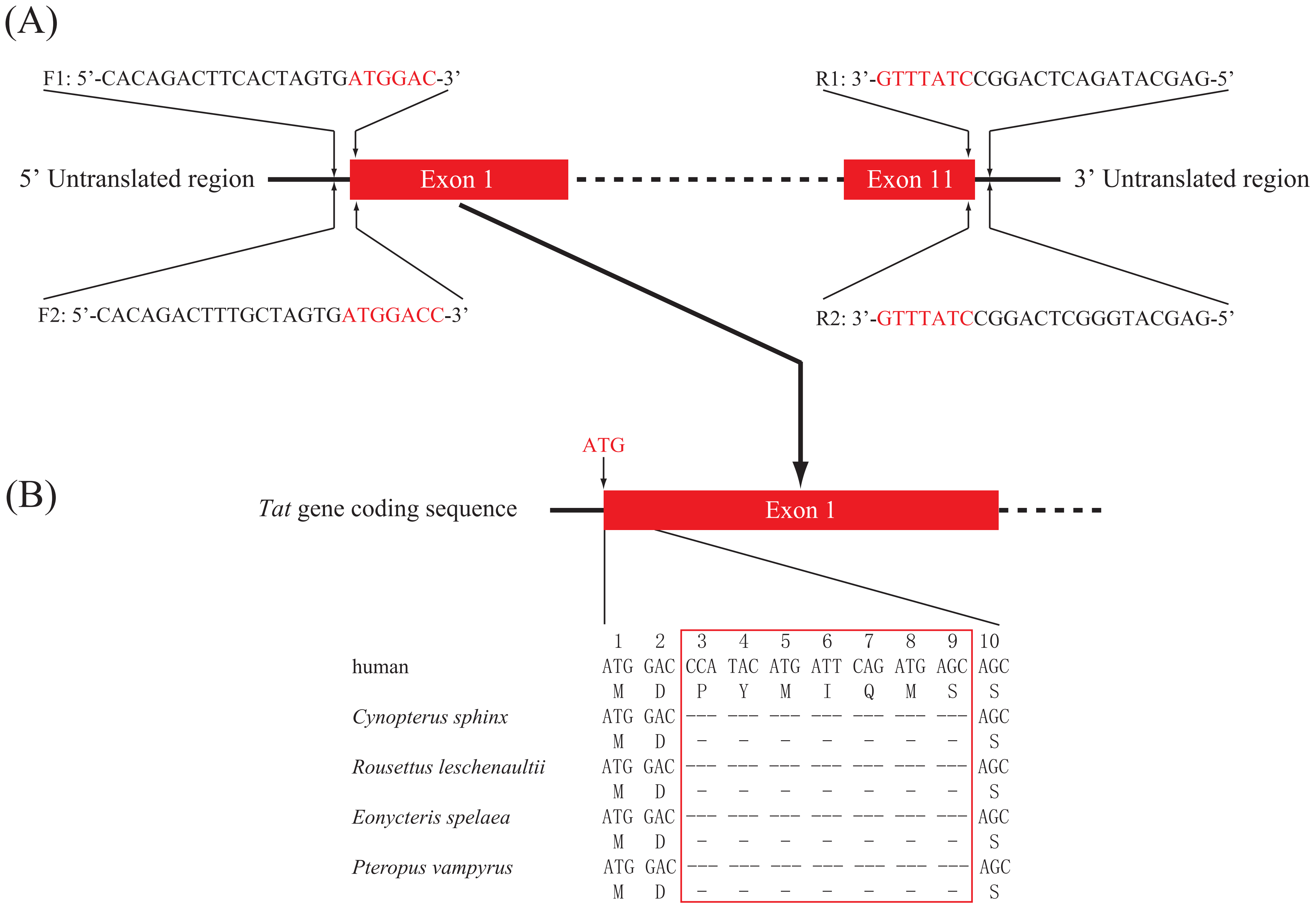

(A)
F1: 5'-CACAGACTTCACTAGTGATGGAC-3'
R1: 3'-GTTTATCCGGACTCAGATACGAG-5'
5' Untranslated region
Exon 1
Exon 11
3' Untranslated region
F2: 5'-CACAGACTTTGCTAGTGATGGACC-3'
R2: 3'-GTTTATCCGGACTCGGGTACGAG-5'
(B)
ATG
Tat gene coding sequence
Exon 1
1 2 3 4 5 6 7 8 9 10
human ATG GAC CCA TAC ATG ATT CAG ATG AGC AGC
M D P Y M I Q M S S
Cynopterus sphinx ATG GAC --- --- --- --- --- --- --- AGC
M D - - - - - - - S
Rousettus leschenaultii ATG GAC --- --- --- --- --- --- --- AGC
M D - - - - - - - S
Eonycteris spelaea ATG GAC --- --- --- --- --- --- --- AGC
M D - - - - - - - S
Pteropus vampyrus ATG GAC --- --- --- --- --- --- --- AGC
M D - - - - - - - S
