# Supplementary material for: Relaxed Evolution in the Tyrosine Aminotransferase Gene Tat in Old World Fruit Bats (Chiroptera: Pteropodidae)
Source: PLoS One. 2014 May 13;9(5):e97483. doi: 10.1371/journal.pone.0097483 (PMC4019583; doi:10.1371/journal.pone.0097483)
Supplement: Figure S3 — Variable amino acid sites in the sequences of the Tat gene from 28 mammals. Variable sites from the alignment shown in Fig. S1 are shown. Species belonging to the Old World fruit bats and the New World fruit bats are highlighted in green and blue, respectively. (PDF) [file pone.0097483.s003.pdf]

					11111	1111111111	1111111111	1111111111	1111112222	2222222222
	1122222233	3333333344	4445555666	6677777888	9999900111	1222222223	3334444555	6777777888	8899990000	0111222333
	0312578901	2345678902	4790138123	5602358348	1345716025	7123567890	6892589137	4013569123	4535690123	5034678258
human	SNHVGRSSVP	GKMKGRKARS	RDAKKFADNM	VKNTMSIVFP	PVTQMLYASF	SEIAYYHCPE	KVISSIDCAN	VSLKLAMIEV	KLSEIKQLEY	IAIVSKRKAA
mouse	VSRVGRSSVQ	GRAKGRKARN	RDSNKFADNM	VKNTVSIVFP	PVTQMLYASY	SEVAYYHCPE	KVISSIECAN	ISLRLAMIEV	KLSEIKQLES	IAVVSKRKAE
rat	VSHVGRNSVQ	GRKKGRKARD	RDSNKFADNM	VQNTVSIVFP	PVTQMLYASY	SEVAYYHCHE	KVISSIECAN	ISLRLAMIEV	KLSEIKQLES	IAVVSKRKAE
cow	DSHVARSSVL	GKVKSRKARS	RDSNKFADNM	VKNTMAIVFP	PVTQMLFVSY	SEVAYYHCPE	KVISSIECAN	VSLRLAMIEV	KLNEIKQLES	IVIVSRRKAA
dog	SNHVGRSSLP	GKMKGRKARS	RDSNKFADSS	VKNATAIVFP	PVTQMLYASY	SEIAYYHRPE	KVISSIECAN	VSLRLAMIEV	KLSEIKQLES	IAIVSKSKAA
pig	GNHVARIPVP	GKMKGRKARS	RDSNKCADSM	VKNTTSIVFP	QVTQMLYASY	SEVAYYHCPE	KVISSIECAN	VPLRLAMIEV	KLSEIKHLES	IAVVSRNKAT
panda	SNHVGRSSLP	GKMKGRKARS	KDSNKFADSM	VKNTMAIVFP	PVTQMLYASY	SEIAYYHRPE	KVISSIECAN	VSLRLAMIEV	KLSEIKQLES	IAIVSKSKAA
horse	GNHVSRSSAP	GKMKGRKARS	RDSNKFADNM	VKNTMAIVFS	PVTQLVYATY	SEIAYYHHPE	KVISSIECAN	VALRLAMIEV	KCSEIKQVES	VAIVSKSKAA
Cynopterus sphinx	SDHMNRSSAP	EKVKGRKPRS	KDANRYADSM	MEDTTTILSP	PIIQLLYASY	SEVAYFRCPE	KVISSVECAN	ASPRLAMIKI	KLSEIKQLES	IAIVSKSDAA
Rousettus leschenaultii	SDHVNRSSVP	EKVKGRQPGS	RDANRYADNM	LEDPMSLLFP	PIIQLLYASY	SEIAYYHHPK	KVICSIECAN	ASPRHAMIEL	KLSEIKQMES	IAIVSKSDAA
Eonycteris spelaea	SDHVSRSPVP	EKVKGRKTES	RDANRYADSM	MEDTTAILFP	PIIQLLYASY	SEVAYYHHPK	KVICSIECTN	ASPRLALVEV	KLYEIEQLES	IAIVSKSDAA
Pteropus vampyrus	SDHINRSSVS	EKVKGRKPRS	RDANRYADSM	MEDTTPILCP	PIIKLLYASY	SEIAYYHRPE	KVISSIECAN	ASPRLALIEI	KLSEVKQLES	IAIVSKSDAA
Rhinolophus ferrumequinum	GNHVGKNSKQ	EKGKGKKARS	RDANNFTDNM	VKHTMAIVFP	PVTQMLYASY	SEIAYYHCPE	NVISGIECAN	LSLRLAMIEV	KLSEIKQLKS	IAIVSKNKAA
Rhinolophus pusillus	GNHVGKNSKQ	EKVKGRKARS	KDSNKFTDNM	VKDAMAIVFP	PVTQMLYASY	SEIAYYHCPE	KVISSIECAN	ISLRLAMIEV	KLSEIKQLES	IAIVSTNKAA
Hipposideros armiger	SNHVGKNSKQ	GKVKGRKARS	KESNKFTDGM	VKNTMSIVFP	PVTQMLYASY	SEVAYYHCPE	KVISSIECAN	VSLRLAMIEV	KLSKIKQLEA	IAIVSKSKAA
Hipposideros pratti	SNHVGKNSKQ	GKVKGRKARS	KESNKFTDGM	VKNTMSIVFP	PVTQMLYASY	SEVAYYHCPE	KVISSIECAN	VSLRLAMIEV	KLSKIKQLEA	IAIVSKSKAA
Megaderma lyra	GTHVGRSSMP	GKVKGRKARS	KDSNKFTDSM	VKDAMAIVFP	PVTQMLYASY	SEIAYYHCPE	KIISSIECAN	VSLKLAMIEV	KLSEIKQLES	IAIVSKSKAA
Megaderma spasma	GTHVDRSSMP	GKARGRKARS	KDSNNFTDSM	VKDAMAIVFP	PVTQMLYASY	SEIAYYHCPE	KVISSIECAN	VSLKLAMIEV	KLSEIKQLES	IAIVSKSKAA
Myotis ricketti	DDHVGRSSTP	GKAKGRKARS	KDSNNFADSM	VKDTMSIVFP	PVTQMLYASY	SEVAYYHCPE	KVISSIECAN	VSLRLAMIEI	KLSEIKQLES	IAIVSRSKAA
Pipistrellus abramus	GDHVGRSSTL	GKAKGRKARS	KDANNFADSM	VKDTMSIVFP	PVTQMLYASF	SEVAYYHCPE	KVISSIECAN	VSLRLAMIEV	KLSEIKQLES	IAIVSRSKAA
Scotophilus kuhlii	GDHVGRSSTP	GKGRGRKARS	KDSNNFADSM	VKDAMSIVFP	PVTQMLYASY	SEVAYYHCPE	EVISSIECAN	VSLRLAMIEV	KLSEIKQLES	IAIVSRSKAA
Miniopterus fuliginosus	GNHVGRSSMP	GKAKGRKARS	KDSNKFAESM	VKDTMSIVFP	PVTQMLYASY	SEIAYYHCPE	KVISSIECAN	VSLRLAMIEI	KLSEAKQLES	IAIVSRSKAA
Tadarida plicata	GPPVGRSSMP	GKAKGRKARS	KESKNFADSM	VKDTMSIVFP	PVIQMLYASY	SEIAYYHCPE	KVISSIECAN	VSLRLAMIEI	KLSEIKQLES	IAIVSRSKAA
Mormoops megalophylla	GSHVGKNSLP	GKGKGRKARS	KDSNNFADSM	VKDPMSIVFP	PVTQMLYASY	SEIAYYHCPE	KVISSIECAN	VSLRLALVEV	RFSEIKQLES	IAIIRRSKAA
Pteronotus parnellii	GNHVGRNSTL	GKGKGRKARS	KDSNNFADSM	VKDTMSIVFP	PVTQMLYASY	SEIAFYHCPE	KVISSIESAN	VSLRLAMIEV	KLSEVKQLES	IAIVSRSEAA
Desmodus rotundus	GSHVGKNSQP	GKGKGRKARS	KDANNFDDGM	VKAAMSIVFP	PVIQMLYASY	SEIVYHHCPE	KVISSIECAN	VSLRLTMIEI	KLSEIKQLES	IAVVSRSKAA
Leptonycteris yerbabuena	RSHVGKNSLP	GKGKGRKARS	KDANNFADNM	VKDTMSIVFP	PVTQMLYASY	CDIAYYHCPE	KVLSSIECAN	VSLRLAMIEV	KLSEIKQLES	IAIVSRSKAA
Artibeus lituratus	RSHVGKNSLP	GKGKGRKARS	KAANNFADSM	VKDTMSIVFP	PVTQMLYASY	SEIAYYHCPE	KVISSIECAS	VSLRLAMIEV	KLSEIKQLES	IAIVSRSKTA

	2222222222	2222222222	2333333333	3333333333	3333333333	3333333333	3444444444	4444444444	44
	3444555566	6666778999	9001111223	3333444444	5556666667	7778889999	9000001112	2222334444	44
	9015147801	3589499348	9670137292	5679015789	0580467891	4792780157	8134781361	2467260346	89
human	RQCLGVDCYE	LTTDSALLIR	DRDVKSLISC	PGEYHNFKSN	AGAIRVRPSA	LGEHENVERV	ASHCATEPIT	VEMMSEQHCE	SQ
mouse	RQCLGVDCYE	MTTNSALLIR	DRDVKSLISQ	PQEYQDFKSN	AGSIQVRPSA	LGEHENVERI	ASHCATEPFT	VEMMSEQHCE	SQ
rat	RQCLGVDCYE	LNTNSALLIR	DRDVKSLISQ	PQEYHDFKSN	AGAIQVRPSA	LGEHENVERI	AAHCATEPFT	VEMMSEQHCE	SQ
cow	RQCLGVDSFE	LTSKSAMLIR	DRDTKSLLSC	PRVYHNFKSN	AGAIRIRPSA	LGEHENVEQV	ASHCATEPFT	VEMMSEQHCE	SQ
dog	RQCLGVDSFE	LTSNSALLIR	DRDVKSLISR	PQEYQNFKSN	AGAIRVRPSA	LGEHENVERV	ASHCATEPFT	VKMMSELHCE	SQ
pig	RQCLGVDSFE	LTSNSALLIR	DRDVKSLLSR	PQEYHNFKTN	AGAVRIRPCA	LGEQENVERV	ASHCGMERFT	VKMMSEQQCE	TQ
panda	KQCLGVDSFE	LTSNSALLIR	DRDVKSLISH	PQEYHNFKSN	AGAIQVRPSA	LGEHENVERV	ASHCATEPFT	VKMMSELHCE	SQ
horse	RQCIGVDSFE	LTSNSALLIR	DRDVKSLINR	PQEYHNLKSN	AGAIRVRPSA	LGEHENVERV	ASHCGTELFT	VKMMSEQHCE	SQ
Cynopterus sphinx	RQCVGVDTFE	ITRSCTLLIG	DRNVKSMLSR	PQEYHNFKSS	AVSIRVRPSA	LEEHENVERF	VSHCAMEPFA	VKTISEQHHE	IQ
Rousettus leschenaultii	RKYLGVDTFE	ITRNTALLIR	DRNVKCMISR	PQEYHNFKSN	AEAIRVRSSA	IEQHENVEQF	VSQCAKEPFT	VKMMSEQHLE	SQ
Eonycteris spelaea	RKYLGVDTFV	ITRNTALFIK	DRNVRCMISR	PQEYHNFKSS	AEAIRVHSSA	LEEHENVEQF	VSHCAKEPFT	VKMMSEQHHE	TQ
Pteropus vampyrus	RQCLGMDTFE	ITHNSALLIG	DPNVKSMVSR	PQEYRNVKSS	AESIRVRPSA	LEEHENVEQF	VSLCAIEPFA	VKMISEQHHE	IQ
Rhinolophus ferrumequinum	RQCLGVDSFE	LTSNSALLIK	DRDVKSLISH	PQEYHNFKSN	AGAIRVRPSA	LEEHENVERV	ASHCATEPFT	VEMMSEKHCE	SQ
Rhinolophus pusillus	RQCLGVDSFE	LTSNSALLIK	DRDVKSLISH	PQEYHNFKSN	AGAIRVRPSA	LEEHKNVERV	ASHCATEPFT	VEMMSEKHCE	SQ
Hipposideros armiger	RQCLGVDSFE	LNTNSALLIR	NRDVKSLLNR	PQEYHNFKSN	AGSIRVRPSA	LEEHENVERV	ASHCATEPFT	VEMMGEQHCE	SQ
Hipposideros pratti	RQCLGVDSFE	LNTNSALLIR	NRDVKSLLNR	PQEYHNFKSN	AGSIRVRPSA	LEEHENVERV	ASHCATEPFT	VEMMGEQHCE	SQ
Megaderma lyra	RQCLGVDSFE	LTSNSALLIR	DRDVKSLISR	PQEYHNFKSN	AGAIRVRPSA	LEEHKDVERV	ASHCATEPFT	VEMMSERHGE	SQ
Megaderma spasma	RQCLGVDSFE	LTGHSALLIR	DRDVKSLISR	PQEYHNFKSN	AGAIRVRPSA	LEEHKDVERV	ASHCATEPFT	VEMMSERHGE	SQ
Myotis ricketti	RQCLGVGSFE	LTSNSALLIR	DRDVKSLISR	PQEYHDFKSN	AGAIRVRPSA	LEEHENVERV	ASHCATEPFT	VEMISEQHCE	SP
Pipistrellus abramus	RQCLGVGSFE	LTTNSALLIR	DRDVKSLISR	PQEYHDFRSN	AGAIRVRPSA	LEEHENVERI	ASHCATEPFT	VEMMSEQHHE	SP
Scotophilus kuhlii	RQCLGVGSFE	LTSNSALLIR	DRDVKSLISR	PQEYHDFKSN	AGAIRVRPSA	LEEHENVERV	ASHCATEPFT	VEMMSEQHCE	SP
Miniopterus fuliginosus	RQCLGVDSFE	LTSNSALLIK	GRDVKSLISH	TQEFQDFKSN	AGAIRVRPSA	LEEHENVERV	ASHCATEPFT	VEMMSEQHSE	SH
Tadarida plicata	RQCLGVDSFE	LTSNSALLIR	DRDVKSLISR	PQEYHNFKSN	AGAIRVRPSA	LEEHENVDRV	ASHCATEPFT	VEMMSEQHCE	SQ
Mormoops megalophylla	RQCLAVDSFE	LNSDSALLIR	DRDVKSLISR	PREYHNFKSS	AGAIRIRPLA	LEEHENEERI	ASQLAKEPFT	VEMMSEQHCE	SQ
Pteronotus parnellii	KQCLGVDSFE	LNSNSALLIR	DRDVKSLISR	PQEYHNFKSN	AGAVRVRPSA	LEQHENVERV	ASHCATEPFT	VEMMSEQHCE	SQ
Desmodus rotundus	RQCLGVDSFE	LSSNSAMLIR	DRDVESLISR	PQEYQNFKSN	GGAIRVRPSA	LEEHGNVERV	ASHCATGPFT	VEMISKQHSE	GQ
Leptonycteris yerbabuena	RQCLGVNSFE	LNSNSALLVR	DRDVKSLISR	PQEYHNFKSN	AGAIRVRPST	LEEHENVERV	ASHCATEPFT	VKMMSEQHCE	SQ
Artibeus lituratus	RQCLGVDSFE	LNSNSALLIR	DRDVKSLISR	PQEYHNFKSN	AGAIWVRPSA	LKEHENVERV	ASHCATEPFT	AEMMSEQHCA	SQ
