# Supplementary material for: Relaxed Evolution in the Tyrosine Aminotransferase Gene Tat in Old World Fruit Bats (Chiroptera: Pteropodidae)
Source: PLoS One. 2014 May 13;9(5):e97483. doi: 10.1371/journal.pone.0097483 (PMC4019583; doi:10.1371/journal.pone.0097483)
Supplement: Figure S4 — Alignment of the full nucleic acid sequences and corresponding amino acids of the Tat gene from 28 mammals. Twenty-one amino acid changes in the Old World fruit bats are highlighted by red and the changes are marked on the top of the alignment in red. Species belonging to the Old World fruit bats and the New World fruit bats are highlighted in green and blue, respectively. (PDF) [file pone.0097483.s004.pdf]

A39P

human	ATG GAC CCA TAC ATG ATT CAG ATG AGC AGC AAA GGC AAC CTC CCC TCA ATT CTG GAC GTG CAT GTC AAC GTT GGT GGG AGA AGC TCT GTG CCG GGA AAA ATG AAA GGC AGA AAG GCC AGG	[120]
	M D P Y M I Q M S S K G N L P S I L D V H V N V G G R S S V P G K M K G R K A R	[40]
mouse	ATG GAC TCC TAC GTA ATC CAG ACG AAT GTC AAT GAC AGC CTA CCT TCA GTG CTG GAT GTT CGC GTC AAT ATT GGC GGG AGA AGC TCG GTG CAA GGG AGA GCG AAG GGC AGG AAG GCC AGG	[120]
	M D S Y V I Q T N V N D S L P S V L D V R V N I G G R S S V Q G R A K G R K A R	[40]
rat	ATG GAC TCC TAC GTG ATT CAG ACG GAT GTC GAC GAC AGC TTG TCC TCA GTT CTG GAT GTG CAT GTC AAT ATT GGT GGG AGA AAC TCG GTA CAA GGA AGA AAG AAA GGC AGG AAG GCC AGA	[120]
	M D S Y V I Q T D V D D S L S S V L D V H V N I G G R N S V Q G R K K G R K A R	[40]
cow	ATG GAC CCA TAT GTG ATT CAG ATG CAG GAT CAC GGC AGC CTC CCC TCA GTT CTG GAT GTG CAT GTC AAC GTT GCT GGG AGA AGC TCT GTG CTG GGA AAA GTG AAA AGC AGG AAG GCC AGA	[120]
	M D P Y V I Q M Q D H G S L P S V L D V H V N V A G R S S V L G K V K S R K A R	[40]
dog	ATG GAC CCA TAT GTG ATT GAG ATG AAC AGC AAC AGC AAC CTC CCC TCA GTC CTG GAT GTG CAT GTC AAT ATT GGT GGG AGA AGC TCC TTG CCA GGA AAA ATG AAA GGC AGG AAG GCC AGA	[120]
	M D P Y V I E M N S N S N L P S V L D V H V N I G G R S S L P G K M K G R K A R	[40]
pig	ATG GAC CCC TAC GTG ATT CAG ATG CAC GGC CAT GGC AAC CTC CCC TCC GTT CTG GAT GTG CAT GTC AAC ACT GCT GGT AGA ATC CCT GTG CCA GGA AAA ATG AAA GGC AGG AAG GCC AGA	[120]
	M D P Y V I Q M H G H G N L P S V L D V H V N T A G R I P V P G K M K G R K A R	[40]
panda	ATG GAC CCG TAT GTC ATT CAG ATG AAC AGC AAC AGC AAC CTC CCC TCC GTT CTG GAT GTG CAT GTC AAC ATT GGT GGG CGA AGC TCT TTG CCG GGA AAA ATG AAA GGC AGG AAG GCC AGA	[120]
	M D P Y V I Q M N S N S N L P S V L D V H V N I G G R S S L P G K M K G R K A R	[40]
horse	ATG GAC CCA TAC GTG ATT CAG ATG ACA GGC AGC GGT AAC CTG CCC TCA GTT CTG GAC ATG CAT GTC AAC ATC AGT GGG AGA AGC TCT GCA CCA GGA AAA ATG AAA GGC AGG AAG GCC AGA	[120]
	M D P Y V I Q M T G S G N L P S V L D M H V N I S G R S S A P G K M K G R K A R	[40]
Cynopterus sphinx	ATG GAC --- --- --- --- --- --- --- AGC AAT GGT GAC CTC CCC TCC ATT CTG GGT GTG CAC ATG CAT ATC AAT GGA AGA AGC TCC GCG CCT GAA AAA GTG AAA GGC AGA AAG CCC AGA	[120]
	M D - - - - - - - S N G D L P S I L G V H M H I N G R S S A P E K V K G R K P R	[40]
Rousettus leschenaultii	ATG GAC --- --- --- --- --- --- --- AGC GAT GGT GAC CTC CCC TTC CTT CTG GAT GTG CAT GTC AGC GTC AAC GGA AGA AGC TCC GTG CCT GAA AAA GTG AAA GGC AGA CAG CCC GGA	[120]
	M D - - - - - - - S D G D L P F L L D V H V S V N G R S S V P E K V K G R Q P G	[40]
Eonycteris spelaea	ATG GAC --- --- --- --- --- --- --- AGC AGT AGT GAC CTC CCC TTC CTT CTG GAT GTG CAT GTC AGT GTC AGT GGA AGA AGC CCT GTG CCT GAA AAA GTG AAA GGC AGA AAG ACC GAA	[120]
	M D - - - - - - - S S S D L P F L L D V H V S V S G R S P V P E K V K G R K T E	[40]
Pteropus vampyrus	ATG GAC --- --- --- --- --- --- --- AGC AAA GGT GAC CTC CCC TCC GTT CTG GAT GTG CAC ATA AAC ATT AAT GGA AGA AGC TCC GTG TCT GAA AAA GTG AAA GGC AGA AAG CCC AGA	[120]
	M D - - - - - - - S K G D L P S V L D V H I N I N G R S S V S E K V K G R K P R	[40]
Rhinolophus ferrumequinum	ATG GAC CCG TAC GTG ATC CAG ATG AAC GGC AAC AGC AAC CTC CCC TCC GTT CTG GAT GTT CAT GTT AAC ATC GGT GGG AAA AAC TCC AAG CAG GAA AAA GGG AAA GGC AAG AAG GCC AGG	[120]
	M D P Y V I Q M N G N S N L P S V L D V H V N I G G K N S K Q E K G K G K K A R	[40]
Rhinolophus pusillus	ATG GAC CCG TAC ATG ATC CAG ATG AAC GGC AAC GGC AAC CTC CCC TCC GTT CTG GAT GTT CAT GTT AAC ATC GGT GGG AAA AAC TCC AAG CAG GAA AAA GTG AAA GGC AGG AAG GCC AGG	[120]
	M D P Y M I Q M N G N G N L P S V L D V H V N I G G K N S K Q E K V K G R K A R	[40]
Hipposideros armiger	ATG GAC CCA TAC GTG ATC CAG ATG AAC AGC AAT GGC AAC TTC CCC TCT GTG CTG GAT GTT CAT GTC AAC ATC GGT GGG AAA AAC TCC AAG CAG GGG AAA GTG AAA GGC AGG AAG GCC AGA	[120]
	M D P Y V I Q M N S N G N F P S V L D V H V N I G G K N S K Q G K V K G R K A R	[40]
Hipposideros pratti	ATG GAC CCA TAC GTG ATC CAG ATG AAC AGC AAT GGC AAC TTC CCC TCT GTG TTG GAT GTT CAT GTC AAC ATC GGT GGG AAA AAC TCC AAG CAG GGG AAA GTG AAA GGC AGG AAG GCC AGA	[120]
	M D P Y V I Q M N S N G N F P S V L D V H V N I G G K N S K Q G K V K G R K A R	[40]
Megaderma lyra	ATG GAC CCC TAT GTG ATC CAG ATG AAT GGC --- --- ACC CTC CCC TCC GTT CTG GAT GTG CAC GTC AAC ATC GGT GGG AGA AGC TCC ATG CCC GGA AAA GTG AAA GGC CGG AAG GCC AGA	[120]
	M D P Y V I Q M N G - - T L P S V L D V H V N I G G R S S M P G K V K G R K A R	[40]
Megaderma spasma	ATG GAC CCC TAT GTG ATC CAG ATG AAT GGC --- --- ACC CTC CCC TCC GTT CTG GAT GTG CAC GTC AAC GTC GAT GGG AGA AGC TCC ATG CCC GGA AAA GCG AGA GGC CGG AAG GCC AGA	[120]
	M D P Y V I Q M N G - - T L P S V L D V H V N V D G R S S M P G K A R G R K A R	[40]
Myotis ricketti	ATG GAC CCA TAC GTG ATC GAG ATG AAT GAC AGC GGC GAC CTC CCC TCC GTT CTG GAT GTG CAT GTC AAC GTC GGT GGG AGA AGC TCC ACG CCA GGA AAA GCG AAA GGC AGG AAG GCC CGG	[120]
	M D P Y V I E M N D S G D L P S V L D V H V N V G G R S S T P G K A K G R K A R	[40]
Pipistrellus abramus	ATG GAC CCC TAC GTG ATC GAG ATG AAC GGC AGC GGC GAC --- --- --- --- --- --- --- CAT GTC AGC GTC GGC GGG AGG AGC TCC ACG CTG GGG AAA GCG AAA GGC CGG AAG GCC CGG	[120]
	M D P Y V I E M N G S G D - - - - - - - H V S V G G R S S T L G K A K G R K A R	[40]
Scotophilus kuhlii	ATG GAC CCA TAC ATG ATC GAG ACG AAC GGC AGC GGC GAC CTC CCC TCT GTG CTG GAT GTG CAC GTC --- --- GGC GGG AGA AGC TCC ACG CCA GGG AAA GGG AGA GGC AGG AAG GCC CGG	[120]
	M D P Y M I E T N G S G D L P S V L D V H V - - G G R S S T P G K G R G R K A R	[40]
Miniopterus fuliginosus	ATG GAC CCA TAC GTG ATT CAG ATG AAC GGC AGC GGC AAC CTC CCC TCC GTT GTG GAT GTG CAT GTC AAC ATC GGG GGG AGA AGC TCC ATG CCG GGA AAA GCG AAA GGC AGG AAG GCC AGG	[120]
	M D P Y V I Q M N G S G N L P S V V D V H V N I G G R S S M P G K A K G R K A R	[40]
Tadarida plicata	ATG GAC CCA TAC GTG ATT CAG ATG AAT GGC AAG AGC CCC CTC CCC TCT GTT CTG GAT GTG CCT GTC AAC ATC GGT GGG AGA AGC TCC ATG CCA GGA AAA GCG AAA GGC AGG AAG GCC AGG	[120]
	M D P Y V I Q M N G K S P L P S V L D V P V N I G G R S S M P G K A K G R K A R	[40]
Mormoops megalophylla	ATG GAC CTG TAC GTG ATT CAG ATG AAT GGC AAG GGC AGC CTG CCC CCC GTC CTG GAC GTG CAT GTC AAC ATC GGT GGG AAA AAC TCT CTG CCA GGA AAA GGG AAA GGC AGG AAG GCC AGA	[120]
	M D L Y V I Q M N G K G S L P P V L D V H V N I G G K N S L P G K G K G R K A R	[40]
Pteronotus parnellii	ATG GAC CCA TAC CTG ATT CAG ATG AAT GGC ATG GGC AAC CTG CCC TCT GTG CTG GAC GTG CAT GTC AAC GTT GGT GGG AGA AAC TCT ACG CTA GGA AAA GGC AAA GGC AGG AAG GCC AGA	[120]
	M D P Y L I Q M N G M G N L P S V L D V H V N V G G R N S T L G K G K G R K A R	[40]
Desmodus rotundus	ATG GAC CCA TAC GTG ATT CAG ATG AAC GGG AAG GGC AGC CTG CCC TCT CTC CTG GAC ATG CAT GTC AAC GTT GGT GGG AAG AAC TCT CAG CCA GGA AAA GGG AAA GGC AGG AAG GCC AGA	[120]
	M D P Y V I Q M N G K G S L P S L L D M H V N V G G K N S Q P G K G K G R K A R	[40]
Leptonycteris yerbabuena	ATG GAC CCA TAT GTG ATT CAG ATG AAC AGG AAG GGC AGC CTG CCC TCT CTC CTG GAC ATG CAT GTC AAT GTC GGT GGG AAG AAC TCT CTG CCA GGA AAA GGG AAA GGC AGG AAG GCC AGA	[120]
	M D P Y V I Q M N R K G S L P S L L D M H V N V G G K N S L P G K G K G R K A R	[40]
Artibeus lituratus	ATG GAC CCA TAC GTG ATT CAG ATG AAC AGG AAG GGC AGC CTG CCA TCT CTC CTG AAC GTG CAC GTC AAC ATC GGT GGG AAG AAC TCT CTG CCA GGA AAA GGG AAG GGC AGG AAG GCC AGA	[120]
	M D P Y V I Q M N R K G S L P S L L N V H V N I G G K N S L P G K G K G R K A R	[40]

K51R F53Y V65M K66E

human	TGG TCT GTG AGG CCC TCA GAC ATG GCC AAG AAA ACT TTC AAC CCC ATC CGA GCC ATT GTG GAC AAC ATG AAG GTG AAA CCA AAT CCA AAC AAA ACC ATG ATT TCC CTG TCC ATT GGG GAC	[240]
	W S V R P S D M A K K T F N P I R A I V D N M K V K P N P N K T M I S L S I G D	[80]
mouse	TGG AAT GTG AGG CCC TCT GAC ATG TCC AAT AAA ACC TTC AAT CCC ATC CGA GCC ATT GTG GAC AAC ATG AAG GTG AAG CCG AAT CCG AAC AAA ACC GTG ATC TCT CTG TCA ATT GGG GAC	[240]
	W N V R P S D M S N K T F N P I R A I V D N M K V K P N P N K T V I S L S I G D	[80]
rat	TGG GAC GTG AGA CCC TCT GAC ATG TCC AAT AAG ACC TTC AAT CCC ATC CGA GCC ATC GTG GAC AAC ATG AAG GTG CAG CCC AAT CCG AAC AAG ACC GTG ATT TCT CTG TCA ATT GGG GAC	[240]
	W D V R P S D M S N K T F N P I R A I V D N M K V Q P N P N K T V I S L S I G D	[80]
cow	TGG TCC GTG AGG CCC TCG GAC ATG TCC AAC AAA ACT TTC AAT CCC ATC CGG GCC ATT GTG GAC AAC ATG AAG GTG AAG CCA AAT CCA AAC AAG ACC ATG ATT GCT TTG TCG ATT GGG GAC	[240]
	W S V R P S D M S N K T F N P I R A I V D N M K V K P N P N K T M I A L S I G D	[80]
dog	TGG TCT GTG AGG CCT TCA GAC ATG TCT AAC AAA ACT TTC AAC CCC ATC CGG GCC ATC GTG GAC AGC TCG AAG GTG AAG CCA AAT CCA AAT AAA GCC ACA ATT GCT CTG TCT ATT GGC GAC	[240]
	W S V R P S D M S N K T F N P I R A I V D S S K V K P N P N K A T I A L S I G D	[80]
pig	TGG TCC GTG AGG CCC TCG GAC ATG TCC AAC AAG ACT TGC AAT CCC ATC CGG GCC ATC GTG GAC AGC ATG AAG GTG AAA CCG AAC CCA AAC AAG ACC ACA ATA TCT CTG TCA ATT GGG GAC	[240]
	W S V R P S D M S N K T C N P I R A I V D S M K V K P N P N K T T I S L S I G D	[80]
panda	TGG TCC GTG AAG CCT TCA GAT ATG TCC AAC AAA ACT TTC AAT CCC ATC CGA GCC ATT GTG GAC AGC ATG AAG GTG AAG CCA AAT CCA AAC AAA ACC ATG ATC GCT CTG TCA ATT GGG GAC	[240]
	W S V K P S D M S N K T F N P I R A I V D S M K V K P N P N K T M I A L S I G D	[80]
horse	TGG TCC GTG AGG CCC TCA GAC ATG TCC AAC AAA ACT TTC AAT CCC ATC CGG GCC ATC GTG GAC AAC ATG AAG GTG AAA CCA AAC CCA AAC AAA ACC ATG ATT GCT CTG TCA ATT GGG GAC	[240]
	W S V R P S D M S N K T F N P I R A I V D N M K V K P N P N K T M I A L S I G D	[80]
Cynopterus sphinx	TGG TCC GTG AAG CCC TCA GAC ATG GCC AAC AGA ACT TAC AAT CCC ATC CGG GCC ATA GTG GAC AGC ATG AAG ATG GAG CCA AAC CCA GAC AAA ACC ACG ATT ACC CTG TCC ATA GGG GAC	[240]
	W S V K P S D M A N R T Y N P I R A I V D S M K M E P N P D K T T I T L S I G D	[80]
Rousettus leschenaultii	TGG TCT GTG AGG CCC TCG GAC ATG GCC AAC AGA ACT TAC AAT CCC ATC CGG GCC ATA GTG GAC AAC ATG AAG TTG GAA CCA AAC CCA GAC AAA CCC ATG ATT TCC CTG TCC CTT GGG GAC	[240]
	W S V R P S D M A N R T Y N P I R A I V D N M K L E P N P D K P M I S L S L G D	[80]
Eonycteris spelaea	TGG TCT GTG AGG CCC TCG GAC ATG GCC AAC AGA ACT TAC AAT CCC ATC CGG GCC ATA GTG GAC AGC ATG AAG ATG GAA CCA AAC CCA GAC AAA ACC ACG ATT GCC CTG TCC ATT GGG GAC	[240]
	W S V R P S D M A N R T Y N P I R A I V D S M K M E P N P D K T T I A L S I G D	[80]
Pteropus vampyrus	TGG TCT GTG AGG CCC TCG GAC ATG GCC AAC AGA ACT TAC AAT CCC ATC CGG GCC ATA GTG GAC AGC ATG AAG ATG GAG CCC AAC CCA GAC AAA ACC ACG ATT CCC CTG TCC ATT GGG GAC	[240]
	W S V R P S D M A N R T Y N P I R A I V D S M K M E P N P D K T T I P L S I G D	[80]
Rhinolophus ferrumequinum	TGG TCT GTG AGG CCC TCG GAC ATG GCC AAC AAT ACT TTC AAT CCC ATC CGG ACC ATC GTG GAC AAC ATG AAG GTG AAG CCA AAC CCA CAC AAA ACC ATG ATT GCT CTG TCA ATT GGG GAC	[240]
	W S V R P S D M A N N T F N P I R T I V D N M K V K P N P H K T M I A L S I G D	[80]
Rhinolophus pusillus	TGG TCT GTG AAG CCT TCG GAC ATG TCC AAC AAA ACT TTC AAT CCC ATC CGG ACC ATC GTG GAC AAC ATG AAG GTG AAG CCA AAC CCA GAC AAA GCC ATG ATT GCT CTG TCA ATT GGG GAC	[240]
	W S V K P S D M S N K T F N P I R T I V D N M K V K P N P D K A M I A L S I G D	[80]
Hipposideros armiger	TGG TCC GTG AAG CCC TCG GAA ATG TCC AAC AAA ACT TTC AAT CCC ATC CGG ACC ATC GTA GAT GGC ATG AAG GTG AAG CCA AAC CCA AAC AAA ACC ATG ATT TCT CTG TCA ATC GGG GAC	[240]
	W S V K P S E M S N K T F N P I R T I V D G M K V K P N P N K T M I S L S I G D	[80]
Hipposideros pratti	TGG TCC GTG AAG CCC TCG GAA ATG TCC AAC AAA ACT TTC AAT CCC ATC CGG ACC ATC GTA GAT GGC ATG AAG GTG AAG CCA AAC CCA AAC AAA ACC ATG ATT TCT CTG TCA ATC GGG GAC	[240]
	W S V K P S E M S N K T F N P I R T I V D G M K V K P N P N K T M I S L S I G D	[80]
Megaderma lyra	TGG TCC GTG AAG CCC TCG GAC ATG TCC AAC AAA ACC TTC AAT CCC ATC CGG ACC ATC GTG GAC AGC ATG AAG GTG AAG CCA AAC CCA GAT AAA GCC ATG ATT GCT CTG TCA ATT GGG GAC	[240]
	W S V K P S D M S N K T F N P I R T I V D S M K V K P N P D K A M I A L S I G D	[80]
Megaderma spasma	TGG TCC GTG AAG CCC TCG GAC ATG TCC AAC AAC ACT TTC AAT CCC ATC CGG ACC ATC GTA GAC AGC ATG AAG GTG AAG CCA AAC CCA GAT AAA GCC ATG ATT GCT CTG TCA ATC GGG GAC	[240]
	W S V K P S D M S N N T F N P I R T I V D S M K V K P N P D K A M I A L S I G D	[80]
Myotis ricketti	TGG TCT GTG AAG CCC TCG GAC ATG TCC AAC AAC ACT TTC AAT CCC ATA CGG GCC ATC GTG GAC AGC ATG AAG GTG AAG CCA AAC CCG GAC AAA ACC ATG ATT TCT CTG TCA ATT GGG GAC	[240]
	W S V K P S D M S N N T F N P I R A I V D S M K V K P N P D K T M I S L S I G D	[80]
Pipistrellus abramus	TGG TCG GTG AAG CCC TCG GAC ATG GCC AAC AAC ACT TTC AAC CCC ATC CGG GCC ATC GTG GAC AGC ATG AAG GTG AAG CCG AAC CCA GAC AAA ACC ATG ATT TCT CTG TCC ATC GGC GAC	[240]
	W S V K P S D M A N N T F N P I R A I V D S M K V K P N P D K T M I S L S I G D	[80]
Scotophilus kuhlii	TGG TCT GTG AAG CCC TCG GAC ATG TCC AAC AAC ACT TTC AAT CCC ATC CGG GCC ATC GTG GAC AGC ATG AAG GTG AAG CCA AAC CCG GAC AAA GCC ATG ATT TCT CTG TCA ATT GGG GAC	[240]
	W S V K P S D M S N N T F N P I R A I V D S M K V K P N P D K A M I S L S I G D	[80]
Miniopterus fuliginosus	TGG TCC GTG AAG CCC TCG GAC ATG TCC AAC AAA ACT TTC AAT CCC ATC CGG GCC ATC GTG GAA AGC ATG AAG GTG AAG CCA AAC CCA GAC AAA ACC ATG ATT TCT CTG TCA ATT GGG GAC	[240]
	W S V K P S D M S N K T F N P I R A I V E S M K V K P N P D K T M I S L S I G D	[80]
Tadarida plicata	TGG TCC GTG AAG CCC TCG GAA ATG TCC AAG AAC ACT TTC AAT CCC ATC CGG GCC ATT GTG GAC AGC ATG AAG GTG AAG CCA AAC CCA GAC AAA ACC ATG ATT TCT CTG TCA ATT GGG GAC	[240]
	W S V K P S E M S K N T F N P I R A I V D S M K V K P N P D K T M I S L S I G D	[80]
Mormoops megalophylla	TGG TCT GTG AAG CCC TCG GAC ATG TCC AAC AAT ACT TTC AAT CCC ATC CGG GCC ATT GTG GAC AGC ATG AAG GTG AAG CCA AAT CCA GAC AAA CCC ATG ATT TCT CTG TCA ATT GGG GAC	[240]
	W S V K P S D M S N N T F N P I R A I V D S M K V K P N P D K P M I S L S I G D	[80]
Pteronotus parnellii	TGG TCT GTG AAG CCC TCG GAC ATG TCC AAC AAT ACA TTT AAT CCC ATC CGG GCC ATT GTG GAC AGC ATG AAG GTG AAG CCA AAC CCA GAC AAA ACC ATG ATC TCT CTG TCA ATT GGG GAC	[240]
	W S V K P S D M S N N T F N P I R A I V D S M K V K P N P D K T M I S L S I G D	[80]
Desmodus rotundus	TGG TCC GTG AAG CCC TCG GAC ATG GCC AAC AAC ACC TTC AAT CCT ATC CGT GAC ATT GTG GAC GGC ATG AAG GTG AAG CCA AAC CCA GCC AAA GCC ATG ATT TCT CTG TCA ATT GGG GAC	[240]
	W S V K P S D M A N N T F N P I R D I V D G M K V K P N P A K A M I S L S I G D	[80]
Leptonycteris yerbabuena	TGG TCC GTG AAG CCC TCA GAC ATG GCC AAC AAC ACT TTC AAT CCC ATC CGC GCC ATT GTG GAC AAC ATG AAG GTG AAG CCA AAC CCA GAC AAA ACC ATG ATT TCT CTG TCA ATT GGG GAC	[240]
	W S V K P S D M A N N T F N P I R A I V D N M K V K P N P D K T M I S L S I G D	[80]
Artibeus lituratus	TGG TCC GTG AAA CCT TCG GCC ATG GCC AAC AAC ACT TTC AAT CCC ATC CGT GCC ATT GTG GAC AGC ATG AAG GTG AAG CCA AAC CCA GAC AAA ACC ATG ATT TCT CTG TCA ATT GGG GAC	[240]
	W S V K P S A M A N N T F N P I R A I V D S M K V K P N P D K T M I S L S I G D	[80]

```
                                  V83L                            V93I T94I      M97L
human                       CCT ACT GTG TTT GGA AAC CTG CCT ACA GAC CCT GAA GTT ACC CAG GCA ATG AAA GAT GCC CTG GAC TCG GGC AAA TAT AAT GGC TAT GCC CCA TCC ATC GGC TTC CTA TCC AGT CGG GAG [360]
                             P   T   V   F   G   N   L   P   T   D   P   E   V   T   Q   A   M   K   D   A   L   D   S   G   K   Y   N   G   Y   A   P   S   I   G   F   L   S   S   R   E  [120]
mouse                       CCT ACT GTG TTT GGG AAC CTG CCC ACA GAC CCT GAA GTG ACC CAA GCC ATG AAG GAT GCC CTG GAC TCC GGG AAG TAC AAT GGC TAT GCC CCA TCT ATC GGC TAC CTA TCC AGT CGG GAG [360]
                             P   T   V   F   G   N   L   P   T   D   P   E   V   T   Q   A   M   K   D   A   L   D   S   G   K   Y   N   G   Y   A   P   S   I   G   Y   L   S   S   R   E  [120]
rat                         CCT ACT GTG TTT GGG AAC CTG CCT ACA GAC CCT GAA GTT ACC CAA GCC ATG AAA GAT GCC CTG GAC TCG GGG AAG TAC AAT GGC TAT GCC CCG TCC ATC GGC TAC CTA TCC AGT CGG GAG [360]
                             P   T   V   F   G   N   L   P   T   D   P   E   V   T   Q   A   M   K   D   A   L   D   S   G   K   Y   N   G   Y   A   P   S   I   G   Y   L   S   S   R   E  [120]
cow                         CCT ACT GTA TTT GGA AAC CTG CCG ACA GAT CCT GAA GTT ACC CAA GCA ATG AAA GAT GCC CTC GAC TCA GGG AAG TTT AAT GGC TAT GTC CCC TCC ATT GGC TAC TTA TCC AGT CGG GAG [360]
                             P   T   V   F   G   N   L   P   T   D   P   E   V   T   Q   A   M   K   D   A   L   D   S   G   K   F   N   G   Y   V   P   S   I   G   Y   L   S   S   R   E  [120]
dog                         CCT ACT GTG TTC GGA AAC CTG CCT ACA GAC CCT GAA GTT ACA CAG GCA ATG AAA GAT GCC CTG GAC TCG GGG AAG TAT AAC GGC TAT GCC CCC TCC ATT GGC TAC TTA TCC AGT CGG GAG [360]
                             P   T   V   F   G   N   L   P   T   D   P   E   V   T   Q   A   M   K   D   A   L   D   S   G   K   Y   N   G   Y   A   P   S   I   G   Y   L   S   S   R   E  [120]
pig                         CCT ACT GTG TTT GGA AAC CTG CCA ACA GAC CAG GAA GTA ACT CAA GCA ATG AAA GAT GCC CTG GAC TCG GGG AAG TAT AAC GGC TAT GCC CCC TCC ATT GGC TAC CTG TCC AGT CGG GAG [360]
                             P   T   V   F   G   N   L   P   T   D   Q   E   V   T   Q   A   M   K   D   A   L   D   S   G   K   Y   N   G   Y   A   P   S   I   G   Y   L   S   S   R   E  [120]
panda                       CCT ACT GTG TTT GGA AAC CTG CCT ACA GAC CCT GAA GTT ACC CAA GCC ATG AAA GAT GCC CTG GAC TCT GGG AAG TAT AAC GGC TAT GCC CCC TCC ATT GGC TAT TTA TCC AGT AGG GAG [360]
                             P   T   V   F   G   N   L   P   T   D   P   E   V   T   Q   A   M   K   D   A   L   D   S   G   K   Y   N   G   Y   A   P   S   I   G   Y   L   S   S   R   E  [120]
horse                       CCT ACT GTG TTT GGA AAC CTG TCT ACA GAT CCT GAA GTT ACC CAA GCA CTG AAA GAT GCC GTG GAC TCG GGG AAG TAT AAT GGC TAT GCC CCC ACC ATT GGC TAC CTA TCC AGT CGG GAG [360]
                             P   T   V   F   G   N   L   S   T   D   P   E   V   T   Q   A   L   K   D   A   V   D   S   G   K   Y   N   G   Y   A   P   T   I   G   Y   L   S   S   R   E  [120]
Cynopterus sphinx           CCT ACT CTG TCT GGA AAC CTG CCT ACA GAC CCA GAA ATT ATC CAA GCA TTG AAA GAT GCC CTG GAC TCA GGG AAA TAT AAT GGC TAC GCC CCC AGT ATT GGT TAC CTA TCC AGT CGG GAA [360]
                             P   T   L   S   G   N   L   P   T   D   P   E   I   I   Q   A   L   K   D   A   L   D   S   G   K   Y   N   G   Y   A   P   S   I   G   Y   L   S   S   R   E  [120]
Rousettus leschenaultii     CCT ACT CTG TTT GGA AAT CTG CCT ACA GAC CCG GAA ATT ATT CAA GCA TTG AAA GAT GCC CTG GAC TCG GGG AAG TAT AAT GGC TAC GCC CCC AGT ATC GGT TAC CTA TCC AGT AGG GAA [360]
                             P   T   L   F   G   N   L   P   T   D   P   E   I   I   Q   A   L   K   D   A   L   D   S   G   K   Y   N   G   Y   A   P   S   I   G   Y   L   S   S   R   E  [120]
Eonycteris spelaea          CCT ACT CTG TTT GGA AAC CTG CCT ACA GAC CCA GAA ATT ATT CAA GCA TTG AAA GAT GCC CTG GAC TCG GGG AAG TAT AAT GGC TAT GCC CCC AGT ATT GGT TAC CTA TCC AGT CGG GAA [360]
                             P   T   L   F   G   N   L   P   T   D   P   E   I   I   Q   A   L   K   D   A   L   D   S   G   K   Y   N   G   Y   A   P   S   I   G   Y   L   S   S   R   E  [120]
Pteropus vampyrus           CCT ACT CTG TGT GGA AAC CTG CCT ACA GAC CCA GAA ATT ATC AAA GCA TTG AAA GAT GCC CTG GAC TCG GGG AAG TAT AAT GGC TAC GCC CCC AGT ATT GGT TAC CTA TCC AGT CGG GAA [360]
                             P   T   L   C   G   N   L   P   T   D   P   E   I   I   K   A   L   K   D   A   L   D   S   G   K   Y   N   G   Y   A   P   S   I   G   Y   L   S   S   R   E  [120]
Rhinolophus ferrumequinum   CCT ACT GTG TTT GGA AAC CTG CCT ACA GAC CCG GAA GTT ACC CAA GCA ATG AAA GAT GCC CTG GAC TCC GGA AAG TAT AAT GGC TAT GCT CCT TCC ATT GGT TAC CTA TCC AGT CGG GAA [360]
                             P   T   V   F   G   N   L   P   T   D   P   E   V   T   Q   A   M   K   D   A   L   D   S   G   K   Y   N   G   Y   A   P   S   I   G   Y   L   S   S   R   E  [120]
Rhinolophus pusillus        CCT ACT GTG TTT GGA AAC CTG CCT ACA GAC CCG GAA GTT ACC CAA GCA ATG AAA GAT GCT CTG GAC TCC GGA AAG TAT AAT GGC TAT GCT CCT TCC ATT GGT TAC CTA TCC AGT CGG GAA [360]
                             P   T   V   F   G   N   L   P   T   D   P   E   V   T   Q   A   M   K   D   A   L   D   S   G   K   Y   N   G   Y   A   P   S   I   G   Y   L   S   S   R   E  [120]
Hipposideros armiger        CCT ACT GTG TTT GGA AAT CTG CCT ACA GAC CCG GAA GTT ACC CAA GCA ATG AAA GAT GCC CTG GAC TCC GGG AAG TAT AAT GGC TAT GCT CCC TCC ATT GGT TAC CTA TCC AGT CGG GAA [360]
                             P   T   V   F   G   N   L   P   T   D   P   E   V   T   Q   A   M   K   D   A   L   D   S   G   K   Y   N   G   Y   A   P   S   I   G   Y   L   S   S   R   E  [120]
Hipposideros pratti         CCT ACT GTG TTT GGA AAT CTG CCT ACA GAC CCG GAA GTC ACC CAA GCA ATG AAA GAT GCC CTG GAC TCC GGG AAG TAT AAT GGC TAT GCT CCC TCC ATT GGT TAC CTA TCC AGT CGG GAA [360]
                             P   T   V   F   G   N   L   P   T   D   P   E   V   T   Q   A   M   K   D   A   L   D   S   G   K   Y   N   G   Y   A   P   S   I   G   Y   L   S   S   R   E  [120]
Megaderma lyra              CCT ACT GTG TTC GGG AAC CTG CCT ACA GAC CCG GAA GTT ACC CAA GCA ATG AAA GAT GCC CTG GAC TCG GGG AAG TAT AAC GGC TAT GCC CCC TCC ATT GGT TAC CTA TCC AGT CGG GAG [360]
                             P   T   V   F   G   N   L   P   T   D   P   E   V   T   Q   A   M   K   D   A   L   D   S   G   K   Y   N   G   Y   A   P   S   I   G   Y   L   S   S   R   E  [120]
Megaderma spasma            CCT ACT GTG TTT GGA AAC CTG CCT ACA GAC CCG GAA GTT ACC CAA GCA ATG AAA GAT GCC CTG GAC TCG GGG AAG TAT AAC GGC TAT GCC CCC TCC ATT GGT TAC CTA TCC AGT CGG GAA [360]
                             P   T   V   F   G   N   L   P   T   D   P   E   V   T   Q   A   M   K   D   A   L   D   S   G   K   Y   N   G   Y   A   P   S   I   G   Y   L   S   S   R   E  [120]
Myotis ricketti             CCT ACT GTG TTT GGA AAC CTG CCC ACG GAC CCA GAA GTT ACC CAG GCG ATG AAA GAT GCC CTG GAC TCC GGG AAG TAT AAC GGC TAC GCC CCC TCC ATT GGT TAC CTG TCC AGC CGG GAA [360]
                             P   T   V   F   G   N   L   P   T   D   P   E   V   T   Q   A   M   K   D   A   L   D   S   G   K   Y   N   G   Y   A   P   S   I   G   Y   L   S   S   R   E  [120]
Pipistrellus abramus        CCT ACT GTG TTT GGA AAC CTG CCC ACG GAC CCA GAA GTT ACC CAG GCG ATG AAG GAT GCC CTG GAC TCC GGG AAG TAT AAC GGC TAT GCC CCC TCC ATT GGT TTC CTG TCC AGC CGG GAA [360]
                             P   T   V   F   G   N   L   P   T   D   P   E   V   T   Q   A   M   K   D   A   L   D   S   G   K   Y   N   G   Y   A   P   S   I   G   F   L   S   S   R   E  [120]
Scotophilus kuhlii          CCG ACT GTG TTT GGA AAC CTG CCC ACG GAC CCA GAG GTT ACG CAG GCG ATG AAA GAT GCC CTG GAC TCC GGG AAG TAT AAC GGC TAC GCC CCC TCC ATT GGT TAT CTA TCC AGC CGG GAA [360]
                             P   T   V   F   G   N   L   P   T   D   P   E   V   T   Q   A   M   K   D   A   L   D   S   G   K   Y   N   G   Y   A   P   S   I   G   Y   L   S   S   R   E  [120]
Miniopterus fuliginosus     CCT ACT GTG TTT GGA AAC CTG CCT ACA GAC CCA GAA GTT ACC CAG GCA ATG AAG GAT GCC CTG GAC TCG GGG AAG TAT AAC GGC TAC GCC CCC TCC ATT GGT TAC CTG TCC AGT CGG GAG [360]
                             P   T   V   F   G   N   L   P   T   D   P   E   V   T   Q   A   M   K   D   A   L   D   S   G   K   Y   N   G   Y   A   P   S   I   G   Y   L   S   S   R   E  [120]
Tadarida plicata            CCT ACT GTG TTT GGA AAC CTG CCC ACA GAC CCG GAA GTT ATC CAG GCA ATG AAA GAT GCC CTG GAC TCG GGG AAG TAC AAC GGC TAT GCC CCC TCC ATT GGT TAC CTA TCC AGC CGG GAA [360]
                             P   T   V   F   G   N   L   P   T   D   P   E   V   I   Q   A   M   K   D   A   L   D   S   G   K   Y   N   G   Y   A   P   S   I   G   Y   L   S   S   R   E  [120]
Mormoops megalophylla       CCT ACT GTG TTT GGA AAC TTG CCT ACA GAC CCT GAA GTT ACC CAA GCA ATG AAA GAT GCT CTG GAC TCG GGG AAG TAT AAT GGC TAT GCC CCC TCC ATT GGT TAC CTG TCC AGC CGG GAA [360]
                             P   T   V   F   G   N   L   P   T   D   P   E   V   T   Q   A   M   K   D   A   L   D   S   G   K   Y   N   G   Y   A   P   S   I   G   Y   L   S   S   R   E  [120]
Pteronotus parnellii        CCT ACT GTG TTT GGA AAC TTG CCT ACA GAC CCT GAA GTT ACC CAA GCA ATG AAA GAT GCT CTG GAC TCG GGG AAG TAT AAC GGC TAT GCC CCC TCC ATT GGT TAC CTA TCC AGT CGG GAA [360]
                             P   T   V   F   G   N   L   P   T   D   P   E   V   T   Q   A   M   K   D   A   L   D   S   G   K   Y   N   G   Y   A   P   S   I   G   Y   L   S   S   R   E  [120]
Desmodus rotundus           CCT ACG GTG TTT GGA AAC CTG CCT ACA GAC CCT GAA GTT ATC CAA GCA ATG AAG GAT GCC CTG GAC TCG GGA AAG TAT AAT GGC TAT GCC CCC TCC ATT GGT TAC CTG TCC AGT CGG GAA [360]
                             P   T   V   F   G   N   L   P   T   D   P   E   V   I   Q   A   M   K   D   A   L   D   S   G   K   Y   N   G   Y   A   P   S   I   G   Y   L   S   S   R   E  [120]
Leptonycteris yerbabuena    CCT ACA GTG TTT GGA AAC CTG CCT ACA GAC CCT GAA GTT ACC CAA GCA ATG AAG GAT GCC CTG GAC TCA GGG AAG TAT AAC GGC TAT GCC CCC TCC ATC GGT TAC CTG TGC AGT CGG GAA [360]
                             P   T   V   F   G   N   L   P   T   D   P   E   V   T   Q   A   M   K   D   A   L   D   S   G   K   Y   N   G   Y   A   P   S   I   G   Y   L   C   S   R   E  [120]
Artibeus lituratus          CCT ACA GTG TTT GGA AAC CTG CCT ACA GAC CCT GAA GTT ACC CAA GCA ATG AAG GAT GCC CTG GAC TCG GGG AAG TAT AAC GGC TAT GCC CCC TCC ATC GGT TAC CTG TCT AGT CGG GAG [360]
                             P   T   V   F   G   N   L   P   T   D   P   E   V   T   Q   A   M   K   D   A   L   D   S   G   K   Y   N   G   Y   A   P   S   I   G   Y   L   S   S   R   E  [120]
```

human	GAG ATT GCT TCT TAT TAC CAC TGT CCT GAG GCA CCC CTA GAA GCT AAG GAC GTC ATT CTG ACA AGT GGC TGC AGC CAA GCT ATT GAC CTT TGT TTA GCT GTG TTG GCC AAC CCA GGG CAA	[480]
	E I A S Y Y H C P E A P L E A K D V I L T S G C S Q A I D L C L A V L A N P G Q	[160]
mouse	GAG GTC GCT TCC TAT TAC CAC TGT CCT GAG GCT CCT CTG GAA GCT AAG GAT GTC ATT CTG ACG AGT GGC TGC AGT CAG GCC ATT GAG CTG TGT CTA GCC GTG TTG GCC AAT CCT GGA CAG	[480]
	E V A S Y Y H C P E A P L E A K D V I L T S G C S Q A I E L C L A V L A N P G Q	[160]
rat	GAG GTC GCT TCT TAC TAC CAC TGT CAT GAG GCT CCT CTG GAA GCT AAG GAT GTC ATT CTG ACA AGC GGC TGC AGT CAG GCC ATT GAG CTA TGT CTA GCT GTG TTG GCC AAT CCT GGA CAA	[480]
	E V A S Y Y H C H E A P L E A K D V I L T S G C S Q A I E L C L A V L A N P G Q	[160]
cow	GAA GTT GCT TCT TAT TAC CAC TGT CCC GAG GCA CCC CTG GAA GCT AAG GAT GTC ATT CTG ACA AGT GGC TGC AGT CAG GCT ATT GAA CTG TGT TTG GCT GTG TTG GCC AAC CCA GGG CAA	[480]
	E V A S Y Y H C P E A P L E A K D V I L T S G C S Q A I E L C L A V L A N P G Q	[160]
dog	GAG ATT GCT TCG TAT TAC CAC CGG CCC GAG GCA CCC CTG GAA GCT AAG GAT GTC ATT CTG ACC AGT GGC TGC AGT CAG GCT ATT GAA CTT TGT TTA GCT GTA TTG GCT AAT CCA GGA CAA	[480]
	E I A S Y Y H R P E A P L E A K D V I L T S G C S Q A I E L C L A V L A N P G Q	[160]
pig	GAG GTT GCT TCT TAT TAC CAC TGT CCG GAG GCA CCC CTG GAA GCT AAG GAT GTC ATT CTG ACA AGC GGC TGC AGT CAG GCT ATT GAA CTT TGT TTA GCT GTG TTG GCT AAC CCG GGC CAA	[480]
	E V A S Y Y H C P E A P L E A K D V I L T S G C S Q A I E L C L A V L A N P G Q	[160]
panda	GAG ATT GCT TCT TAC TAC CAC CGG CCT GAG GCT CCC CTG GAA GCT AAG GAT GTC ATT CTG ACT AGT GGC TGC AGT CAG GCT ATT GAA CTT TGT TTA GCT GTG TTG GCC AAC CCA GGA CAA	[480]
	E I A S Y Y H R P E A P L E A K D V I L T S G C S Q A I E L C L A V L A N P G Q	[160]
horse	GAG ATC GCT TCT TAT TAC CAT CAT CCC GAG GCA CCT CTG GAA GCT AAG GAT GTC ATT CTG ACA AGT GGC TGC AGT CAG GCT ATT GAA CTT TGT TTA GCT GTG TTG GCC AAC CCA GGG CAA	[480]
	E I A S Y Y H H P E A P L E A K D V I L T S G C S Q A I E L C L A V L A N P G Q	[160]
Cynopterus sphinx	GAG GTT GCT TCT TAT TTC CGC TGT CCC GAG GCC CCC CTG GAA GCT AAG GAT GTC ATT CTG ACA AGT GGC TGC AGT CAG GCT GTT GAA CTT TGT TTA GCT GTG TTG GCC AAC CCA GGG CAA	[480]
	E V A S Y F R C P E A P L E A K D V I L T S G C S Q A V E L C L A V L A N P G Q	[160]
Rousettus leschenaultii	GAG ATT GCT TCT TAT TAC CAC CAT CCC AAG GCA CCC CTG GAA GCT AAG GAC GTC ATT CTG ACA TGT GGC TGC AGT CAG GCT ATT GAA CTT TGT TTA GCT GTG TTG GCC AAC CCA GGG CAA	[480]
	E I A S Y Y H H P K A P L E A K D V I L T C G C S Q A I E L C L A V L A N P G Q	[160]
Eonycteris spelaea	GAG GTT GCT TCT TAT TAC CAC CAT CCC AAG GCA CCC CTG GAA GCT AAG GAT GTC ATT CTG ACA TGT GGC TGC AGT CAG GCT ATT GAA CTT TGT TTA ACT GTG TTG GCC AAC CCA GGG CAA	[480]
	E V A S Y Y H H P K A P L E A K D V I L T C G C S Q A I E L C L T V L A N P G Q	[160]
Pteropus vampyrus	GAG ATT GCT TCT TAT TAC CAC CGT CCC GAG GCA CCT CTG GAA GCT AAG GAT GTT ATT CTG ACA AGT GGC TGC AGT CAG GCT ATT GAA CTT TGT TTA GCT GTG TTG GCC AAC CCA GGG CAA	[480]
	E I A S Y Y H R P E A P L E A K D V I L T S G C S Q A I E L C L A V L A N P G Q	[160]
Rhinolophus ferrumequinum	GAG ATT GCT TCT TAT TAC CAC TGT CCT GAG GCA CCC CTG GAG GCT AAT GAT GTC ATT CTG ACA AGT GGC TGC GGT CAG GCT ATT GAA CTT TGT TTA GCT GTG TTG GCC AAC CCA GGG CAA	[480]
	E I A S Y Y H C P E A P L E A N D V I L T S G C G Q A I E L C L A V L A N P G Q	[160]
Rhinolophus pusillus	GAG ATT GCT TCT TAT TAC CAC TGT CCT GAG GCA CCC CTG GAG GCC AAG GAT GTC ATT CTG ACA AGT GGC TGC AGT CAG GCT ATT GAA CTT TGT TTA GCT GTG TTG GCC AAC CCA GGG CAA	[480]
	E I A S Y Y H C P E A P L E A K D V I L T S G C S Q A I E L C L A V L A N P G Q	[160]
Hipposideros armiger	GAG GTT GCT TCT TAT TAC CAC TGT CCC GAG GCA CCC CTG GAA GCT AAG GAT GTC ATT CTG ACA AGT GGC TGC AGT CAG GCT ATT GAA CTT TGC TTA GCT GTG TTG GCC AAC CCA GGG CAA	[480]
	E V A S Y Y H C P E A P L E A K D V I L T S G C S Q A I E L C L A V L A N P G Q	[160]
Hipposideros pratti	GAG GTT GCT TCT TAT TAC CAC TGT CCC GAG GCA CCC CTG GAA GCT AAG GAT GTC ATT CTG ACA AGT GGC TGC AGT CAG GCT ATT GAA CTT TGC TTA GCT GTG TTG GCT AAC CCA GGG CAA	[480]
	E V A S Y Y H C P E A P L E A K D V I L T S G C S Q A I E L C L A V L A N P G Q	[160]
Megaderma lyra	GAG ATT GCT TCT TAT TAC CAC TGT CCT GAG GCA CCC CTG GAA GCT AAG GAT ATC ATT CTG ACA AGT GGC TGC AGT CAA GCT ATT GAA CTT TGT CTA GCT GTG TTG GCC AAC CCA GGG CAA	[480]
	E I A S Y Y H C P E A P L E A K D I I L T S G C S Q A I E L C L A V L A N P G Q	[160]
Megaderma spasma	GAG ATT GCT TCT TAT TAC CAC TGT CCT GAG GCA CCC CTG GAA GCA AAG GAT GTC ATT CTG ACA AGC GGC TGC AGT CAG GCT ATT GAA CTT TGT TTA GCT GTG TTG GCC AAC CCA GGG CAA	[480]
	E I A S Y Y H C P E A P L E A K D V I L T S G C S Q A I E L C L A V L A N P G Q	[160]
Myotis ricketti	GAG GTC GCG TCC TAT TAC CAC TGT CCC GAG GCA CCC CTG GAA GCT AAG GAT GTC ATT CTG ACA AGT GGC TGC AGT CAG GCT ATT GAA CTT TGT TTA GCT GTG TTG GCC AAC CCA GGG CAA	[480]
	E V A S Y Y H C P E A P L E A K D V I L T S G C S Q A I E L C L A V L A N P G Q	[160]
Pipistrellus abramus	GAG GTT GCC TCC TAT TAC CAC TGT CCC GAG GCA CCC CTG GAA GCT AAG GAT GTC ATT CTG ACA AGT GGC TGC AGT CAG GCT ATT GAA CTT TGC TTA GCT GTG TTG GCC AAC CCA GGG CAA	[480]
	E V A S Y Y H C P E A P L E A K D V I L T S G C S Q A I E L C L A V L A N P G Q	[160]
Scotophilus kuhlii	GAG GTT GCC TCC TAT TAC CAC TGT CCC GAG GCT CCC CTG GAA GCT GAG GAT GTC ATT CTG ACA AGT GGC TGC AGT CAG GCT ATT GAA CTT TGT TTA GCT GTG TTG GCC AAC CCA GGG CAA	[480]
	E V A S Y Y H C P E A P L E A E D V I L T S G C S Q A I E L C L A V L A N P G Q	[160]
Miniopterus fuliginosus	GAA ATT GCT TCT TAC TAC CAC TGC CCC GAG GCG CCC TTG GAA GCT AAG GAT GTC ATT CTG ACA AGT GGC TGC AGT CAG GCT ATT GAA CTG TGT TTA GCT GTG TTG GCC AAC CCA GGG CAA	[480]
	E I A S Y Y H C P E A P L E A K D V I L T S G C S Q A I E L C L A V L A N P G Q	[160]
Tadarida plicata	GAG ATT GCC TCT TAC TAC CAC TGT CCC GAG GCA CCC CTG GAA GCT AAG GAT GTC ATT CTG ACA AGT GGG TGC AGT CAG GCT ATT GAA CTT TGT TTA GCT GTG TTG GCC AAC CCA GGG CAA	[480]
	E I A S Y Y H C P E A P L E A K D V I L T S G C S Q A I E L C L A V L A N P G Q	[160]
Mormoops megalophylla	GAG ATT GCT TCT TAT TAC CAC TGT CCC GAG GCA CCC CTG GAA GCT AAG GAT GTC ATT CTA ACA AGT GGC TGC AGT CAG GCT ATT GAA CTT TGT TTA GCA GTG CTG GCC AAC CCA GGG CAG	[480]
	E I A S Y Y H C P E A P L E A K D V I L T S G C S Q A I E L C L A V L A N P G Q	[160]
Pteronotus parnellii	GAG ATT GCT TCT TTT TAC CAC TGT CCC GAG GCA CCC CTG GAA GCT AAG GAT GTC ATT CTG ACC AGT GGC TGC AGT CAG GCT ATT GAA CTT TCT TTA GCA GTG TTG GCC AAC CCA GGG CAG	[480]
	E I A S F Y H C P E A P L E A K D V I L T S G C S Q A I E L S L A V L A N P G Q	[160]
Desmodus rotundus	GAG ATT GTT TCT TAT CAC CAC TGT CCC GAG GCA CCC CTG GAA GCT AAG GAT GTC ATT CTG ACA AGT GGC TGC AGT CAG GCC ATT GAA CTT TGT TTA GCA GTG CTG GCC AAT CCG GGG CAG	[480]
	E I V S Y H H C P E A P L E A K D V I L T S G C S Q A I E L C L A V L A N P G Q	[160]
Leptonycteris yerbabuena	GAT ATT GCT TCT TAT TAC CAC TGT CCC GAG GCA CCC CTG GAA GCT AAG GAT GTC CTT CTG ACA AGT GGC TGC AGT CAG GCC ATT GAA CTT TGT TTA GCA GTG TTG GCC AAC CCA GGG CAG	[480]
	D I A S Y Y H C P E A P L E A K D V L L T S G C S Q A I E L C L A V L A N P G Q	[160]
Artibeus lituratus	GAG ATT GCT TCT TAT TAC CAC TGT CCC GAG GCA CCC CTG GAA GCT AAG GAT GTC ATT CTG ACA AGT GGC TGC AGT CAG GCC ATT GAA CTT TGT TTA GCA GTG CTG GCC AGC CCA GGG CAG	[480]
	E I A S Y Y H C P E A P L E A K D V I L T S G C S Q A I E L C L A V L A S P G Q	[160]

	V164A L171P	
human	AAC ATC CTG GTT CCA AGA CCT GGT TTC TCT CTC TAC AAG ACT CTG GCT GAG TCT ATG GGA ATT GAG GTC AAA CTC TAC AAT TTG TTG CCA GAG AAA TCT TGG GAA ATT GAC CTG AAA CAA	[600]
	N I L V P R P G F S L Y K T L A E S M G I E V K L Y N L L P E K S W E I D L K Q	[200]
mouse	AAC ATC CTC ATT CCG AGG CCC GGG TTT TCC CTC TAC AGG ACA TTG GCT GAG TCT ATG GGG ATT GAG GTC AAG CTC TAC AAT CTA TTG CCT GAG AAG TCT TGG GAA ATT GAT CTA AAA CAA	[600]
	N I L I P R P G F S L Y R T L A E S M G I E V K L Y N L L P E K S W E I D L K Q	[200]
rat	AAC ATC CTC ATT CCA AGG CCC GGG TTT TCC CTC TAT AGG ACT TTG GCT GAG TCT ATG GGA ATT GAG GTC AAG CTC TAC AAT CTC CTG CCC GAG AAG TCT TGG GAA ATT GAC CTA AAA CAA	[600]
	N I L I P R P G F S L Y R T L A E S M G I E V K L Y N L L P E K S W E I D L K Q	[200]
cow	AAC ATC CTA GTT CCG AGA CCT GGC TTC TCT CTC TAC AGG ACT CTG GCT GAA TCT ATG GGA ATT GAG GTC AAA CTC TAC AAT TTA TTG CCA GAG AAG AAT TGG GAA ATT GAC CTG AAA CAA	[600]
	N I L V P R P G F S L Y R T L A E S M G I E V K L Y N L L P E K N W E I D L K Q	[200]
dog	AAC ATC CTA GTT CCG AGA CCT GGT TTC TCT CTC TAC AGG ACT TTG GCT GAA TCG ATG GGA ATT GAG GTC AAA CTC TAC AAT TTA TTG CCA GAG AAG TCT TGG GAA ATT GAC TTG AAA CAA	[600]
	N I L V P R P G F S L Y R T L A E S M G I E V K L Y N L L P E K S W E I D L K Q	[200]
pig	AAC ATC CTG GTT CCG AGA CCC GGT TTC CCT CTC TAC AGG ACT CTG GCT GAA TCT ATG GGA ATC GAG GTC AAA CTC TAC AAT TTG TTG CCA GAG AAG TCT TGG GAA ATT GAC CTG AAA CAT	[600]
	N I L V P R P G F P L Y R T L A E S M G I E V K L Y N L L P E K S W E I D L K H	[200]
panda	AAC ATC CTA GTT CCG AGA CCC GGT TTC TCT CTC TAC AGG ACT TTG GCT GAA TCG ATG GGA ATT GAG GTC AAA CTC TAC AAT TTA TTG CCA GAG AAG TCT TGG GAA ATT GAC TTG AAA CAA	[600]
	N I L V P R P G F S L Y R T L A E S M G I E V K L Y N L L P E K S W E I D L K Q	[200]
horse	AAC ATC CTA GTT CCC AGA CCT GGC TTT GCT CTC TAC AGG ACT TTG GCT GAA TCC ATG GGA ATT GAG GTC AAA TGC TAC AAT TTA TTG CCA GAG AAG TCT TGG GAA ATT GAC CTG AAA CAA	[600]
	N I L V P R P G F A L Y R T L A E S M G I E V K C Y N L L P E K S W E I D L K Q	[200]
Cynopterus sphinx	AAC ATC CTA GCC CCG AGA CCT GGT TTC TCT CCC TAC AGG ACT TTG GCT GAA TCT ATG GGG ATT AAG ATC AAA CTC TAC AAT TTA TTG CCA GAG AAA TCT TGG GAA ATT GAC CTG AAA CAA	[600]
	N I L A P R P G F S P Y R T L A E S M G I K I K L Y N L L P E K S W E I D L K Q	[200]
Rousettus leschenaultii	AAC ATC CTA GCC CCG AGA CCC GGT TTC TCT CCC TAC AGG ACT CAT GCT GAA TCT ATG GGG ATT GAG CTC AAA CTC TAC AAT TTA TTG CCA GAG AAA TCT TGG GAA ATT GAC CTG AAA CAG	[600]
	N I L A P R P G F S P Y R T H A E S M G I E L K L Y N L L P E K S W E I D L K Q	[200]
Eonycteris spelaea	AAC ATC CTA GCC CCG AGA CCC GGC TTC TCT CCC TAC AGG ACT CTT GCT GAA TCT CTG GGG GTT GAG GTC AAA CTC TAC AAT TTA TTG CCA GAG AAA TAT TGG GAA ATT GAC CTG GAA CAA	[600]
	N I L A P R P G F S P Y R T L A E S L G V E V K L Y N L L P E K Y W E I D L E Q	[200]
Pteropus vampyrus	AAC ATC CTA GCC CCG AGA CCC GGT TTC TCT CCC TAC AGG ACT TTG GCT GAA TCA TTG GGA ATT GAG ATC AAA CTC TAC AAT TTA TTG CCA GAG AAA TCT TGG GAA GTT GAC CTG AAA CAA	[600]
	N I L A P R P G F S P Y R T L A E S L G I E I K L Y N L L P E K S W E V D L K Q	[200]
Rhinolophus ferrumequinum	AAC ATC CTA CTT CCA AGA CCC GGC TTT TCT CTA TAT AGG ACT TTG GCT GAA TCT ATG GGG ATT GAG GTT AAA CTC TAC AAT TTA TTG CCA GAG AAG TCT TGG GAA ATT GAC CTG AAA CAA	[600]
	N I L L P R P G F S L Y R T L A E S M G I E V K L Y N L L P E K S W E I D L K Q	[200]
Rhinolophus pusillus	AAC ATC CTA ATT CCA AGA CCC GGC TTT TCT CTA TAC AGG ACT TTG GCT GAA TCT ATG GGG ATT GAG GTC AAA CTC TAC AAT TTA TTG CCA GAG AAG TCT TGG GAA ATT GAC CTG AAA CAA	[600]
	N I L I P R P G F S L Y R T L A E S M G I E V K L Y N L L P E K S W E I D L K Q	[200]
Hipposideros armiger	AAC ATC CTG GTT CCG AGA CCT GGT TTT TCT CTG TAT AGG ACT TTG GCT GAA TCT ATG GGG ATT GAG GTC AAA CTC TAC AAT TTA TTG CCA GAG AAG TCT TGG AAA ATT GAC CTG AAA CAA	[600]
	N I L V P R P G F S L Y R T L A E S M G I E V K L Y N L L P E K S W K I D L K Q	[200]
Hipposideros pratti	AAC ATC CTG GTT CCG AGA CCT GGT TTT TCT CTG TAT AGG ACT TTG GCT GAA TCT ATG GGG ATT GAG GTC AAA CTC TAC AAT TTA TTG CCA GAG AAG TCT TGG AAA ATT GAC CTG AAA CAA	[600]
	N I L V P R P G F S L Y R T L A E S M G I E V K L Y N L L P E K S W K I D L K Q	[200]
Megaderma lyra	AAC ATC CTG GTT CCG AGA CCT GGT TTC TCT CTC TAT AAG ACT TTG GCT GAA TCT ATG GGG ATT GAG GTT AAA CTC TAC AAT TTA TTG CCA GAG AAG TCT TGG GAG ATT GAT CTG AAA CAA	[600]
	N I L V P R P G F S L Y K T L A E S M G I E V K L Y N L L P E K S W E I D L K Q	[200]
Megaderma spasma	AAC ATC CTA GTT CCA AGG CCT GGT TTC TCT CTC TAT AAG ACT TTG GCT GAA TCT ATG GGG ATT GAG GTT AAA CTC TAC AAT TTA TTG CCA GAG AAG TCT TGG GAG ATT GAC CTG AAA CAA	[600]
	N I L V P R P G F S L Y K T L A E S M G I E V K L Y N L L P E K S W E I D L K Q	[200]
Myotis ricketti	AAC ATC CTC GTT CCC AGA CCC GGC TTC TCT CTC TAC AGG ACT TTG GCT GAA TCT ATG GGG ATT GAG ATC AAA CTC TAC AAC TTA TTG CCA GAG AAG TCT TGG GAA ATT GAC CTG AAA CAA	[600]
	N I L V P R P G F S L Y R T L A E S M G I E I K L Y N L L P E K S W E I D L K Q	[200]
Pipistrellus abramus	AAC ATC CTC GTT CCG AGA CCC GGC TTC TCT CTC TAC AGG ACT TTG GCT GAA TCT ATG GGG ATT GAG GTC AAA CTC TAC AAC TTA TTG CCA GAG AAG TCT TGG GAA ATT GAC CTG AAA CAA	[600]
	N I L V P R P G F S L Y R T L A E S M G I E V K L Y N L L P E K S W E I D L K Q	[200]
Scotophilus kuhlii	AAC ATC CTT GTT CCG AGA CCC GGT TTC TCT CTC TAC AGG ACT TTG GCT GAA TCT ATG GGG ATT GAG GTC AAA CTC TAC AAC TTA TTG CCA GAG AAG TCT TGG GAA ATT GAC CTG AAA CAG	[600]
	N I L V P R P G F S L Y R T L A E S M G I E V K L Y N L L P E K S W E I D L K Q	[200]
Miniopterus fuliginosus	AAC ATC CTA GTT CCG AGA CCC GGT TTC TCT CTC TAC AGG ACT TTG GCT GAA TCT ATG GGG ATT GAG ATC AAG CTC TAC AAT TTA TTG CCA GAG AAG TCT TGG GAA GCT GAC CTG AAA CAA	[600]
	N I L V P R P G F S L Y R T L A E S M G I E I K L Y N L L P E K S W E A D L K Q	[200]
Tadarida plicata	AAC ATC CTG GTT CCA AGA CCA GGT TTC TCT CTC TAC AGG ACT TTG GCT GAA TCT ATG GGG ATT GAG ATC AAA CTC TAC AAT TTA TTG CCA GAG AAG TCG TGG GAA ATT GAC CTG AAA CAA	[600]
	N I L V P R P G F S L Y R T L A E S M G I E I K L Y N L L P E K S W E I D L K Q	[200]
Mormoops megalophylla	AAC ATC CTA GTT CCG AGA CCC GGC TTC TCT CTC TAC AGG ACT TTG GCT GAA TCT CTG GGG GTT GAA GTC AGA TTC TAC AAT TTA TTG CCA GAG AAA TCT TGG GAA ATT GAC CTG AAA CAA	[600]
	N I L V P R P G F S L Y R T L A E S L G V E V R F Y N L L P E K S W E I D L K Q	[200]
Pteronotus parnellii	AAC ATC CTC GTG CCG AGG CCC GGT TTT TCT CTC TAC AGG ACT TTG GCT GAA TCT ATG GGG ATA GAG GTC AAA CTC TAC AAT TTA TTG CCA GAG AAG TCT TGG GAA GTT GAC CTG AAA CAA	[600]
	N I L V P R P G F S L Y R T L A E S M G I E V K L Y N L L P E K S W E V D L K Q	[200]
Desmodus rotundus	AAC ATC CTG GTT CCT AGA CCC GGT TTC TCT CTC TAC AGG ACT TTG ACT GAA TCT ATG GGG ATT GAG ATC AAA CTC TAC AAT TTA TTG CCA GAG AAG TCT TGG GAA ATT GAC CTG AAA CAG	[600]
	N I L V P R P G F S L Y R T L T E S M G I E I K L Y N L L P E K S W E I D L K Q	[200]
Leptonycteris yerbabuena	AAC ATC CTG GTT CCG AGA CCC GGT TTC TCT CTC TAT AGG ACT TTG GCT GAA TCT ATG GGG ATT GAG GTC AAA CTC TAC AAT TTA TTG CCA GAG AAG TCT TGG GAA ATT GAC CTG AAA CAA	[600]
	N I L V P R P G F S L Y R T L A E S M G I E V K L Y N L L P E K S W E I D L K Q	[200]
Artibeus lituratus	AAC ATC CTG GTT CCC AGA CCC GGT TTC TCT CTC TAT AGG ACT TTG GCT GAA TCT ATG GGG ATC GAG GTC AAA CTC TAC AAT TTA TTG CCA GAG AAG TCT TGG GAA ATT GAC CTG AAA CAA	[600]
	N I L V P R P G F S L Y R T L A E S M G I E V K L Y N L L P E K S W E I D L K Q	[200]

K232D

human	CTG GAA TAT CTA ATT GAT GAA AAG ACA GCT TGT CTC ATT GTC AAT AAT CCA TCA AAC CCC TGT GGG TCA GTG TTC AGC AAA CGT CAT CTT CAG AAG ATT CTG GCA GTG GCT GCA CGG CAG	[720]
	L E Y L I D E K T A C L I V N N P S N P C G S V F S K R H L Q K I L A V A A R Q	[240]
mouse	CTG GAA TCT CTG ATC GAC GAA AAA ACA GCT TGT CTC GTG GTC AAC AAC CCG TCC AAT CCC TGT GGC TCT GTG TTC AGT AAG CGG CAC CTT CAG AAG ATT TTG GCA GTG GCT GAA AGG CAA	[720]
	L E S L I D E K T A C L V V N N P S N P C G S V F S K R H L Q K I L A V A E R Q	[240]
rat	CTG GAA TCT CTG ATC GAT GAA AAA ACA GCG TGT CTT GTT GTC AAC AAC CCA TCC AAT CCC TGT GGC TCC GTG TTC AGT AAG CGG CAC CTT CAG AAG ATT TTG GCA GTG GCT GAA AGG CAG	[720]
	L E S L I D E K T A C L V V N N P S N P C G S V F S K R H L Q K I L A V A E R Q	[240]
cow	TTA GAA TCT CTG ATT GAT GAA AAG ACA GTT TGT CTT ATT GTC AAC AAT CCA TCA AAC CCT TGT GGG TCA GTG TTC AGT AGA CGT CAT CTC CAG AAA ATT TTG GCA GTG GCT GCA AGG CAA	[720]
	L E S L I D E K T V C L I V N N P S N P C G S V F S R R H L Q K I L A V A A R Q	[240]
dog	CTG GAA TCT CTG ATT GAT GAA AAG ACA GCT TGT CTC ATT GTT AAT AAT CCA TCA AAC CCC TGT GGG TCA GTG TTC AGT AAA AGT CAT CTC CAG AAA ATT CTG GCT GTG GCT GCA AGG CAG	[720]
	L E S L I D E K T A C L I V N N P S N P C G S V F S K S H L Q K I L A V A A R Q	[240]
pig	CTA GAG TCT CTG ATT GAT GAA AAG ACA GCT TGT CTT GTC GTC AAT AAT CCA TCA AAC CCT TGT GGG TCA GTG TTC AGT AGA AAT CAT CTC CAG AAA ATT TTG GCA GTG GCC ACC AGG CAG	[720]
	L E S L I D E K T A C L V V N N P S N P C G S V F S R N H L Q K I L A V A T R Q	[240]
panda	CTG GAA TCT TTG ATT GAT GAA AAG ACA GCT TGT CTC ATT GTT AAT AAT CCA TCA AAC CCC TGT GGG TCA GTG TTC AGT AAA AGT CAT CTC CAG AAA ATT CTG GCT GTG GCT GCA AAG CAG	[720]
	L E S L I D E K T A C L I V N N P S N P C G S V F S K S H L Q K I L A V A A K Q	[240]
horse	GTA GAA TCT CTG GTT GAT GAA AAG ACA GCT TGT CTC ATT GTC AAC AAT CCA TCA AAC CCC TGT GGG TCA GTG TTC AGT AAA AGT CAT CTC CAG AAA ATT CTG GCA GTG GCC GCA AGG CAG	[720]
	V E S L V D E K T A C L I V N N P S N P C G S V F S K S H L Q K I L A V A A R Q	[240]
Cynopterus sphinx	TTG GAA TCA CTG ATT GAT GAA AAG ACA GCT TGT CTC ATT GTC AAC AAC CCA TCA AAC CCC TGT GGG TCA GTG TTC AGT AAA AGT CAC CTC CAG GAT ATT CTG GCT GTG GCT GCA AGG CAG	[720]
	L E S L I D E K T A C L I V N N P S N P C G S V F S K S H L Q D I L A V A A R Q	[240]
Rousettus leschenaultii	ATG GAA TCA CTG ATC GAT GAA AAG ACA GCT TGT CTC ATT GTC AAC AAT CCA TCA AAC CCC TGT GGG TCA GTG TTC AGT AAA AGT CAC CTC CAG GAT ATT CTG GCT GTG GCT GCA AGG AAG	[720]
	M E S L I D E K T A C L I V N N P S N P C G S V F S K S H L Q D I L A V A A R K	[240]
Eonycteris spelaea	TTG GAA TCA CTG ATC GAT GAA AAG ACA GCT TGT CTC ATT GTC AAC AAT CCA TCA AAC CCC TGT GGG TCA GTG TTC AGT AAA AGT CAC CTC CAG GAT ATT CTG GCA GTG GCT GCA AGG AAG	[720]
	L E S L I D E K T A C L I V N N P S N P C G S V F S K S H L Q D I L A V A A R K	[240]
Pteropus vampyrus	TTG GAA TCA CTG ATT GAC GAA AAG ACA GCT TGT CTC ATT GTC AAC AAT CCA TCA AAC CCC TGT GGG TCA GTG TTC AGT AAA AGT CAC CTC CAG GAT ATT CTG GCT GTG GCT GCA AGG CAG	[720]
	L E S L I D E K T A C L I V N N P S N P C G S V F S K S H L Q D I L A V A A R Q	[240]
Rhinolophus ferrumequinum	TTA AAA TCT CTG ATT GAT GAA AAG ACA GCT TGT CTC ATT GTC AAC AAC CCA TCA AAT CCC TGT GGG TCA GTG TTT AGT AAA AAT CAT CTC CAG AAG ATT CTG GCC GTG GCT GCA AGG CAG	[720]
	L K S L I D E K T A C L I V N N P S N P C G S V F S K N H L Q K I L A V A A R Q	[240]
Rhinolophus pusillus	TTA GAA TCT CTG ATT GAT GAA AAG ACA GCT TGT CTC ATT GTC AAC AAC CCA TCA AAC CCC TGT GGG TCA GTG TTT AGT ACA AAT CAT CTC CAG AAG ATT CTG GCC GTG GCT GCA AGG CAG	[720]
	L E S L I D E K T A C L I V N N P S N P C G S V F S T N H L Q K I L A V A A R Q	[240]
Hipposideros armiger	TTG GAA GCT CTG ATT GAT GAA AAG ACA GCT TGT CTC ATT GTC AAT AAT CCA TCA AAC CCC TGT GGG TCA GTG TTC AGT AAA AGT CAT CTC CAG AAG ATT TTG GCC GTG GCT GCA AGG CAG	[720]
	L E A L I D E K T A C L I V N N P S N P C G S V F S K S H L Q K I L A V A A R Q	[240]
Hipposideros pratti	TTG GAA GCT CTG ATT GAT GAA AAG ACA GCT TGT CTC ATT GTC AAC AAT CCA TCA AAC CCC TGT GGG TCA GTG TTC AGT AAA AGT CAT CTC CAG AAG ATT TTG GCC GTG GCT GCA AGG CAG	[720]
	L E A L I D E K T A C L I V N N P S N P C G S V F S K S H L Q K I L A V A A R Q	[240]
Megaderma lyra	TTG GAA TCT CTA ATT GAT GAA AAG ACA GCT TGT CTC ATT GTC AAC AAC CCA TCA AAC CCC TGT GGG TCA GTA TTC AGT AAA AGT CAT CTC CAG AAG ATT TTG GCA GTG GCT GCA AGG CAG	[720]
	L E S L I D E K T A C L I V N N P S N P C G S V F S K S H L Q K I L A V A A R Q	[240]
Megaderma spasma	TTG GAA TCT CTA ATT GAT GAA AAG ACA GCT TGT CTC ATT GTT AAC AAC CCA TCA AAC CCC TGT GGG TCA GTA TTC AGT AAA AGT CAT CTC CAG AAG ATT TTG GCA GTG GCT GCA AGG CAG	[720]
	L E S L I D E K T A C L I V N N P S N P C G S V F S K S H L Q K I L A V A A R Q	[240]
Myotis ricketti	TTG GAA TCT CTG ATT GAT GAA AAG ACA GCT TGT CTC ATT GTC AAC AAT CCA TCA AAC CCC TGT GGG TCA GTG TTC AGC AGA AGT CAT CTT CAG AAG ATT CTA GCC GTG GCT GCA AGG CAG	[720]
	L E S L I D E K T A C L I V N N P S N P C G S V F S R S H L Q K I L A V A A R Q	[240]
Pipistrellus abramus	TTG GAA TCT CTG ATT GAC GAA AAG ACA GCT TGT CTC ATT GTC AAT AAT CCA TCA AAC CCC TGT GGG TCG GTG TTC AGC AGA AGT CAT CTC CAG AAG ATT CTG GCG GTG GCT GCA AGG CAG	[720]
	L E S L I D E K T A C L I V N N P S N P C G S V F S R S H L Q K I L A V A A R Q	[240]
Scotophilus kuhlii	TTG GAA TCT CTG ATT GAT GAA AAG ACA GCT TGT CTC ATT GTC AAT AAT CCA TCA AAC CCC TGT GGG TCA GTG TTC AGC AGA AGT CAT CTT CAG AAG ATT CTG GCC GTG GCT GCA AGG CAG	[720]
	L E S L I D E K T A C L I V N N P S N P C G S V F S R S H L Q K I L A V A A R Q	[240]
Miniopterus fuliginosus	CTG GAA TCT CTG ATT GAT GAA AAG ACA GCT TGT CTC ATT GTC AAT AAT CCA TCA AAC CCC TGT GGG TCA GTG TTC AGT AGG AGT CAT CTC CAG AAG ATT CTG GCT GTG GCT GCA AGG CAG	[720]
	L E S L I D E K T A C L I V N N P S N P C G S V F S R S H L Q K I L A V A A R Q	[240]
Tadarida plicata	CTG GAA TCT TTG ATT GAT GAA AAG ACA GCT TGT CTC ATT GTC AAT AAT CCA TCA AAC CCC TGT GGG TCA GTG TTC AGT AGA AGT CAT CTC CAG AAG ATT CTG GCT GTG GCT GCA AGG CAA	[720]
	L E S L I D E K T A C L I V N N P S N P C G S V F S R S H L Q K I L A V A A R Q	[240]
Mormoops megalophylla	TTG GAA TCT CTG ATT GAT GAA AAG ACA GCT TGT CTC ATC ATC AAT AAC CCG TCA AAC CCC TGT GGG TCA GTG TTC AGG AGA AGT CAT CTC CAG AAG ATT CTG GCC GTG GCT GCA AGG CAG	[720]
	L E S L I D E K T A C L I I N N P S N P C G S V F R R S H L Q K I L A V A A R Q	[240]
Pteronotus parnellii	TTG GAA TCT CTG ATT GAT GAA AAG ACA GCT TGT CTC ATT GTC AAT AAT CCA TCA AAC CCC TGT GGG TCA GTG TTC AGC AGA AGT CAT CTC CAG GAG ATT CTG GCC GTG GCT GCA AAG CAG	[720]
	L E S L I D E K T A C L I V N N P S N P C G S V F S R S H L Q E I L A V A A K Q	[240]
Desmodus rotundus	CTG GAA TCT CTG ATT GAT GAA AAG ACA GCT TGT CTC GTT GTC AAT AAT CCA TCA AAC CCC TGT GGG TCA GTG TTC AGC AGA AGT CAT CTC CAG AAG ATT CTG GCC GTG GCT GCG AGG CAG	[720]
	L E S L I D E K T A C L V V N N P S N P C G S V F S R S H L Q K I L A V A A R Q	[240]
Leptonycteris yerbabuena	TTA GAA TCT CTG ATT GAT GAA AAG ACA GCC TGT CTC ATT GTC AAT AAT CCA TCG AAC CCC TGT GGG TCA GTG TTC AGT AGA AGT CAT CTC CAG AAG ATT CTG GCC GTG GCT GCA CGG CAG	[720]
	L E S L I D E K T A C L I V N N P S N P C G S V F S R S H L Q K I L A V A A R Q	[240]
Artibeus lituratus	TTG GAA TCT CTG ATT GAT GAA AAG ACA GCC TGT CTC ATT GTC AAT AAT CCA TCG AAC CCC TGT GGG TCA GTG TTC AGT AGA AGT CAT CTC CAG AAG ATT CTG ACT GTG GCT GCA AGG CAG	[720]
	L E S L I D E K T A C L I V N N P S N P C G S V F S R S H L Q K I L T V A A R Q	[240]

S258T L263I S268R

human	TGT GTC CCC ATC TTA GCT GAT GAG ATC TAT GGA GAC ATG GTG TTT TCG GAT TGC AAA TAT GAA CCA CTG GCC ACC CTC AGC ACC GAT GTC CCC ATC CTG TCC TGT GGA GGG CTG GCC AAG	[840]
	C V P I L A D E I Y G D M V F S D C K Y E P L A T L S T D V P I L S C G G L A K	[280]
mouse	TGC GTC CCC ATC TTA GCC GAT GAG ATC TAT GGT GAC ATG GTG TTT TCA GAT TGC AAA TAT GAA CCA ATG GCC ACC CTC AGC ACC AAT GTC CCC ATC CTG TCC TGT GGT GGG CTG GCC AAG	[840]
	C V P I L A D E I Y G D M V F S D C K Y E P M A T L S T N V P I L S C G G L A K	[280]
rat	TGT GTC CCC ATC TTA GCT GAC GAG ATC TAT GGT GAC ATG GTG TTT TCA GAT TGC AAA TAC GAA CCA CTG GCC AAC CTC AGC ACC AAT GTT CCC ATC CTG TCC TGT GGT GGG CTG GCC AAG	[840]
	C V P I L A D E I Y G D M V F S D C K Y E P L A N L S T N V P I L S C G G L A K	[280]
cow	TGT GTT CCC ATT TTA GCT GAT GAG ATC TAT GGA GAC ATG GTG TTT TCA GAT TCC AAA TTT GAG CCT CTG GCC ACC CTC AGC AGC AAA GTC CCC ATC CTG TCC TGT GGA GGG CTG GCC AAG	[840]
	C V P I L A D E I Y G D M V F S D S K F E P L A T L S S K V P I L S C G G L A K	[280]
dog	TGC GTC CCC ATC TTA GCT GAT GAG ATC TAT GGA GAC ATG GTG TTT TCG GAT TCC AAA TTT GAG CCT CTG GCT ACC CTG AGC AGC AAC GTC CCC ATC CTG TCC TGT GGA GGG CTG GCC AAG	[840]
	C V P I L A D E I Y G D M V F S D S K F E P L A T L S S N V P I L S C G G L A K	[280]
pig	TGT GTC CCC ATC TTA GCT GAT GAG ATC TAT GGA GAC ATG GTG TTT TCA GAT TCC AAA TTT GAG CCT CTA GCC ACC CTG AGC AGC AAT GTC CCC ATC CTG TCC TGT GGA GGG CTG GCC AAG	[840]
	C V P I L A D E I Y G D M V F S D S K F E P L A T L S S N V P I L S C G G L A K	[280]
panda	TGT GTC CCC ATC TTA GCT GAT GAG ATC TAT GGA GAC ATG GTG TTT TCG GAT TCC AAA TTT GAG CCT TTG GCC ACC CTC AGC AGC AAC GTC CCC ATC CTG TCC TGT GGA GGG CTG GCC AAG	[840]
	C V P I L A D E I Y G D M V F S D S K F E P L A T L S S N V P I L S C G G L A K	[280]
horse	TGT GTC CCC ATC ATA GCT GAT GAG ATC TAT GGA GAC ATG GTG TTT TCA GAT TCC AAA TTT GAG CCT TTG GCC ACT CTC AGC AGC AAC GTT CCC ATC CTG TCC TGT GGA GGG TTA GCC AAG	[840]
	C V P I I A D E I Y G D M V F S D S K F E P L A T L S S N V P I L S C G G L A K	[280]
Cynopterus sphinx	TGT GTA CCC ATC GTG GCC GAT GAG ATT TAT GGA GAC ATG GTG TTT TCA GAT ACC AAA TTT GAG CCT ATA GCT ACT CTC AGC CGC AGC GTC CCT ATC CTG TGC TGT GGA GGG CTG ACC AAG	[840]
	C V P I V A D E I Y G D M V F S D T K F E P I A T L S R S V P I L C C G G L T K	[280]
Rousettus leschenaultii	TAT GTC CCC ATC TTA GCT GAT GAA ATC TAT GGA GAC ATG GTG TTT TCA GAT ACC AAA TTT GAG CCT ATA GCT ACT CTC AGC CGC AAC GTC CCC ATC CTG ACC TGT GGA GGG CTG GCC AAG	[840]
	Y V P I L A D E I Y G D M V F S D T K F E P I A T L S R N V P I L T C G G L A K	[280]
Eonycteris spelaea	TAT GTC CCC ATC TTA GCC GAT GAG ATC TAT GGA GAC ATG GTG TTT TCA GAT ACC AAA TTT GTG CCT ATA GCT ACT CTC AGC CGC AAC GTC CCC ATC CTG ACC TGT GGA GGG CTG GCC AAG	[840]
	Y V P I L A D E I Y G D M V F S D T K F V P I A T L S R N V P I L T C G G L A K	[280]
Pteropus vampyrus	TGT GTC CCC ATC TTA GCC GAT GAG ATC TAT GGA GAC ATG ATG TTT TCA GAT ACC AAA TTT GAG CCT ATA GCT ACT CTC AGC CAC AAC GTC CCC ATC CTG TCC TGT GGA GGG CTG GCC AAG	[840]
	C V P I L A D E I Y G D M M F S D T K F E P I A T L S H N V P I L S C G G L A K	[280]
Rhinolophus ferrumequinum	TGT GTC CCC ATC TTA GCT GAT GAG ATC TAT GGA GAC ATG GTG TTT TCA GAT TCC AAA TTT GAG CCT CTG GCC ACC CTC AGC AGC AAC GTC CCC ATC CTT TCC TGT GGA GGG CTG GCC AAG	[840]
	C V P I L A D E I Y G D M V F S D S K F E P L A T L S S N V P I L S C G G L A K	[280]
Rhinolophus pusillus	TGT GTC CCC ATC TTA GCT GAT GAG ATC TAT GGA GAC ATG GTG TTT TCA GAT TCC AAA TTT GAG CCT CTG GCC ACC CTC AGC AGC AAC GTC CCC ATC CTT TCC TGT GGA GGG CTG GCC AAG	[840]
	C V P I L A D E I Y G D M V F S D S K F E P L A T L S S N V P I L S C G G L A K	[280]
Hipposideros armiger	TGC GTC CCC ATC TTA GCT GAT GAG ATC TAT GGA GAC ATG GTG TTT TCA GAT TCC AAA TTT GAG CCT CTG GCC AAC CTC AGC ACC AAC GTC CCC ATC CTG TCC TGT GGA GGG CTG GCC AAG	[840]
	C V P I L A D E I Y G D M V F S D S K F E P L A N L S T N V P I L S C G G L A K	[280]
Hipposideros pratti	TGC GTC CCC ATC TTA GCT GAT GAG ATC TAT GGA GAC ATG GTG TTT TCA GAT TCC AAA TTT GAG CCT CTG GCC AAC CTC AGC ACC AAC GTC CCC ATC CTG TCC TGT GGA GGG CTG GCC AAG	[840]
	C V P I L A D E I Y G D M V F S D S K F E P L A N L S T N V P I L S C G G L A K	[280]
Megaderma lyra	TGT GTC CCC ATC TTA GCT GAT GAG ATC TAT GGA GAC ATG GTG TTC TCA GAT TCC AAG TTT GAG CCT CTG GCC ACG CTC AGC AGC AAT GTC CCC ATC CTG TCC TGT GGA GGG CTG GCT AAG	[840]
	C V P I L A D E I Y G D M V F S D S K F E P L A T L S S N V P I L S C G G L A K	[280]
Megaderma spasma	TGT GTC CCG ATC TTA GCT GAT GAG ATC TAT GGA GAC ATG GTG TTT TCA GAT TCC AAG TTT GAG CCT CTG GCC ACC CTC AGC GGC CAT GTC CCC ATC CTG TCC TGT GGA GGG CTG GCG AAG	[840]
	C V P I L A D E I Y G D M V F S D S K F E P L A T L S G H V P I L S C G G L A K	[280]
Myotis ricketti	TGT GTC CCC ATC TTA GCT GAT GAG ATC TAT GGA GAC ATG GTG TTT TCG GGC TCC AAA TTT GAG CCT CTG GCC ACC CTC AGC AGC AAC GTC CCC ATC CTG TCC TGC GGA GGG TTG GCC AAG	[840]
	C V P I L A D E I Y G D M V F S G S K F E P L A T L S S N V P I L S C G G L A K	[280]
Pipistrellus abramus	TGT GTG CCC ATC TTA GCT GAT GAG ATC TAT GGC GAT ATG GTG TTT TCG GGC TCC AAA TTT GAG CCT CTG GCC ACC CTC AGC ACC AAT GTC CCC ATC CTG TCC TGT GGA GGG CTG GCC AAG	[840]
	C V P I L A D E I Y G D M V F S G S K F E P L A T L S T N V P I L S C G G L A K	[280]
Scotophilus kuhlii	TGT GTC CCC ATC TTA GCT GAT GAG ATC TAT GGC GAC ATG GTG TTT TCA GGC TCC AAA TTT GAG CCT CTG GCC ACC CTC AGC AGC AAC GTC CCC ATC CTG TCC TGT GGA GGG TTG GCC AAG	[840]
	C V P I L A D E I Y G D M V F S G S K F E P L A T L S S N V P I L S C G G L A K	[280]
Miniopterus fuliginosus	TGT GTC CCC ATC TTA GCT GAT GAG ATC TAT GGA GAC ATG GTG TTT TCA GAT TCC AAA TTT GAG CCT CTG GCC ACC CTC AGC AGC AAT GTC CCC ATC CTG TCC TGT GGA GGG CTG GCC AAG	[840]
	C V P I L A D E I Y G D M V F S D S K F E P L A T L S S N V P I L S C G G L A K	[280]
Tadarida plicata	TGT GTC CCC ATC TTA GCT GAT GAG ATC TAT GGA GAC ATG GTA TTT TCA GAT TCC AAA TTT GAG CCT CTG GCC ACC CTC AGC AGC AAT GTC CCC ATC CTG TCC TGT GGA GGG CTG GCC AAG	[840]
	C V P I L A D E I Y G D M V F S D S K F E P L A T L S S N V P I L S C G G L A K	[280]
Mormoops megalophylla	TGC GTC CCC ATC TTA GCT GAT GAG ATC TAT GCA GAC ATG GTG TTT TCT GAT TCC AAA TTT GAG CCT CTG GCC AAC CTC AGC AGC GAT GTC CCC ATC CTG TCC TGT GGA GGG CTG GCC AAG	[840]
	C V P I L A D E I Y A D M V F S D S K F E P L A N L S S D V P I L S C G G L A K	[280]
Pteronotus parnellii	TGT GTC CCC ATC TTA GCT GAT GAG ATC TAT GGA GAC ATG GTG TTT TCT GAT TCC AAA TTT GAG CCT CTG GCC AAC CTC AGC AGC AAT GTC CCC ATC CTG TCC TGT GGA GGA TTG GCC AAG	[840]
	C V P I L A D E I Y G D M V F S D S K F E P L A N L S S N V P I L S C G G L A K	[280]
Desmodus rotundus	TGT GTC CCC ATC TTA GCT GAT GAG ATC TAC GGA GAC ATG GTG TTT TCT GAC TCC AAA TTT GAG CCT CTG GCC AGC CTC AGC AGC AAT GTC CCC ATC CTG TCC TGT GGA GGG TTG GCC AAG	[840]
	C V P I L A D E I Y G D M V F S D S K F E P L A S L S S N V P I L S C G G L A K	[280]
Leptonycteris yerbabuena	TGT GTA CCC ATC TTA GCC GAT GAG ATC TAT GGA GAC ATG GTG TTT TCC AAC TCC AAA TTT GAG CCT CTG GCC AAC CTC AGC AGC AAT GTC CCC ATC CTG TCC TGT GGA GGG TTG GCT AAG	[840]
	C V P I L A D E I Y G D M V F S N S K F E P L A N L S S N V P I L S C G G L A K	[280]
Artibeus lituratus	TGT GTC CCC ATC TTA GCT GAT GAG ATC TAT GGA GAC ATG GTG TTT TCT GAC TCC AAA TTT GAG CCT CTG GCC AAC CTC AGC AGC AAT GTC CCC ATC CTG TCC TGT GGA GGG TTG GCC AAG	[840]
	C V P I L A D E I Y G D M V F S D S K F E P L A N L S S N V P I L S C G G L A K	[280]

																										D307N										L317M				
human	CGC	TGG	CTG	GTT	CCT	GGC	TGG	AGG	TTG	GGC	TGG	ATC	CTC	ATT	CAT	GAC	CGA	AGA	GAC	ATT	TTT	GGC	AAT	GAG	ATC	CGA	GAT	GGG	CTG	GTG	AAG	CTG	AGT	CAG	CGC	ATT	TTG	GGA	CCC	TGT	[960]
	R	W	L	V	P	G	W	R	L	G	W	I	L	I	H	D	R	R	D	I	F	G	N	E	I	R	D	G	L	V	K	L	S	Q	R	I	L	G	P	C	[320]
mouse	CGC	TGG	CTG	GTT	CCT	GGC	TGG	AGG	CTG	GGC	TGG	ATC	CTT	ATC	CAT	GAT	CGA	AGA	GAC	ATT	TTT	GGC	AAT	GAG	ATT	CGG	GAC	GGG	CTG	GTG	AAG	CTG	AGT	CAG	CGG	ATC	CTG	GGC	CCG	TGC	[960]
	R	W	L	V	P	G	W	R	L	G	W	I	L	I	H	D	R	R	D	I	F	G	N	E	I	R	D	G	L	V	K	L	S	Q	R	I	L	G	P	C	[320]
rat	CGC	TGG	CTG	GTT	CCT	GGC	TGG	AGG	TTG	GGC	TGG	ATC	CTC	ATT	CAT	GAT	CGA	AGA	GAC	ATT	TTT	GGC	AAT	GAG	ATT	CGA	GAC	GGG	CTG	GTG	AAA	CTG	AGT	CAG	CGG	ATC	CTG	GGA	CCA	TGC	[960]
	R	W	L	V	P	G	W	R	L	G	W	I	L	I	H	D	R	R	D	I	F	G	N	E	I	R	D	G	L	V	K	L	S	Q	R	I	L	G	P	C	[320]
cow	CGC	TGG	CTG	GTT	CCT	GGC	TGG	AGG	ATG	GGC	TGG	ATC	CTT	ATC	CAT	GAC	CGA	AGA	GAT	ATT	TTT	GGC	AAT	GAG	ATC	CGA	GAT	GGG	CTG	ACG	AAG	CTA	AGT	CAA	CGG	ATC	CTG	GGC	CCC	TGC	[960]
	R	W	L	V	P	G	W	R	M	G	W	I	L	I	H	D	R	R	D	I	F	G	N	E	I	R	D	G	L	T	K	L	S	Q	R	I	L	G	P	C	[320]
dog	CGC	TGG	TTG	GTT	CCT	GGC	TGG	AGG	TTG	GGC	TGG	ATC	CTC	ATC	CAT	GAC	CGA	AGA	GAC	ATT	TTT	GGC	AAT	GAG	ATC	CGA	GAT	GGG	CTG	GTG	AAG	CTG	AGT	CAG	AGG	ATC	CTG	GGG	CCC	TGC	[960]
	R	W	L	V	P	G	W	R	L	G	W	I	L	I	H	D	R	R	D	I	F	G	N	E	I	R	D	G	L	V	K	L	S	Q	R	I	L	G	P	C	[320]
pig	CGC	TGG	CTG	GTT	CCT	GGC	TGG	AGG	CTG	GGC	TGG	ATC	CTC	ATC	CAC	GAT	CGC	AGG	GAC	ATT	TTT	GGC	AAT	GAG	ATC	CGA	GAT	GGG	CTG	GTG	AAG	CTG	AGT	CAG	CGA	ATC	CTG	GGA	CCC	TGC	[960]
	R	W	L	V	P	G	W	R	L	G	W	I	L	I	H	D	R	R	D	I	F	G	N	E	I	R	D	G	L	V	K	L	S	Q	R	I	L	G	P	C	[320]
panda	CGC	TGG	CTG	GTT	CCT	GGC	TGG	AGG	TTG	GGC	TGG	ATC	CTC	ATC	CAT	GAC	CGA	AGA	GAC	ATT	TTT	GGC	AAT	GAG	ATC	CGA	GAT	GGG	CTG	GTG	AAG	CTG	AGT	CAG	CGG	ATC	CTG	GGA	CCC	TGC	[960]
	R	W	L	V	P	G	W	R	L	G	W	I	L	I	H	D	R	R	D	I	F	G	N	E	I	R	D	G	L	V	K	L	S	Q	R	I	L	G	P	C	[320]
horse	CGC	TGG	CTG	GTT	CCC	GGC	TGG	AGG	TTG	GGC	TGG	ATC	CTC	ATC	CAT	GAC	CGA	AGA	GAC	ATT	TTT	GGC	AAC	GAG	ATC	CGA	GAT	GGG	CTG	GTG	AAG	CTG	AGT	CAG	CGG	ATC	CTG	GGA	CCC	TGC	[960]
	R	W	L	V	P	G	W	R	L	G	W	I	L	I	H	D	R	R	D	I	F	G	N	E	I	R	D	G	L	V	K	L	S	Q	R	I	L	G	P	C	[320]
Cynopterus sphinx	CGT	TGG	CTG	GTT	CCT	GGC	TGG	AGG	TTG	GGC	TGG	ATC	CTC	ATC	CAT	GAC	CGA	GGA	GAC	ATT	TTT	GGC	AAT	GAG	ATC	CGA	AAC	GGG	CTG	GTA	AAG	CTG	AGT	CAG	CGG	ATC	ATG	GGA	CCC	TGC	[960]
	R	W	L	V	P	G	W	R	L	G	W	I	L	I	H	D	R	G	D	I	F	G	N	E	I	R	N	G	L	V	K	L	S	Q	R	I	M	G	P	C	[320]
Rousettus leschenaultii	CGC	TGG	CTG	GTT	CCT	GGC	TGG	AGG	CTG	GGC	TGG	ATC	CTC	ATC	CAT	GAC	CGA	AGA	GAC	ATT	TTC	GGC	AAT	GAG	ATC	CGA	AAC	GGG	CTG	GTG	AAG	TTG	TGT	CAG	CGG	ATC	ATG	GGA	CCC	TGC	[960]
	R	W	L	V	P	G	W	R	L	G	W	I	L	I	H	D	R	R	D	I	F	G	N	E	I	R	N	G	L	V	K	L	C	Q	R	I	M	G	P	C	[320]
Eonycteris spelaea	CGC	TGG	CTG	GTT	CCT	GGC	TGG	AGG	TTG	GGC	TGG	ATC	TTC	ATC	CAT	GAC	CGA	AAA	GAC	ATT	TTT	GGC	AAT	GAG	ATC	CGA	AAC	GGG	CTG	GTG	AGG	CTG	TGT	CAG	CGG	ATT	ATG	GGA	CCC	TGC	[960]
	R	W	L	V	P	G	W	R	L	G	W	I	F	I	H	D	R	K	D	I	F	G	N	E	I	R	N	G	L	V	R	L	C	Q	R	I	M	G	P	C	[320]
Pteropus vampyrus	CGC	TGG	CTG	GTT	CCT	GGC	TGG	AGG	TTG	GGC	TGG	ATC	CTC	ATC	CAT	GAC	CGA	GGA	GAC	ATT	TTT	GGC	AAT	GAG	ATC	CCA	AAC	GGG	CTG	GTG	AAG	CTG	AGT	CAG	CGG	ATC	ATG	GGA	CCC	TGC	[960]
	R	W	L	V	P	G	W	R	L	G	W	I	L	I	H	D	R	G	D	I	F	G	N	E	I	P	N	G	L	V	K	L	S	Q	R	I	M	G	P	C	[320]
Rhinolophus ferrumequinum	CGC	TGG	CTG	GTT	CCC	GGC	TGG	AGA	TTG	GGC	TGG	ATC	CTC	ATC	CAT	GAC	CGA	AAA	GAT	ATT	TTT	GGC	AAT	GAG	ATC	CGA	GAT	GGA	CTG	GTG	AAG	CTG	AGT	CAG	CGA	ATC	CTG	GGA	CCC	TGC	[960]
	R	W	L	V	P	G	W	R	L	G	W	I	L	I	H	D	R	K	D	I	F	G	N	E	I	R	D	G	L	V	K	L	S	Q	R	I	L	G	P	C	[320]
Rhinolophus pusillus	CGC	TGG	CTG	GTT	CCC	GGC	TGG	AGA	CTG	GGC	TGG	ATC	CTC	ATC	CAT	GAC	CGA	AAA	GAT	ATT	TTT	GGC	AAC	GAG	ATC	CGA	GAT	GGA	CTG	GTG	AAG	CTG	AGT	CAG	CGG	ATC	CTG	GGA	CCC	TGC	[960]
	R	W	L	V	P	G	W	R	L	G	W	I	L	I	H	D	R	K	D	I	F	G	N	E	I	R	D	G	L	V	K	L	S	Q	R	I	L	G	P	C	[320]
Hipposideros armiger	CGC	TGG	CTG	GTG	CCT	GGC	TGG	AGA	CTG	GGC	TGG	ATC	CTC	ATC	CAT	GAC	CGA	AGA	AAC	ATT	TTT	GGC	AAT	GAG	ATC	CGA	GAT	GGA	CTG	GTG	AAG	CTG	AGT	CAG	CGG	ATC	CTG	GGA	CCT	TGC	[960]
	R	W	L	V	P	G	W	R	L	G	W	I	L	I	H	D	R	R	N	I	F	G	N	E	I	R	D	G	L	V	K	L	S	Q	R	I	L	G	P	C	[320]
Hipposideros pratti	CGC	TGG	CTG	GTG	CCC	GGC	TGG	AGA	CTG	GGC	TGG	ATC	CTC	ATC	CAT	GAC	CGA	AGA	AAC	ATT	TTT	GGC	AAT	GAG	ATC	CGA	GAT	GGA	CTG	GTG	AAG	CTG	AGT	CAG	CGG	ATC	CTG	GGA	CCT	TGC	[960]
	R	W	L	V	P	G	W	R	L	G	W	I	L	I	H	D	R	R	N	I	F	G	N	E	I	R	D	G	L	V	K	L	S	Q	R	I	L	G	P	C	[320]
Megaderma lyra	CGC	TGG	TTG	GTT	CCT	GGC	TGG	AGG	TTG	GGC	TGG	ATC	CTC	ATC	CAT	GAC	CGA	AGA	GAC	ATT	TTT	GGT	AAT	GAG	ATC	CGA	GAT	GGA	CTG	GTG	AAG	CTG	AGT	CAG	CGT	ATC	CTG	GGG	CCC	TGC	[960]
	R	W	L	V	P	G	W	R	L	G	W	I	L	I	H	D	R	R	D	I	F	G	N	E	I	R	D	G	L	V	K	L	S	Q	R	I	L	G	P	C	[320]
Megaderma spasma	CGC	TGG	CTG	GTT	CCC	GGC	TGG	AGG	CTG	GGC	TGG	ATC	CTC	ATC	CAT	GAC	CGA	AGA	GAC	ATT	TTT	GGC	AAT	GAG	ATC	CGA	GAT	GGA	CTG	GTG	AAG	CTG	AGT	CAG	CGT	ATC	CTG	GGG	CCC	TGC	[960]
	R	W	L	V	P	G	W	R	L	G	W	I	L	I	H	D	R	R	D	I	F	G	N	E	I	R	D	G	L	V	K	L	S	Q	R	I	L	G	P	C	[320]
Myotis ricketti	CGC	TGG	CTG	GTT	CCT	GGC	TGG	AGG	TTG	GGC	TGG	ATC	CTC	ATC	CAT	GAC	CGC	AGG	GAC	ATT	TTT	GGC	AAT	GAG	ATC	CGA	GAT	GGG	CTG	GTG	AAG	CTG	AGT	CAG	CGG	ATC	CTG	GGA	CCC	TGC	[960]
	R	W	L	V	P	G	W	R	L	G	W	I	L	I	H	D	R	R	D	I	F	G	N	E	I	R	D	G	L	V	K	L	S	Q	R	I	L	G	P	C	[320]
Pipistrellus abramus	CGC	TGG	CTG	GTT	CCT	GGC	TGG	AGG	CTG	GGC	TGG	ATC	CTC	ATC	CAT	GAC	CGA	AGG	GAC	ATT	TTT	GGC	AAT	GAG	ATC	CGA	GAT	GGG	CTG	GTG	AAG	CTG	AGT	CAG	CGG	ATC	TTG	GGA	CCG	TGC	[960]
	R	W	L	V	P	G	W	R	L	G	W	I	L	I	H	D	R	R	D	I	F	G	N	E	I	R	D	G	L	V	K	L	S	Q	R	I	L	G	P	C	[320]
Scotophilus kuhlii	CGC	TGG	CTG	GTT	CCT	GGC	TGG	AGG	TTG	GGC	TGG	ATC	CTC	ATC	CAT	GAC	CGA	AGG	GAC	ATT	TTT	GGC	AAT	GAG	ATC	CGA	GAC	GGG	CTG	GTG	AAG	CTG	AGT	CAG	CGG	ATC	CTG	GGG	CCC	TGC	[960]
	R	W	L	V	P	G	W	R	L	G	W	I	L	I	H	D	R	R	D	I	F	G	N	E	I	R	D	G	L	V	K	L	S	Q	R	I	L	G	P	C	[320]
Miniopterus fuliginosus	CGC	TGG	CTG	GTT	CCT	GGC	TGG	AGG	TTG	GGC	TGG	ATC	CTC	ATT	CAT	GAC	CGA	AAA	GGC	ATT	TTT	GGC	AAT	GAG	ATC	CGA	GAC	GGG	CTG	GTA	AAG	CTG	AGT	CAG	CGG	ATC	CTG	GGA	CCC	TGC	[960]
	R	W	L	V	P	G	W	R	L	G	W	I	L	I	H	D	R	K	G	I	F	G	N	E	I	R	D	G	L	V	K	L	S	Q	R	I	L	G	P	C	[320]
Tadarida plicata	CGC	TGG	CTG	GTT	CCT	GGC	TGG	AGG	TTG	GGC	TGG	ATT	CTC	ATC	CAT	GAC	CGA	AGA	GAT	ATT	TTT	GGC	AAT	GAG	ATC	CGA	GAT	GGG	CTG	GTG	AAG	CTG	AGT	CAG	CGG	ATC	CTG	GGA	CCC	TGC	[960]
	R	W	L	V	P	G	W	R	L	G	W	I	L	I	H	D	R	R	D	I	F	G	N	E	I	R	D	G	L	V	K	L	S	Q	R	I	L	G	P	C	[320]
Mormoops megalophylla	CGC	TGG	TTG	GTT	CCT	GGC	TGG	AGG	CTG	GGC	TGG	ATC	CTC	ATC	CAT	GAC	CGA	AGA	GAC	ATT	TTT	GGC	AAT	GAG	ATC	CGA	GAT	GGG	CTG	GTG	AAG	CTG	AGC	CAG	CGG	ATC	CTG	GGA	CCC	TGC	[960]
	R	W	L	V	P	G	W	R	L	G	W	I	L	I	H	D	R	R	D	I	F	G	N	E	I	R	D	G	L	V	K	L	S	Q	R	I	L	G	P	C	[320]
Pteronotus parnellii	CGC	TGG	TTG	GTT	CCT	GGC	TGG	AGG	TTG	GGC	TGG	ATC	CTC	ATC	CAT	GAC	CGA	AGA	GAC	ATT	TTT	GGC	AAT	GAG	ATT	CGA	GAT	GGT	CTG	GTG	AAG	CTG	AGT	CAG	CGG	ATC	CTG	GGA	CCC	TGC	[960]
	R	W	L	V	P	G	W	R	L	G	W	I	L	I	H	D	R	R	D	I	F	G	N	E	I	R	D	G	L	V	K	L	S	Q	R	I	L	G	P	C	[320]
Desmodus rotundus	CGC	TGG	CTG	GTT	CCT	GGC	TGG	AGG	ATG	GGC	TGG	ATC	CTC	ATC	CAT	GAC	CGA	AGA	GAC	ATT	TTT	GGC	AAT	GAG	ATC	CGA	GAT	GGG	CTG	GTG	GAG	CTG	AGT	CAG	CGG	ATC	CTG	GGA	CCC	TGT	[960]
	R	W	L	V	P	G	W	R	M	G	W	I	L	I	H	D	R	R	D	I	F	G	N	E	I	R	D	G	L	V	E	L	S	Q	R	I	L	G	P	C	[320]
Leptonycteris yerbabuena	CGC	TGG	CTG	GTT	CCT	GGC	TGG	AGG	TTG	GGC	TGG	ATC	CTT	GTC	CAT	GAC	CGA	AGA	GAC	ATT	TTT	GGC	AAT	GAG	ATC	CGA	GAT	GGG	CTG	GTG	AAG	CTG	AGC	CAG	CGG	ATC	CTG	GGA	CCC	TGC	[960]
	R	W	L	V	P	G	W	R	L	G	W	I	L	V	H	D	R	R	D	I	F	G	N	E	I	R	D	G	L	V	K	L	S	Q	R	I	L	G	P	C	[320]
Artibeus lituratus	CGC	TGG	CTG	GTT	CCT	GGC	TGG	AGG	TTG	GGC	TGG	ATC	CTC	ATT	CAT	GAC	CGA	AGA	GAC	ATT	TTT	GGC	AAT	GAG	ATC	CGA	GAT	GGG	CTG	GTA	AAG	CTG	AGC	CAG	AGA	ATC	CTG	GGA	CCC	TGC	[960]
	R	W	L	V	P	G	W	R	L	G	W	I	L	I	H	D	R	R	D	I	F	G	N	E	I	R	D	G	L	V	K	L	S	Q	R	I	L	G	P	C	[320]

																																			G355E						
human	ACC	ATT	GTC	CAG	GGA	GCT	CTG	AAA	AGC	ATC	CTA	TGT	CGC	ACC	CCG	GGA	GAG	TTT	TAC	CAC	AAC	ACT	CTG	AGC	TTC	CTC	AAG	TCC	AAT	GCT	GAT	CTC	TGT	TAT	GGG	GCG	TTG	GCT	GCC	ATC	[1080]
	T	I	V	Q	G	A	L	K	S	I	L	C	R	T	P	G	E	F	Y	H	N	T	L	S	F	L	K	S	N	A	D	L	C	Y	G	A	L	A	A	I	[360]
mouse	ACC	ATC	GTC	CAA	GGT	GCC	CTG	AAG	AGC	ATC	CTT	CAG	CGC	ACC	CCT	CAG	GAG	TTC	TAC	CAG	GAC	ACT	TTA	AGC	TTC	CTT	AAG	TCC	AAT	GCG	GAC	CTC	TGC	TAT	GGG	GCG	TTG	TCT	GCC	ATT	[1080]
	T	I	V	Q	G	A	L	K	S	I	L	Q	R	T	P	Q	E	F	Y	Q	D	T	L	S	F	L	K	S	N	A	D	L	C	Y	G	A	L	S	A	I	[360]
rat	ACC	ATA	GTC	CAG	GGT	GCT	CTG	AAG	AGC	ATC	CTT	CAG	CGA	ACC	CCT	CAG	GAG	TTC	TAT	CAC	GAC	ACG	TTA	AGC	TTC	CTC	AAG	TCC	AAT	GCG	GAC	CTC	TGC	TAT	GGG	GCA	CTG	GCT	GCC	ATC	[1080]
	T	I	V	Q	G	A	L	K	S	I	L	Q	R	T	P	Q	E	F	Y	H	D	T	L	S	F	L	K	S	N	A	D	L	C	Y	G	A	L	A	A	I	[360]
cow	ACC	CTT	GTC	CAG	GGA	GCT	CTG	AAA	AGC	ATC	CTG	TGT	CGC	ACC	CCT	CGT	GTG	TTC	TAC	CAC	AAC	ACT	CTA	AGC	TTC	CTC	AAG	TCC	AAT	GCG	GAT	CTC	TGC	TAT	GGG	GCA	TTG	GCT	GCC	ATC	[1080]
	T	L	V	Q	G	A	L	K	S	I	L	C	R	T	P	R	V	F	Y	H	N	T	L	S	F	L	K	S	N	A	D	L	C	Y	G	A	L	A	A	I	[360]
dog	ACC	ATC	GTC	CAG	GGA	GCT	CTG	AAA	AGC	ATC	CTG	CGT	CGC	ACC	CCT	CAG	GAG	TTC	TAC	CAA	AAT	ACT	CTG	AGC	TTC	CTG	AAG	TCC	AAT	GCT	GAT	CTT	TGC	TAT	GGA	GCT	TTG	GCT	GCC	ATT	[1080]
	T	I	V	Q	G	A	L	K	S	I	L	R	R	T	P	Q	E	F	Y	Q	N	T	L	S	F	L	K	S	N	A	D	L	C	Y	G	A	L	A	A	I	[360]
pig	ACC	CTG	GTC	CAG	GGA	GCT	CTG	AAA	AGC	ATC	CTG	CGC	CGC	ACC	CCT	CAA	GAG	TTC	TAC	CAT	AAC	ACT	CTA	AGC	TTC	CTC	AAG	ACC	AAC	GCT	GAC	CTC	TGC	TAT	GGG	GCC	TTG	GCT	GCT	GTC	[1080]
	T	L	V	Q	G	A	L	K	S	I	L	R	R	T	P	Q	E	F	Y	H	N	T	L	S	F	L	K	T	N	A	D	L	C	Y	G	A	L	A	A	V	[360]
panda	ACC	ATC	GTC	CAG	GGA	GCT	CTG	AAA	AGC	ATC	CTG	CAT	CGC	ACC	CCT	CAG	GAG	TTC	TAC	CAC	AAC	ACT	CTG	AGC	TTC	CTG	AAG	TCC	AAT	GCT	GAT	CTT	TGC	TAT	GGG	GCA	TTG	GCT	GCC	ATC	[1080]
	T	I	V	Q	G	A	L	K	S	I	L	H	R	T	P	Q	E	F	Y	H	N	T	L	S	F	L	K	S	N	A	D	L	C	Y	G	A	L	A	A	I	[360]
horse	ACC	ATT	GTC	CAG	GGA	GCT	CTG	AAA	AAT	ATC	CTG	CGT	CGC	ACC	CCT	CAA	GAG	TTC	TAC	CAC	AAC	ACT	CTA	AGC	TTA	CTC	AAG	TCC	AAT	GCG	GAT	CTC	TGC	TAC	GGT	GCA	CTG	GCT	GCC	ATC	[1080]
	T	I	V	Q	G	A	L	K	N	I	L	R	R	T	P	Q	E	F	Y	H	N	T	L	S	L	L	K	S	N	A	D	L	C	Y	G	A	L	A	A	I	[360]
Cynopterus sphinx	ACC	CTT	GTC	CAG	GGA	GCT	CTG	AAA	AGC	ATT	CTG	CGT	CGC	ACC	CCT	CAA	GAG	TTC	TAC	CAC	AAC	ACT	TTA	AGC	TTC	CTC	AAG	TCC	AGT	GCT	GAC	CTG	TGC	TAT	GTG	GCA	TTG	TCT	GCC	ATC	[1080]
	T	L	V	Q	G	A	L	K	S	I	L	R	R	T	P	Q	E	F	Y	H	N	T	L	S	F	L	K	S	S	A	D	L	C	Y	V	A	L	S	A	I	[360]
Rousettus leschenaultii	ACC	ATT	GTC	CAG	GGA	GCT	CTG	AAA	AGC	ATT	CTG	CGT	CGC	ACC	CCT	CAA	GAG	TTC	TAC	CAC	AAC	ACT	TTA	AGC	TTC	CTC	AAG	TCC	AAT	GCT	GAT	CTG	TGC	TAT	GAG	GCA	TTG	GCT	GCC	ATC	[1080]
	T	I	V	Q	G	A	L	K	S	I	L	R	R	T	P	Q	E	F	Y	H	N	T	L	S	F	L	K	S	N	A	D	L	C	Y	E	A	L	A	A	I	[360]
Eonycteris spelaea	ACC	ATC	GTC	CAG	GGA	GCT	CTG	AAA	AGC	ATT	CTG	CGT	CGT	ACC	CCT	CAA	GAG	TTC	TAC	CAC	AAC	ACT	TTA	AGC	TTC	CTC	AAG	TCC	AGT	GCT	GAT	CTG	TGC	TAT	GAG	GCA	TTG	GCT	GCC	ATC	[1080]
	T	I	V	Q	G	A	L	K	S	I	L	R	R	T	P	Q	E	F	Y	H	N	T	L	S	F	L	K	S	S	A	D	L	C	Y	E	A	L	A	A	I	[360]
Pteropus vampyrus	ACC	GTT	GTC	CAG	GGA	GCT	CTG	AAG	AGC	ATT	CTG	CGT	CGC	ACC	CCT	CAA	GAG	TTC	TAC	CGC	AAC	ACT	TTA	AGC	GTC	CTC	AAG	TCC	AGT	GCT	GAT	CTG	TGC	TAT	GAG	GCA	TTG	TCT	GCC	ATC	[1080]
	T	V	V	Q	G	A	L	K	S	I	L	R	R	T	P	Q	E	F	Y	R	N	T	L	S	V	L	K	S	S	A	D	L	C	Y	E	A	L	S	A	I	[360]
Rhinolophus ferrumequinum	ACC	ATT	GTC	CAG	GGA	GCT	CTG	AAA	AGC	ATC	CTG	CAT	CGC	ACC	CCT	CAA	GAG	TTC	TAC	CAC	AAC	ACT	CTA	AGC	TTC	CTC	AAG	TCC	AAT	GCG	GAT	CTG	TGC	TAT	GGG	GCA	TTG	GCT	GCC	ATC	[1080]
	T	I	V	Q	G	A	L	K	S	I	L	H	R	T	P	Q	E	F	Y	H	N	T	L	S	F	L	K	S	N	A	D	L	C	Y	G	A	L	A	A	I	[360]
Rhinolophus pusillus	ACC	ATT	GTC	CAG	GGA	GCT	CTG	AAA	AGC	ATC	CTG	CAT	CGC	ACC	CCT	CAA	GAG	TTC	TAC	CAC	AAC	ACT	CTA	AGC	TTC	CTC	AAG	TCC	AAT	GCG	GAT	CTG	TGC	TAT	GGG	GCA	TTG	GCT	GCC	ATC	[1080]
	T	I	V	Q	G	A	L	K	S	I	L	H	R	T	P	Q	E	F	Y	H	N	T	L	S	F	L	K	S	N	A	D	L	C	Y	G	A	L	A	A	I	[360]
Hipposideros armiger	ACC	CTT	GTC	CAG	GGA	GCT	CTG	AAA	AAC	ATC	CTG	CGT	CGC	ACC	CCT	CAA	GAA	TTC	TAC	CAC	AAC	ACT	CTA	AGC	TTC	CTC	AAG	TCC	AAT	GCT	GAT	CTG	TGC	TAT	GGG	GCG	TTG	TCT	GCC	ATC	[1080]
	T	L	V	Q	G	A	L	K	N	I	L	R	R	T	P	Q	E	F	Y	H	N	T	L	S	F	L	K	S	N	A	D	L	C	Y	G	A	L	S	A	I	[360]
Hipposideros pratti	ACC	CTT	GTC	CAG	GGA	GCT	CTG	AAA	AAC	ATC	CTG	CGT	CGC	ACC	CCT	CAA	GAA	TTC	TAC	CAC	AAC	ACT	CTA	AGC	TTC	CTC	AAG	TCC	AAT	GCT	GAT	CTG	TGC	TAT	GGG	GCA	TTG	TCT	GCC	ATC	[1080]
	T	L	V	Q	G	A	L	K	N	I	L	R	R	T	P	Q	E	F	Y	H	N	T	L	S	F	L	K	S	N	A	D	L	C	Y	G	A	L	S	A	I	[360]
Megaderma lyra	ACC	ATT	GTC	CAG	GGA	GCT	CTG	AAA	AGC	ATC	CTG	CGT	CGC	ACC	CCT	CAA	GAG	TTC	TAC	CAC	AAC	ACT	CTA	AGC	TTC	CTC	AAG	TCT	AAT	GCT	GAT	CTG	TGC	TAT	GGG	GCA	CTG	GCT	GCC	ATC	[1080]
	T	I	V	Q	G	A	L	K	S	I	L	R	R	T	P	Q	E	F	Y	H	N	T	L	S	F	L	K	S	N	A	D	L	C	Y	G	A	L	A	A	I	[360]
Megaderma spasma	ACC	ATT	GTC	CAG	GGA	GCT	CTG	AAA	AGC	ATC	CTG	CGT	CGC	ACC	CCT	CAA	GAG	TTC	TAC	CAC	AAC	ACT	CTA	AGC	TTC	CTC	AAG	TCC	AAT	GCC	GAT	CTG	TGC	TAT	GGG	GCA	CTG	GCT	GCC	ATC	[1080]
	T	I	V	Q	G	A	L	K	S	I	L	R	R	T	P	Q	E	F	Y	H	N	T	L	S	F	L	K	S	N	A	D	L	C	Y	G	A	L	A	A	I	[360]
Myotis ricketti	ACC	ATT	GTC	CAG	GGA	GCT	TTG	AAA	AGC	ATC	CTG	CGT	CGC	ACC	CCT	CAA	GAG	TTC	TAC	CAC	GAC	ACC	CTA	AGC	TTC	CTC	AAG	TCC	AAT	GCT	GAC	CTG	TGC	TAT	GGG	GCG	TTG	GCT	GCC	ATC	[1080]
	T	I	V	Q	G	A	L	K	S	I	L	R	R	T	P	Q	E	F	Y	H	D	T	L	S	F	L	K	S	N	A	D	L	C	Y	G	A	L	A	A	I	[360]
Pipistrellus abramus	ACC	ATT	GTC	CAG	GGA	GCT	TTG	AAA	AGC	ATC	CTG	CGT	CGC	ACC	CCT	CAA	GAG	TTC	TAC	CAC	GAC	ACC	CTA	AGC	TTC	CTC	AGG	TCC	AAT	GCT	GAT	CTG	TGC	TAT	GGG	GCG	TTG	GCT	GCC	ATT	[1080]
	T	I	V	Q	G	A	L	K	S	I	L	R	R	T	P	Q	E	F	Y	H	D	T	L	S	F	L	R	S	N	A	D	L	C	Y	G	A	L	A	A	I	[360]
Scotophilus kuhlii	ACC	ATT	GTC	CAG	GGA	GCT	TTG	AAA	AGC	ATC	CTG	CGT	CGC	ACC	CCT	CAA	GAG	TTC	TAC	CAC	GAC	ACC	CTA	AGC	TTC	CTC	AAG	TCC	AAT	GCT	GAT	CTG	TGC	TAT	GGG	GCG	TTG	GCT	GCA	ATC	[1080]
	T	I	V	Q	G	A	L	K	S	I	L	R	R	T	P	Q	E	F	Y	H	D	T	L	S	F	L	K	S	N	A	D	L	C	Y	G	A	L	A	A	I	[360]
Miniopterus fuliginosus	ACT	ATT	GTC	CAG	GGA	GCT	TTG	AAA	AGC	ATC	CTG	CAT	CGC	ACC	ACT	CAA	GAG	TTC	TTC	CAG	GAT	ACT	CTA	AGC	TTC	CTC	AAG	TCC	AAT	GCT	GAC	CTG	TGC	TAT	GGG	GCG	TTG	GCT	GCC	ATC	[1080]
	T	I	V	Q	G	A	L	K	S	I	L	H	R	T	T	Q	E	F	F	Q	D	T	L	S	F	L	K	S	N	A	D	L	C	Y	G	A	L	A	A	I	[360]
Tadarida plicata	ACC	ATT	GTC	CAG	GGA	GCT	TTG	AAA	AGC	ATC	CTG	CGT	CGC	ACC	CCT	CAA	GAG	TTC	TAC	CAC	AAC	ACT	CTA	AGC	TTC	CTC	AAG	TCC	AAT	GCT	GAT	CTG	TGC	TAT	GGG	GCA	TTG	GCT	GCC	ATC	[1080]
	T	I	V	Q	G	A	L	K	S	I	L	R	R	T	P	Q	E	F	Y	H	N	T	L	S	F	L	K	S	N	A	D	L	C	Y	G	A	L	A	A	I	[360]
Mormoops megalophylla	ACC	ATT	GTC	CAG	GGG	GCT	TTG	AAA	AGC	ATC	CTG	CGT	CGT	ACC	CCT	CGG	GAG	TTC	TAC	CAC	AAC	ACT	CTA	AGC	TTC	CTC	AAG	TCC	AGT	GCT	GAT	CTG	TGC	TAT	GGA	GCA	CTG	GCT	GCC	ATC	[1080]
	T	I	V	Q	G	A	L	K	S	I	L	R	R	T	P	R	E	F	Y	H	N	T	L	S	F	L	K	S	S	A	D	L	C	Y	G	A	L	A	A	I	[360]
Pteronotus parnellii	ACC	ATT	GTC	CAG	GGA	GCT	TTG	AAA	AGC	ATC	CTG	CGC	CGC	ACC	CCC	CAA	GAG	TTC	TAC	CAC	AAC	ACG	CTA	AGC	TTC	CTC	AAG	TCC	AAT	GCT	GAT	CTG	TGC	TAC	GGG	GCG	TTG	GCT	GCT	GTC	[1080]
	T	I	V	Q	G	A	L	K	S	I	L	R	R	T	P	Q	E	F	Y	H	N	T	L	S	F	L	K	S	N	A	D	L	C	Y	G	A	L	A	A	V	[360]
Desmodus rotundus	ACC	ATT	GTT	CAG	GGA	GCT	TTG	AAA	AGC	ATC	CTG	CGT	CGC	ACC	CCT	CAA	GAA	TTT	TAC	CAA	AAC	ACA	CTA	AGC	TTC	CTC	AAG	TCC	AAT	GGG	GAT	CTG	TGC	TAT	GGG	GCA	TTG	GCT	GCC	ATC	[1080]
	T	I	V	Q	G	A	L	K	S	I	L	R	R	T	P	Q	E	F	Y	Q	N	T	L	S	F	L	K	S	N	G	D	L	C	Y	G	A	L	A	A	I	[360]
Leptonycteris yerbabuena	ACC	ATT	GTC	CAG	GGT	GCT	TTG	AAA	AGC	ATC	CTG	CGT	CGC	ACC	CCT	CAA	GAG	TTC	TAC	CAC	AAC	ACT	CTA	AGC	TTC	CTC	AAG	TCC	AAT	GCT	GAT	CTG	TGC	TAT	GGG	GCT	TTG	GCT	GCC	ATC	[1080]
	T	I	V	Q	G	A	L	K	S	I	L	R	R	T	P	Q	E	F	Y	H	N	T	L	S	F	L	K	S	N	A	D	L	C	Y	G	A	L	A	A	I	[360]
Artibeus lituratus	ACC	ATT	GTC	CAG	GGA	GCT	TTG	AAA	AGC	ATC	CTG	CGT	CGC	ACC	CCT	CAA	GAG	TTC	TAC	CAC	AAC	ACT	CTA	AGC	TTC	CTC	AAG	TCC	AAT	GCT	GAC	TTG	TGC	TAT	GGG	GCT	TTG	GCT	GCC	ATT	[1080]
	T	I	V	Q	G	A	L	K	S	I	L	R	R	T	P	Q	E	F	Y	H	N	T	L	S	F	L	K	S	N	A	D	L	C	Y	G	A	L	A	A	I	[360]

	R395Q V397FA398V	
human	CCT GGA CTC CGG CCA GTC CGC CCT TCT GGG GCT ATG TAC CTC ATG GTT GGA ATT GAG ATG GAA CAT TTC CCA GAA TTT GAG AAC GAT GTG GAG TTC ACG GAG CGG TTA GTT GCT GAG CAG	[1200]
	P G L R P V R P S G A M Y L M V G I E M E H F P E F E N D V E F T E R L V A E Q	[400]
mouse	CCT GGA CTC CAG CCA GTC CGC CCA TCT GGA GCC ATG TAC CTT ATG GTG GGA ATT GAG ATG GAG CAC TTC CCA GAA TTT GAG AAT GAC GTG GAA TTC ACA GAG CGG TTA ATT GCG GAG CAG	[1200]
	P G L Q P V R P S G A M Y L M V G I E M E H F P E F E N D V E F T E R L I A E Q	[400]
rat	CCT GGA CTC CAG CCG GTC CGC CCT TCT GGA GCC ATG TAC CTT ATG GTG GGA ATT GAG ATG GAG CAT TTC CCG GAA TTC GAG AAC GAC GTG GAG TTC ACA GAG CGG TTG ATT GCG GAG CAG	[1200]
	P G L Q P V R P S G A M Y L M V G I E M E H F P E F E N D V E F T E R L I A E Q	[400]
cow	CCT GGA CTC CGG CCC ATT CGC CCT TCT GGG GCC ATG TAC CTC ATG GTT GGA ATT GAG ATG GAA CAT TTC CCA GAA TTT GAG AAT GAT GTG GAG TTC ACG GAG CAG TTA GTT GCT GAG CAA	[1200]
	P G L R P I R P S G A M Y L M V G I E M E H F P E F E N D V E F T E Q L V A E Q	[400]
dog	CCT GGA CTC CGG CCA GTC CGC CCT TCC GGG GCC ATG TAC CTC ATG GTT GGA ATT GAG ATG GAA CAT TTC CCA GAA TTT GAG AAT GAT GTG GAG TTC ACA GAG CGG TTA GTT GCT GAA CAA	[1200]
	P G L R P V R P S G A M Y L M V G I E M E H F P E F E N D V E F T E R L V A E Q	[400]
pig	CCT GGT CTC CGG CCC ATC CGT CCT TGT GGG GCC ATG TAT CTC ATG GTT GGA ATT GAG ATG GAA CAG TTC CCA GAA TTT GAG AAT GAC GTG GAG TTC ACA GAG CGG TTG GTT GCT GAG CAA	[1200]
	P G L R P I R P C G A M Y L M V G I E M E Q F P E F E N D V E F T E R L V A E Q	[400]
panda	CCT GGA CTC CAG CCA GTC CGT CCT TCC GGG GCC ATG TAC CTC ATG GTT GGA ATT GAG ATG GAA CAT TTC CCA GAA TTT GAG AAT GAC GTG GAG TTC ACG GAG CGC TTA GTT GCT GAG CAG	[1200]
	P G L Q P V R P S G A M Y L M V G I E M E H F P E F E N D V E F T E R L V A E Q	[400]
horse	CCT GGA CTC CGG CCA GTC CGC CCT TCT GGG GCC ATG TAC CTC ATG GTT GGA ATT GAG ATG GAA CAT TTC CCA GAA TTT GAG AAT GAT GTG GAG TTC ACA GAA CGC TTA GTT GCT GAG CAA	[1200]
	P G L R P V R P S G A M Y L M V G I E M E H F P E F E N D V E F T E R L V A E Q	[400]
Cynopterus sphinx	CCT GGA CTC CGA CCG GTC CGC CCT TCT GGG GCC ATG TAC CTT ATG GTT GAA ATT GAG ATG GAA CAT TTC CCA GAA TTT GAG AAT GAT GTG GAG TTC ACC GAG CGG TTA TTT GTT GAG CAA	[1200]
	P G L R P V R P S G A M Y L M V E I E M E H F P E F E N D V E F T E R L F V E Q	[400]
Rousettus leschenaultii	CCT GGA CTC CGG CCA GTC CGC TCT TCT GGG GCC ATG TAC ATT ATG GTT GAA ATT CAG ATG GAA CAT TTC CCA GAA TTT GAG AAT GAC GTG GAG TTC ACG GAG CAG TTA TTT GTT GAG CAA	[1200]
	P G L R P V R S S G A M Y I M V E I Q M E H F P E F E N D V E F T E Q L F V E Q	[400]
Eonycteris spelaea	CCT GGA CTC CGG CCA GTC CAC TCT TCT GGG GCC ATG TAC CTT ATG GTT GAA ATT GAG ATG GAA CAT TTC CCA GAA TTT GAA AAT GAT GTG GAG TTC ACG GAG CAG TTA TTT GTT GAG CAA	[1200]
	P G L R P V H S S G A M Y L M V E I E M E H F P E F E N D V E F T E Q L F V E Q	[400]
Pteropus vampyrus	CCT GGA CTC CGG CCG GTC CGC CCT TCT GGG GCC ATG TAC CTT ATG GTT GAA ATT GAG ATG GAA CAT TTC CCA GAA TTT GAG AAT GAT GTG GAG TTC ACG GAG CAG TTA TTT GTT GAG CAA	[1200]
	P G L R P V R P S G A M Y L M V E I E M E H F P E F E N D V E F T E Q L F V E Q	[400]
Rhinolophus ferrumequinum	CCT GGA CTC CGG CCA GTC CGC CCG TCT GGG GCC ATG TAC CTC ATG GTT GAA ATT GAG ATG GAA CAT TTC CCA GAA TTT GAG AAT GAT GTG GAG TTC ACG GAA CGG TTA GTT GCC GAG CAA	[1200]
	P G L R P V R P S G A M Y L M V E I E M E H F P E F E N D V E F T E R L V A E Q	[400]
Rhinolophus pusillus	CCT GGA CTC CGG CCA GTC CGC CCG TCT GGG GCC ATG TAC CTC ATG GTT GAA ATT GAG ATG GAA CAT TTC CCA GAA TTT AAG AAT GAT GTG GAG TTC ACG GAG CGG TTA GTT GCC GAG CAA	[1200]
	P G L R P V R P S G A M Y L M V E I E M E H F P E F K N D V E F T E R L V A E Q	[400]
Hipposideros armiger	CCT GGA CTC CGG CCA GTC CGT CCT TCT GGG GCC ATG TAC CTC ATG GTT GAA ATT GAG ATG GAA CAT TTC CCA GAA TTT GAG AAT GAT GTG GAG TTC ACG GAG CGG TTA GTT GCT GAG CAA	[1200]
	P G L R P V R P S G A M Y L M V E I E M E H F P E F E N D V E F T E R L V A E Q	[400]
Hipposideros pratti	CCT GGA CTC CGG CCA GTC CGT CCT TCT GGG GCC ATG TAC CTC ATG GTT GAA ATT GAG ATG GAA CAT TTC CCA GAA TTT GAG AAT GAT GTG GAG TTC ACG GAG CGG TTA GTT GCT GAG CAA	[1200]
	P G L R P V R P S G A M Y L M V E I E M E H F P E F E N D V E F T E R L V A E Q	[400]
Megaderma lyra	CCT GGA CTC CGG CCA GTT CGC CCT TCT GGG GCT ATG TAC CTC ATG GTT GAA ATT GAG ATG GAA CAT TTC CCA GAA TTT AAG GAT GAC GTG GAG TTC ACG GAG CGG TTA GTT GCC GAG CAG	[1200]
	P G L R P V R P S G A M Y L M V E I E M E H F P E F K D D V E F T E R L V A E Q	[400]
Megaderma spasma	CCT GGA CTC CGG CCA GTT CGC CCT TCT GGG GCT ATG TAC CTC ATG GTT GAA ATT GAG ATG GAA CAT TTC CCA GAA TTT AAG GAT GAT GTG GAG TTC ACG GAG CGG TTA GTT GCC GAG CAG	[1200]
	P G L R P V R P S G A M Y L M V E I E M E H F P E F K D D V E F T E R L V A E Q	[400]
Myotis ricketti	CCT GGA CTG CGG CCA GTC CGC CCT TCT GGG GCC ATG TAC CTC ATG GTT GAA ATT GAG ATG GAA CAT TTC CCA GAA TTT GAG AAT GAT GTG GAG TTC ACG GAG CGG TTA GTT GCT GAA CAA	[1200]
	P G L R P V R P S G A M Y L M V E I E M E H F P E F E N D V E F T E R L V A E Q	[400]
Pipistrellus abramus	CCT GGA CTC CGG CCA GTC CGC CCT TCT GGG GCC ATG TAC CTC ATG GTT GAA ATT GAG ATG GAA CAT TTC CCA GAA TTT GAG AAT GAC GTG GAG TTC ACG GAG CGG TTA ATT GCT GAA CAA	[1200]
	P G L R P V R P S G A M Y L M V E I E M E H F P E F E N D V E F T E R L I A E Q	[400]
Scotophilus kuhlii	CCT GGG CTC CGG CCA GTC CGC CCT TCT GGG GCC ATG TAC CTC ATG GTT GAA ATT GAG ATG GAA CAT TTC CCA GAA TTT GAG AAT GAT GTG GAG TTC ACG GAG CGG TTA GTT GCT GAA CAG	[1200]
	P G L R P V R P S G A M Y L M V E I E M E H F P E F E N D V E F T E R L V A E Q	[400]
Miniopterus fuliginosus	CCT GGA CTC CGG CCA GTC CGC CCT TCT GGG GCC ATG TAC CTC ATG GTT GAA ATT GAG ATG GAA CAT TTC CCA GAA TTT GAG AAT GAT GTG GAG TTC ACG GAG CGG TTA GTT GCT GAA CAA	[1200]
	P G L R P V R P S G A M Y L M V E I E M E H F P E F E N D V E F T E R L V A E Q	[400]
Tadarida plicata	CCT GGA CTC CGA CCA GTC CGC CCT TCT GGG GCT ATG TAC CTC ATG GTT GAA ATT GAG ATG GAA CAT TTC CCA GAA TTT GAG AAT GAT GTG GAT TTC ACG GAG CGG TTA GTT GCT GAA CAA	[1200]
	P G L R P V R P S G A M Y L M V E I E M E H F P E F E N D V D F T E R L V A E Q	[400]
Mormoops megalophylla	CCT GGA CTC CGG CCA ATC CGC CCT CTG GGG GCC ATG TAC CTC ATG GTT GAA ATT GAG ATG GAG CAT TTC CCA GAA TTT GAG AAC GAT GAG GAG TTC ACG GAG CGG TTA ATT GCC GAA CAG	[1200]
	P G L R P I R P L G A M Y L M V E I E M E H F P E F E N D E E F T E R L I A E Q	[400]
Pteronotus parnellii	CCT GGA CTC CGG CCA GTC CGC CCT TCG GGG GCC ATG TAC CTC ATG GTT GAA ATT CAG ATG GAG CAT TTC CCA GAG TTT GAG AAC GAT GTG GAG TTC ACA GAG CGG TTA GTT GCT GAA CAA	[1200]
	P G L R P V R P S G A M Y L M V E I Q M E H F P E F E N D V E F T E R L V A E Q	[400]
Desmodus rotundus	CCT GGA CTC CGG CCA GTC CGC CCT TCG GGA GCC ATG TAC CTC ATG GTT GAA ATT GAG ATG GAG CAT TTC CCA GAA TTT GGG AAC GAT GTG GAG TTC ACA GAG CGG TTA GTT GCT GAA CAG	[1200]
	P G L R P V R P S G A M Y L M V E I E M E H F P E F G N D V E F T E R L V A E Q	[400]
Leptonycteris yerbabuena	CCT GGA CTC CGG CCA GTC CGC CCT TCG GGG ACC ATG TAC CTC ATG GTT GAA ATT GAG ATG GAG CAT TTC CCA GAA TTT GAG AAT GAT GTG GAG TTC ACG GAG CGG TTA GTT GCT GAA CAG	[1200]
	P G L R P V R P S G T M Y L M V E I E M E H F P E F E N D V E F T E R L V A E Q	[400]
Artibeus lituratus	CCT GGA CTC TGG CCA GTC CGC CCT TCG GGG GCC ATG TAC CTC ATG GTT AAA ATT GAA ATG GAG CAT TTC CCA GAA TTT GAG AAT GAT GTG GAA TTC ACA GAG CGG TTA GTT GCT GAA CAG	[1200]
	P G L W P V R P S G A M Y L M V K I E M E H F P E F E N D V E F T E R L V A E Q	[400]
