# Supplementary figures and images for: Relaxed Evolution in the Tyrosine Aminotransferase Gene Tat in Old World Fruit Bats (Chiroptera: Pteropodidae)
Source: PLoS One. 2014 May 13;9(5):e97483. doi: 10.1371/journal.pone.0097483 (PMC4019583; doi:10.1371/journal.pone.0097483)

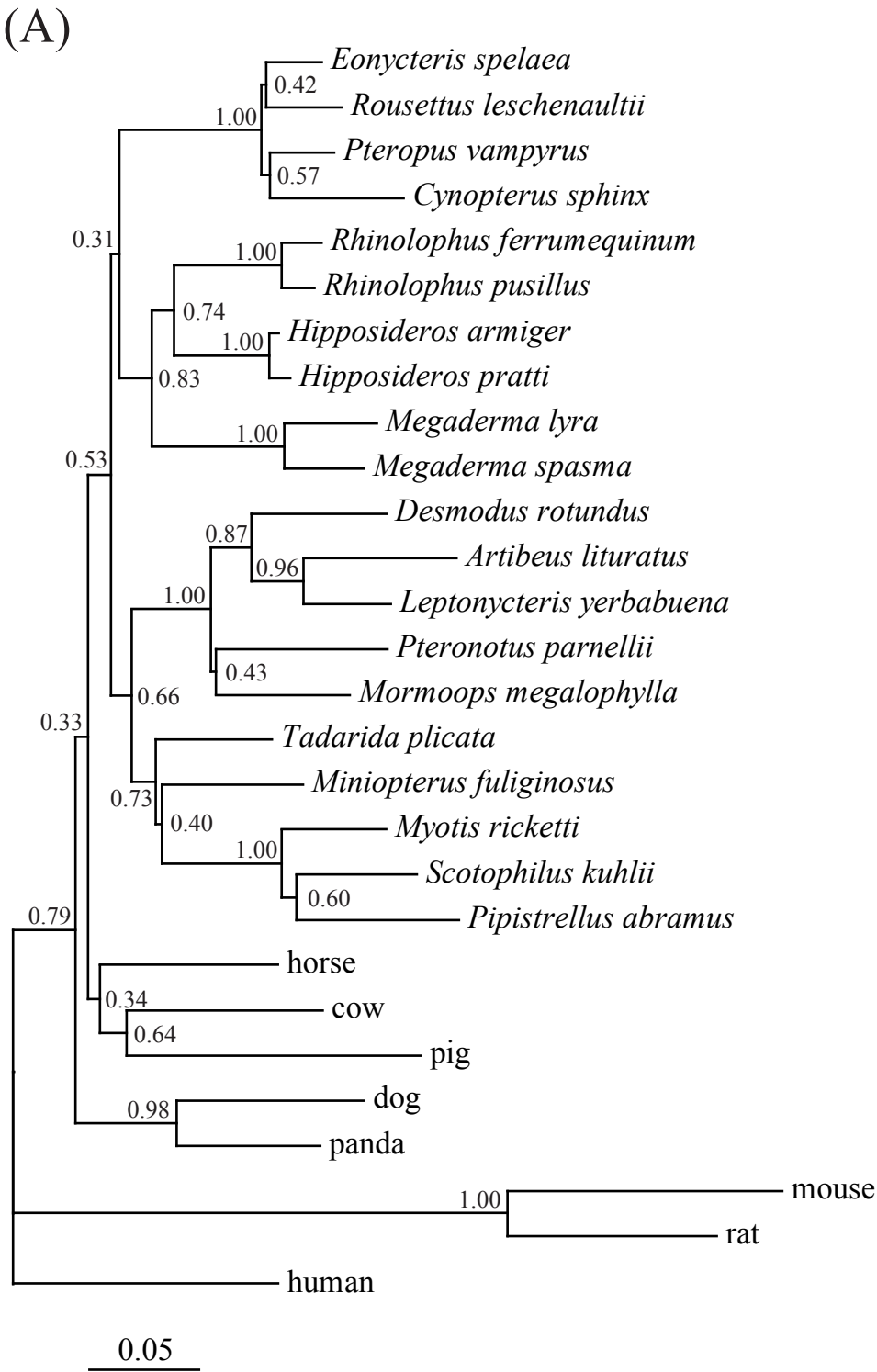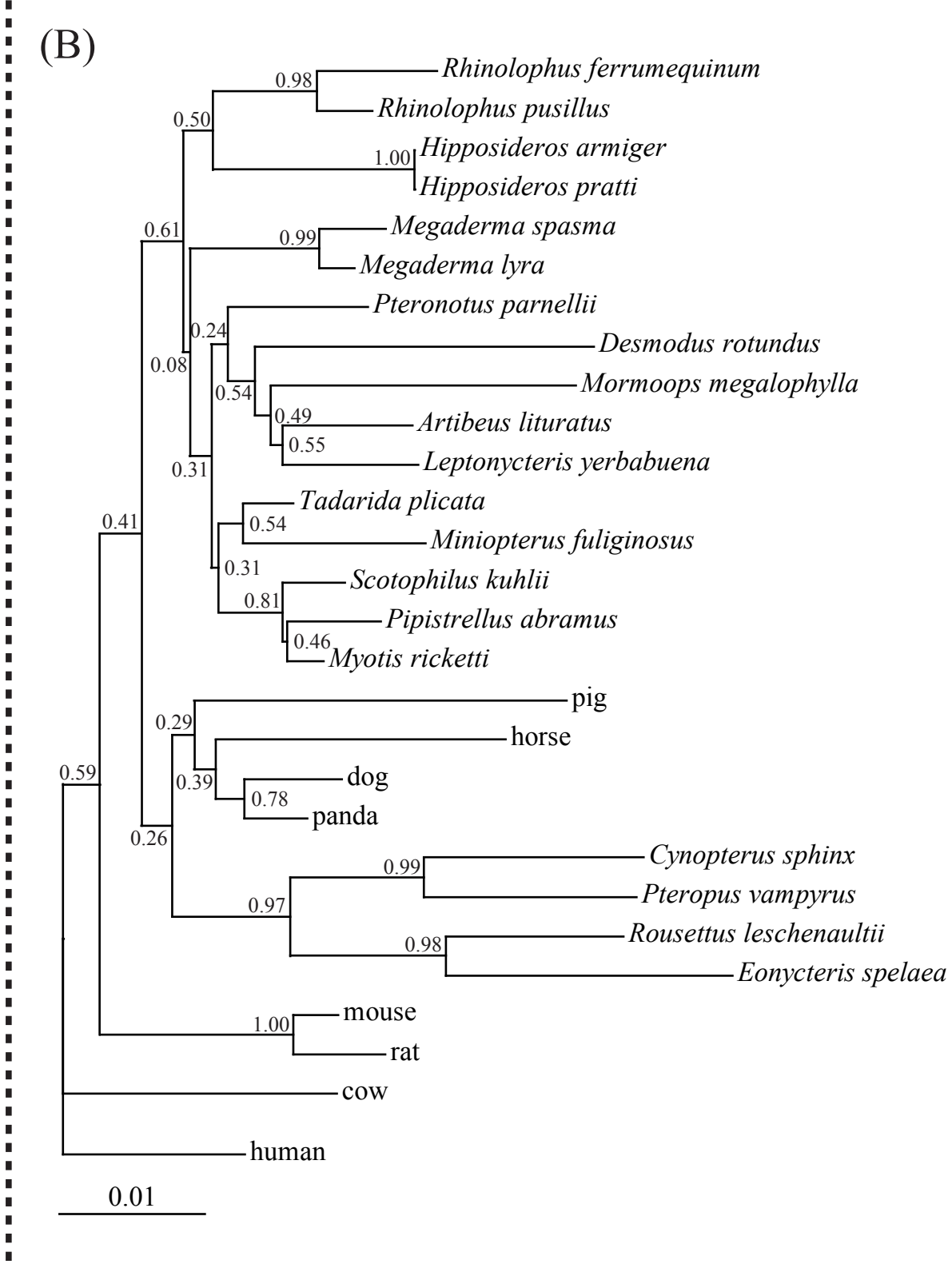

Supplement: Figure S5 — Neighbor-Joining (NJ) trees based on synonymous and nonsynonymous substitutions using the Tat gene nucleotide dataset removing codons for 21 shared amino acid changes in the Old World fruit bats, under the Kumar model. (A) NJ tree based on synonymous substitutions. (B) NJ tree based on nonsynonymous substitutions. Codons corresponding to amino acids at positions 39, 51, 53, 65, 66, 83, 93, 94, 97, 164, 171, 232, 258, 263, 268, 307, 317, 355, 395, 397 and 398 are removed prior to phylogenetic reconstruction analyses. Values on the nodes are Neighbor-Joining bootstrap values. Branch lengths are based on the number of synonymous substitutions per synonymous site for (A) and the number of nonsynonymous substitutions per nonsynonymous site for (B). (PDF) [file pone.0097483.s005.pdf]

(A)

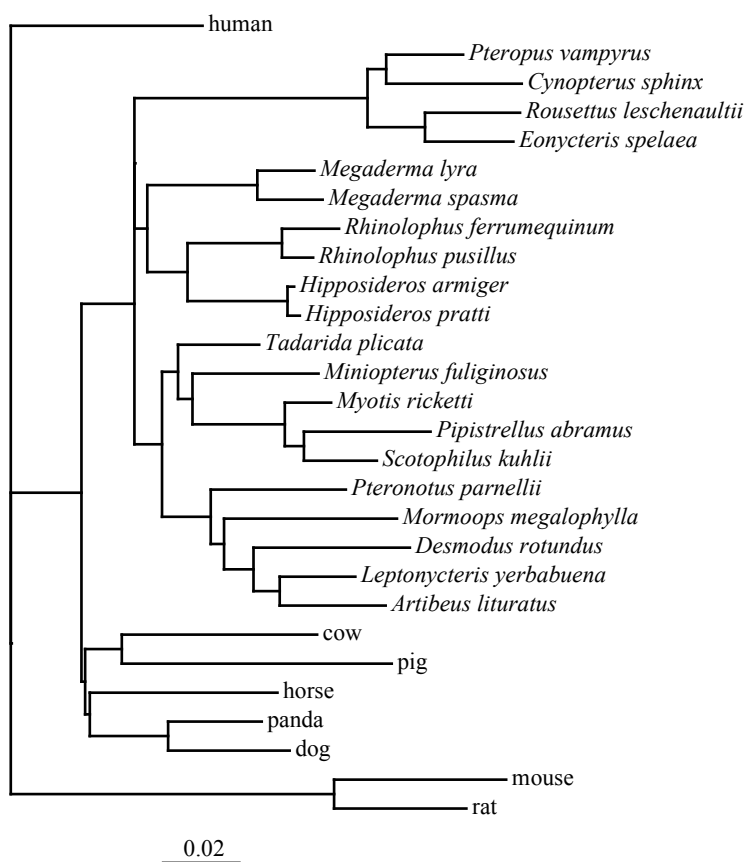

(B)

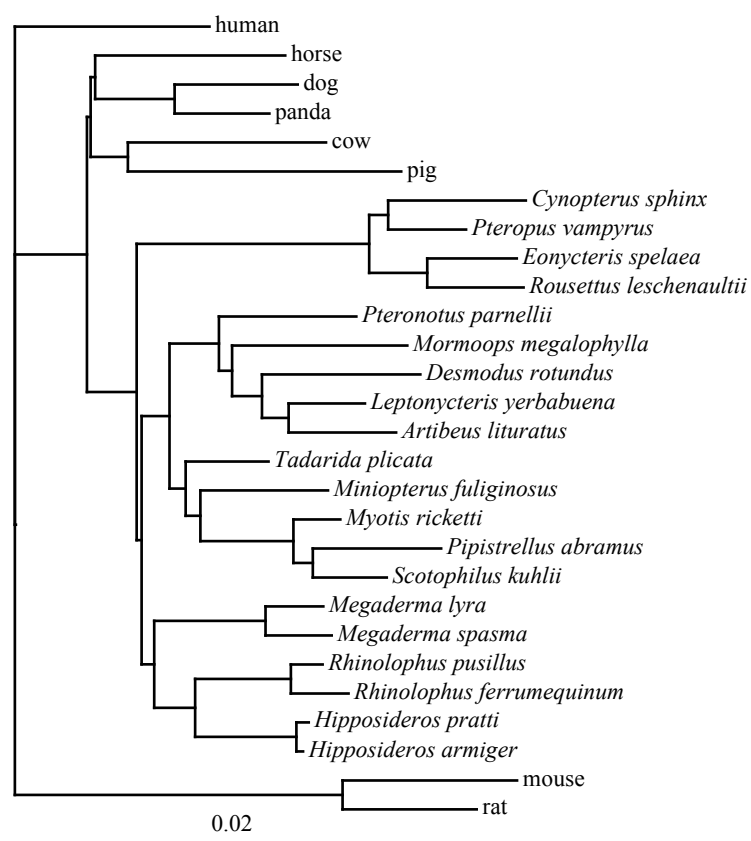

(C)

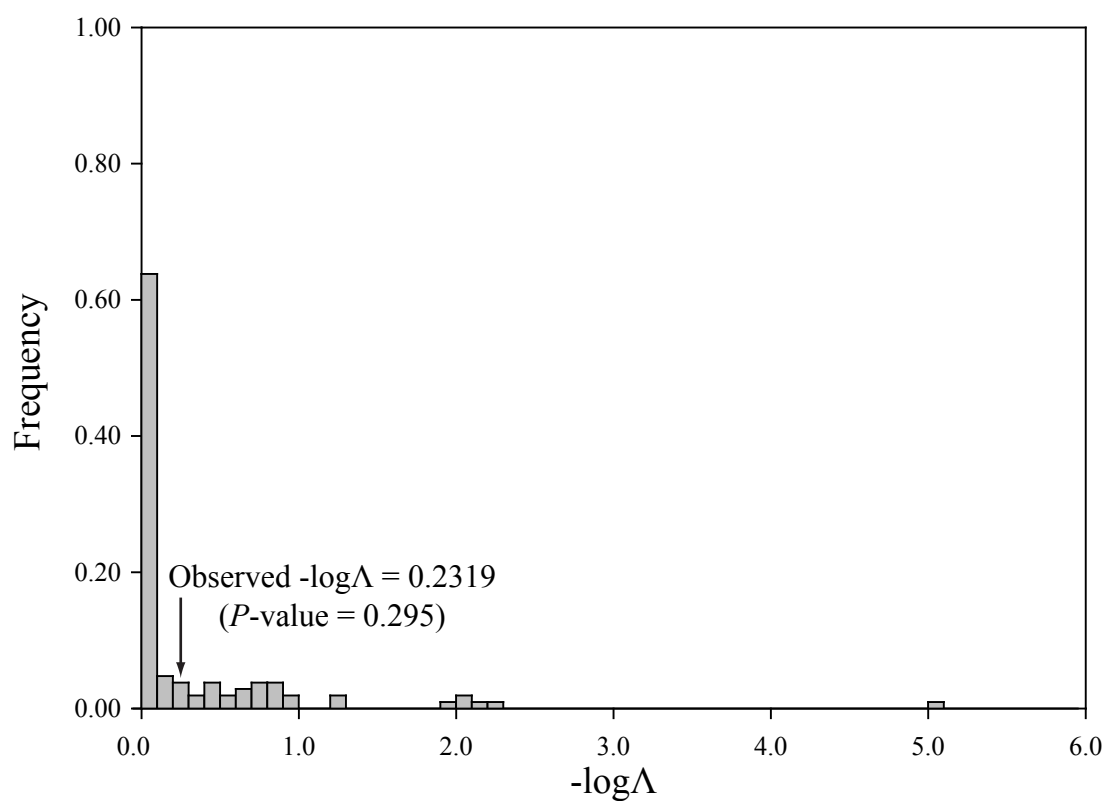

Supplement: Figure S6 — Results of the parametric bootstrapping analysis. (A) Constrained species tree based on the Tat gene coding sequences as the null hypothesis (log L = −8528.75). Two hundred simulated datasets were generated based on this tree topology. (B) Unconstrained maximum-likelihood tree based on the Tat gene coding sequences as the alternative hypothesis (log L = −8528.52). (C) Distribution of the differences in maximum likelihood values under the assumption that the null hypothesis (constrained species tree) is correct. The observed difference of maximum likelihood values (–log Λ = 0.2319) is not significant (P-value = 0.295), thus, the constrained species tree could not be rejected by the unconstrained maximum-likelihood tree. (PDF) [file pone.0097483.s006.pdf]

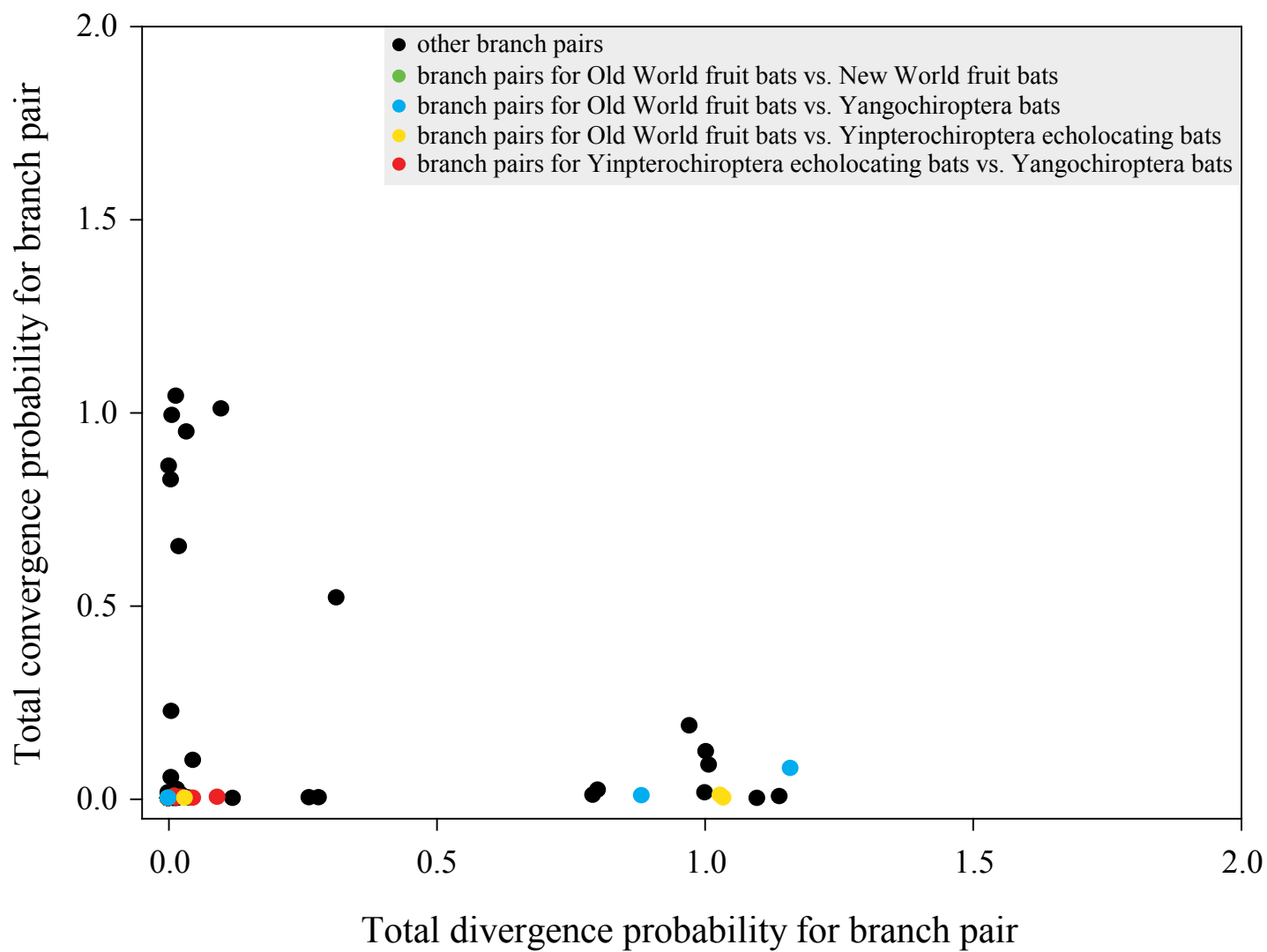

Supplement: Figure S7 — Plot of total posterior probabilities of divergence versus convergence for all pairs of branches in the tree. Pairwise comparison for the branches of Yinpterochiroptera echolocating bats versus branches of the Yangochiroptera bats, the ancestral branch of the Old World fruit bats versus the branches of Yinpterochiroptera echolocating bats, the ancestral branch of the Old World fruit bats versus branches of Yangochiroptera bats and the ancestral branch of the Old World fruit bats versus the ancestral branch of the New World fruit bats are highlighted. (PDF) [file pone.0097483.s007.pdf]
