# Supplementary material for: Relaxed Evolution in the Tyrosine Aminotransferase Gene Tat in Old World Fruit Bats (Chiroptera: Pteropodidae)
Source: PLoS One. 2014 May 13;9(5):e97483. doi: 10.1371/journal.pone.0097483 (PMC4019583; doi:10.1371/journal.pone.0097483)
Supplement: Table S1 — Species analyzed in this study. (DOC) [file pone.0097483.s008.doc]

**Table S1. Species analyzed in this study**

| **Species Name** | **Family** | **Accession Number** |
| --- | --- | --- |
| *Cynopterus sphinx* | Pteropodidae | KJ161834 |
| *Rousettus leschenaultii* | Pteropodidae | KJ161849 |
| *Eonycteris spelaea* | Pteropodidae | KJ161836 |
| *Pteropus vampyrus* | Pteropodidae | Ensemble database |
| *Rhinolophus ferrumequinum* | Rhinolophidae | KJ161847 |
| *Rhinolophus pusillus* | Rhinolophidae | KJ161848 |
| *Hipposideros armiger* | Hipposideridae | KJ161837 |
| *Hipposideros pratti* | Hipposideridae | KJ161838 |
| *Megaderma spasma* | Megadermatidae | KJ161841 |
| *Megaderma lyra* | Megadermatidae | KJ161840 |
| *Mormoops megalophylla* | Mormoopidae | KJ161843 |
| *Pteronotus parnellii* | Mormoopidae | KJ161846 |
| *Desmodus rotundus* | Phyllostomidae | KJ161835 |
| *Artibeus lituratus* | Phyllostomidae | KJ161833 |
| *Leptonycteris yerbabuenae* | Phyllostomidae | KJ161839 |
| *Myotis ricketti* | Vespertilionidae | KJ161844 |
| *Scotophilus kuhlii* | Vespertilionidae | KJ161850 |
| *Pipistrellus abramus* | Vespertilionidae | KJ161845 |
| *Miniopterus fuliginosus* | Miniopteridae | KJ161842 |
| *Tadarida plicata* | Molossidae | KJ161851 |
| human | Hominidae | NM_000353 |
| mouse | Muridae | NM_146214 |
| rat | Muridae | NM_012668 |
| dog | Canidae | XM_536796 |
| giant panda | Ursidae | XM_002920948 |
| cow | Bovidae | XM_005218654 |
| pig | Suidae | XM_003126884 |
| horse | Equidae | XM_001498000 |
