# Supplementary material for: Relaxed Evolution in the Tyrosine Aminotransferase Gene Tat in Old World Fruit Bats (Chiroptera: Pteropodidae)
Source: PLoS One. 2014 May 13;9(5):e97483. doi: 10.1371/journal.pone.0097483 (PMC4019583; doi:10.1371/journal.pone.0097483)
Supplement: Table S2 — Primers used for amplifying Tat coding sequences by PCR. (DOC) [file pone.0097483.s009.doc]

**Table S2. Primers used for amplifying *Tat* coding sequences by PCR**

| **Species name** | **Primer pair for PCR** | **Tm(°C)^a^** |
| --- | --- | --- |
| *Cynopterus sphinx*  *Rousettus leschenaultii*  *Eonycteris spelaea*  *Hipposideros armiger*  *Megaderma lyra*  *Mormoops megalophylla*  *Desmodus rotundus*  *Artibeus lituratus*  *Leptonycteris yerbabuenae*  *Myotis ricketti*  *Tadarida plicata* | Forward primer: F1: 5’-CACAGACTTCACTAGTGATGGAC-3’  Reverse primer: R1: 5’-GAGCATAGACTCAGGCCTATTTG-3’ | 60 |
| *Rhinolophus ferrumequinum*  *Rhinolophus pusillus*  *Hipposideros pratti*  *Megaderma spasma*  *Pteronotus parnellii*  *Scotophilus kuhlii*  *Pipistrellus abramus*  *Miniopterus fuliginosus* | Forward primer: F2: 5’-CACAGACTTTGCTAGTGATGGACC-3’  Reverse primer: R2: 5’-GAGCATGGGCTCAGGCCTATTTG-3’ | 60 |

^a^Tm, annealing temperature.
