# Supplementary material for: Relaxed Evolution in the Tyrosine Aminotransferase Gene Tat in Old World Fruit Bats (Chiroptera: Pteropodidae)
Source: PLoS One. 2014 May 13;9(5):e97483. doi: 10.1371/journal.pone.0097483 (PMC4019583; doi:10.1371/journal.pone.0097483)
Supplement: Table S3 — Results of pairwise relative rate tests. (DOC) [file pone.0097483.s010.doc]

**Table S3. Results of pairwise relative rate tests**

| **Outgroups^a^** | **Ingroup 1^b^**  **(Old World fruit bats)** | **Ingroup 2^c^**  **(Insectivorous bats)** | **Synrate tests^d^** | | **Nonsynrate tests^d^** | |
| --- | --- | --- | --- | --- | --- | --- |
|  |  |  | **LRT^e^** | ***P*-value** | **LRT^e^** | ***P*-value** |
| Human | *Cynopterus sphinx* | *Rhinolophus ferrumequinum* | 0.166 | 0.684 | 19.247 | <0.001 |
|  |  | *Megaderma lyra* | 0.003 | 0.959 | 34.909 | <0.001 |
|  |  | *Mormoops megalophylla* | 0.010 | 0.920 | 11.758 | <0.001 |
|  |  | *Myotis ricketti* | 0.043 | 0.835 | 33.488 | <0.001 |
|  | *Rousettus leschenaultii* | *Rhinolophus ferrumequinum* | 0.571 | 0.449 | 14.056 | <0.001 |
|  |  | *Megaderma lyra* | 1.309 | 0.252 | 25.866 | <0.001 |
|  |  | *Mormoops megalophylla* | 1.445 | 0.229 | 8.068 | <0.01 |
|  |  | *Myotis ricketti* | 1.609 | 0.205 | 22.376 | <0.001 |
|  | *Eonycteris spelaea* | *Rhinolophus ferrumequinum* | 1.779 | 0.182 | 21.515 | <0.001 |
|  |  | *Megaderma lyra* | 2.836 | 0.092 | 35.711 | <0.001 |
|  |  | *Mormoops megalophylla* | 3.262 | 0.071 | 13.903 | <0.001 |
|  |  | *Myotis ricketti* | 3.384 | 0.066 | 31.469 | <0.001 |
|  | *Pteropus vampyrus* | *Rhinolophus ferrumequinum* | 0.767 | 0.381 | 17.834 | <0.001 |
|  |  | *Megaderma lyra* | 1.565 | 0.211 | 31.993 | <0.001 |
|  |  | *Mormoops megalophylla* | 1.788 | 0.181 | 11.071 | <0.001 |
|  |  | *Myotis ricketti* | 2.193 | 0.139 | 28.229 | <0.001 |
| Cow | *Cynopterus sphinx* | *Rhinolophus ferrumequinum* | 0.966 | 0.326 | 16.028 | <0.001 |
|  |  | *Megaderma lyra* | 0.261 | 0.609 | 28.036 | <0.001 |
|  |  | *Mormoops megalophylla* | 0.168 | 0.682 | 9.964 | <0.01 |
|  |  | *Myotis ricketti* | 0.110 | 0.739 | 34.158 | <0.001 |
|  | *Rousettus leschenaultii* | *Rhinolophus ferrumequinum* | 0.341 | 0.559 | 14.219 | <0.001 |
|  |  | *Megaderma lyra* | 0.031 | 0.861 | 24.483 | <0.001 |
|  |  | *Mormoops megalophylla* | 0.008 | 0.931 | 8.669 | <0.01 |
|  |  | *Myotis ricketti* | 0.407 | 0.524 | 26.673 | <0.001 |
|  | *Eonycteris spelaea* | *Rhinolophus ferrumequinum* | 0.051 | 0.822 | 14.093 | <0.001 |
|  |  | *Megaderma lyra* | 0.401 | 0.527 | 23.416 | <0.001 |
|  |  | *Mormoops megalophylla* | 0.577 | 0.447 | 8.775 | <0.01 |
|  |  | *Myotis ricketti* | 2.182 | 0.139 | 25.523 | <0.001 |
|  | *Pteropus vampyrus* | *Rhinolophus ferrumequinum* | 0.063 | 0.802 | 16.546 | <0.001 |
|  |  | *Megaderma lyra* | 0.449 | 0.503 | 28.719 | <0.001 |
|  |  | *Mormoops megalophylla* | 0.647 | 0.421 | 10.885 | <0.001 |
|  |  | *Myotis ricketti* | 2.625 | 0.105 | 31.852 | <0.001 |

^a^Mammal species (human, mouse, rat, horse, cow, pig, panda and dog) are used as outgroup separately for relative rate tests, only results using human and cow as outgroup are shown in the table.

^b^Four Old World fruit bats are used as ingroup 1 to separately compare with insectivorous bats.

^c^Insectivorous bats are used separately as ingroup 2 for relative rate tests to compare with the Old World fruit bats, only results of four selected insectivorous bats are shown in the table.

^d^Synrate tests, Synonymous rate tests. Nonsynrate tests, Nonsynonymous rate tests.

^e^LRT, Likelihood Ratio Test.
