# Supplementary material for: Relaxed Evolution in the Tyrosine Aminotransferase Gene Tat in Old World Fruit Bats (Chiroptera: Pteropodidae)
Source: PLoS One. 2014 May 13;9(5):e97483. doi: 10.1371/journal.pone.0097483 (PMC4019583; doi:10.1371/journal.pone.0097483)
Supplement: Methods S1 — (DOC) [file pone.0097483.s011.doc]

**Supplementary Information**

**Supplementary Methods**

All of our three phylogenetic reconstruction methods recovered a gene tree where all of the laryngeal echolocating bats (including New World fruit bats) formed a monophyletic group, thus in conflict with the accepted species topology. Such phylogenetic conflicts with a species tree might be caused by convergent evolution of the *Tat* gene in insectivorous bats, in relation to the relatively high-protein content insect diets of these species. To test for explicit evidence for convergence between yinpterochiropteran and yangochiropteran insectivorous bats, we conducted the methods described by Castoe et al. (2009) [1] implemented in the codeMLancestral package. In this approach, site-wise posterior probabilities of convergent and divergent amino acid substitutions are estimated and summed under a JTT amino acid substitution model for each pair of independent branches in the established species tree [2,3,4], whose branch lengths were estimated using MrBayes 3.1.2 [5]. We compared the branches of the yinpterochiropteran insectivorous bats versus the branches of the yangochiropteran insectivorous bats and the other branches.

In addition to the approximately unbiased test, we also conducted a parametric bootstrapping analysis to test whether the recovered *Tat* gene tree is significantly different from the species tree. For this purpose, we first reconstructed the optimal gene tree (alternative hypothesis) and the constrained species tree (null hypothesis) based on the original *Tat* sequence dataset under the GTR+Γ nucleotide substitution model using RaxML v7.0.4 [6], to obtain the observed difference in maximum likelihood values (-log Λ) between the gene tree and the constrained species tree. Then we simulated 200 datasets using Seq-Gen v1.3.3 [7] based on the GTR model, parameters estimated (Alpha = 0.4560; A to C = 1.4988, A to G = 6.0273, A to T =0.9682, C to G = 0.8468, C to T = 6.8956, G to T = 1.0000; frequencies of A, C, G and T are 0.2385, 0.2536, 0.2681 and 0.2399, respectively) and the constrained species tree topology with branch length estimated by RaxML v7.0.4. We repeated the phylogenetic reconstructions of the optimal tree and the constrained species tree using these 200 simulated datasets to obtain the null distribution of the difference of maximum likelihood values. Finally, we compared the observed difference from the original data against the null distribution of differences based on the simulations.

**Supplemental references**

1. Castoe TA, de Koning APJ, Kim HM, Gu W, Noonan BP, et al. (2009) Evidence for an ancient adaptive episode of convergent molecular evolution. Proc Natl Acad Sci U S A 106: 8986-8991.

2. Zhou X, Xu S, Xu J, Chen B, Zhou K, et al. (2012) Phylogenomic analysis resolves the interordinal relationships and rapid diversification of the Laurasiatherian mammals. Syst Biol 61: 150-164.

3. Datzmann T, von Helversen O, Mayer F (2010) Evolution of nectarivory in phyllostomid bats (Phyllostomidae Gray, 1825, Chiroptera: Mammalia). BMC Evol Biol 10: 165.

4. Teeling EC, Springer MS, Madsen O, Bates P, O'Brien SJ, et al. (2005) A molecular phylogeny for bats illuminates biogeography and the fossil record. Science 307: 580-584.

5. Ronquist F, Huelsenbeck JP (2003) MrBayes 3: Bayesian phylogenetic inference under mixed models. Bioinformatics 19: 1572-1574.

6. Stamatakis A (2006) RAxML-VI-HPC: maximum likelihood-based phylogenetic analyses with thousands of taxa and mixed models. Bioinformatics 22: 2688-2690.

7. Rambaut A, Grassly NC (1997) Seq-Gen: an application for the Monte Carlo simulation of DNA sequence evolution along phylogenetic trees. Comput Appl Biosci 13: 235-238.

**Supplementary Figure Legends**

**Figure S1. Schematic of *Tat* gene exons.** (A) A cartoon illustrating the primer pair designations for the *Tat* gene coding sequence amplification. Two pairs of primers are designed based on sequences spanning untranslated regions and exons. Primer sequences based on untranslated regions and exons are colored in black and red, respectively. Only exon 1 and exon 11 of the *Tat* gene are shown. (B) Genomic deletion of seven amino acids in four Old World fruit bats. Seven amino acids from positions 3 to 9 in the first exon of the *Tat* gene are deleted in the four Old World fruit bats. The human *Tat* gene sequence is used as reference.

**Figure S2. Alignment of the full amino acid sequences of the *Tat* gene from 28 mammals.** Twenty-one amino acid changes in the Old World fruit bats are highlighted by red and the changes are marked on the top of the alignment in blue. Seven amino acid mutations reported in humans with tyrosinemia type II are marked and indicated by asterisks (*), with mutation in red, on the top of the alignment. The first 23 amino acids of the amino-termini are not conserved and were removed from the dataset prior to phylogenetic reconstruction and molecular evolutionary analyses (see Materials and Methods) and are highlighted by the red box. Species belonging to the Old World fruit bats and the New World fruit bats are highlighted in green and blue, respectively.

**Figure S3. Variable amino acid sites in the sequences of the *Tat* gene from 28 mammals.** Variable sites from the alignment shown in Figure S1 are shown. Species belonging to the Old World fruit bats and the New World fruit bats are highlighted in green and blue, respectively.

**Figure S4. Alignment of the full nucleic acid sequences and corresponding amino acids of the *Tat* gene from 28 mammals.** Twenty-one amino acid changes in the Old World fruit bats are highlighted by red and the changes are marked on the top of the alignment in red. Species belonging to the Old World fruit bats and the New World fruit bats are highlighted in green and blue, respectively.

**Figure S5. Neighbor-Joining (NJ) trees based on synonymous and nonsynonymous substitutions using the *Tat* gene nucleotide dataset removing codons for 21 shared amino acid changes in the Old World fruit bats, under the Kumar model.** (A) NJ tree based on synonymous substitutions. (B) NJ tree based on nonsynonymous substitutions. Codons corresponding to amino acids at positions 39, 51, 53, 65, 66, 83, 93, 94, 97, 164, 171, 232, 258, 263, 268, 307, 317, 355, 395, 397 and 398 are removed prior to phylogenetic reconstruction analyses. Values on the nodes are Neighbor-Joining bootstrap values. Branch lengths are based on the number of synonymous substitutions per synonymous site for (A) and the number of nonsynonymous substitutions per nonsynonymous site for (B).

**Figure S6. Results of the parametric bootstrapping analysis.** (A) Constrained species tree based on the *Tat* gene coding sequences as the null hypothesis (log *L* = -8528.75). Two hundred simulated datasets were generated based on this tree topology. (B) Unconstrained maximum-likelihood tree based on the *Tat* gene coding sequences as the alternative hypothesis (log *L* = -8528.52). (C) Distribution of the differences in maximum likelihood values under the assumption that the null hypothesis (constrained species tree) is correct. The observed difference of maximum likelihood values (-log Λ = 0.2319) is not significant (*P*-value = 0.295), thus, the constrained species tree could not be rejected by the unconstrained maximum-likelihood tree.

**Figure S7. Plot of total posterior probabilities of divergence versus convergence for all pairs of branches in the tree.** Pairwise comparison for the branches of Yinpterochiroptera echolocating bats versus branches of the Yangochiroptera bats, the ancestral branch of the Old World fruit bats versus the branches of Yinpterochiroptera echolocating bats, the ancestral branch of the Old World fruit bats versus branches of Yangochiroptera bats and the ancestral branch of the Old World fruit bats versus the ancestral branch of the New World fruit bats are highlighted.

**Supplementary Tables**

Table S1. Species analyzed in this study

Table S2. Primers used for amplifying Tat coding sequences by PCR

Table S3. Results of pairwise relative rate tests
