# Supplementary material for: Relaxed Evolution in the Tyrosine Aminotransferase Gene Tat in Old World Fruit Bats (Chiroptera: Pteropodidae)
Source: PLoS One. 2014 May 13;9(5):e97483. doi: 10.1371/journal.pone.0097483 (PMC4019583; doi:10.1371/journal.pone.0097483)
Supplement: Figure S2 — Alignment of the full amino acid sequences of the Tat gene from 28 mammals. Twenty-one amino acid changes in the Old World fruit bats are highlighted by red and the changes are marked on the top of the alignment in blue. Seven amino acid mutations reported in humans with tyrosinemia type II are marked and indicated by asterisks (*), with mutation in red, on the top of the alignment. The first 23 amino acids of the amino-termini are not conserved and were removed from the dataset prior to phylogenetic reconstruction and molecular evolutionary analyses (see Materials and Methods) and are highlighted by the red box. Species belonging to the Old World fruit bats and the New World fruit bats are highlighted in green and blue, respectively. (PDF) [file pone.0097483.s002.pdf]

```
First 23 amino acids
removed from dataset
                                                                     A39P           F53Y K51R        V65M K66E              V83L        V93I T94I M97L                       R119W *
human                      MDPYMIQMSS KGNLPSILDV HVNVGGRSSV PGKMKGRKAR WSVRPSDMAK KTFNPIRAIV DNMKVKPNPN KTMISLSIGD PTVFGNLPTD PEVTQAMKDA LDSGKYNGYA PSIGFLSSRE [120]
mouse                      MDSYVIQTNV NDSLPSVLDV RVNIGGRSSV QGRAKGRKAR WNVRPSDMSN KTFNPIRAIV DNMKVKPNPN KTVISLSIGD PTVFGNLPTD PEVTQAMKDA LDSGKYNGYA PSIGYLSSRE [120]
rat                        MDSYVIQTDV DDSLSSVLDV HVNIGGRNSV QGRKKGRKAR WDVRPSDMSN KTFNPIRAIV DNMKVQPNPN KTVISLSIGD PTVFGNLPTD PEVTQAMKDA LDSGKYNGYA PSIGYLSSRE [120]
cow                        MDPYVIQMQD HGSLPSVLDV HVNVAGRSSV LGKVKSRKAR WSVRPSDMSN KTFNPIRAIV DNMKVKPNPN KTMIALSIGD PTVFGNLPTD PEVTQAMKDA LDSGKFNGYV PSIGYLSSRE [120]
dog                        MDPYVIEMNS NSNLPSVLDV HVNIGGRSSL PGKMKGRKAR WSVRPSDMSN KTFNPIRAIV DSSKVKPNPN KATIALSIGD PTVFGNLPTD PEVTQAMKDA LDSGKYNGYA PSIGYLSSRE [120]
pig                        MDPYVIQMHG HGNLPSVLDV HVNTAGRIPV PGKMKGRKAR WSVRPSDMSN KTCNPIRAIV DSMKVKPNPN KTTISLSIGD PTVFGNLPTD QEVTQAMKDA LDSGKYNGYA PSIGYLSSRE [120]
panda                      MDPYVIQMNS NSNLPSVLDV HVNIGGRSSL PGKMKGRKAR WSVKPSDMSN KTFNPIRAIV DSMKVKPNPN KTMIALSIGD PTVFGNLPTD PEVTQAMKDA LDSGKYNGYA PSIGYLSSRE [120]
horse                      MDPYVIQMTG SGNLPSVLDM HVNISGRSSA PGKMKGRKAR WSVRPSDMSN KTFNPIRAIV DNMKVKPNPN KTMIALSIGD PTVFGNLSTD PEVTQALKDA VDSGKYNGYA PTIGYLSSRE [120]
Cynopterus sphinx          MD-------S NGDLPSILGV HMHINGRSSA PEKVKGRKPR WSVKPSDMAN RTYNPIRAIV DSMKMEPNPD KTTITLSIGD PTLSGNLPTD PEIIQALKDA LDSGKYNGYA PSIGYLSSRE [120]
Rousettus leschenaultii    MD-------S DGDLPFLLDV HVSVNGRSSV PEKVKGRQPG WSVRPSDMAN RTYNPIRAIV DNMKLEPNPD KPMISLSLGD PTLFGNLPTD PEIIQALKDA LDSGKYNGYA PSIGYLSSRE [120]
Eonycteris spelaea         MD-------S SSDLPFLLDV HVSVSGRSPV PEKVKGRKTE WSVRPSDMAN RTYNPIRAIV DSMKMEPNPD KTTIALSIGD PTLFGNLPTD PEIIQALKDA LDSGKYNGYA PSIGYLSSRE [120]
Pteropus vampyrus          MD-------S KGDLPSVLDV HININGRSSV SEKVKGRKPR WSVRPSDMAN RTYNPIRAIV DSMKMEPNPD KTTIPLSIGD PTLCGNLPTD PEIIKALKDA LDSGKYNGYA PSIGYLSSRE [120]
Rhinolophus ferrumequinum  MDPYVIQMNG NSNLPSVLDV HVNIGGKNSK QEKGKGKKAR WSVRPSDMAN NTFNPIRTIV DNMKVKPNPH KTMIALSIGD PTVFGNLPTD PEVTQAMKDA LDSGKYNGYA PSIGYLSSRE [120]
Rhinolophus pusillus       MDPYMIQMNG NGNLPSVLDV HVNIGGKNSK QEKVKGRKAR WSVKPSDMSN KTFNPIRTIV DNMKVKPNPD KAMIALSIGD PTVFGNLPTD PEVTQAMKDA LDSGKYNGYA PSIGYLSSRE [120]
Hipposideros armiger       MDPYVIQMNS NGNFPSVLDV HVNIGGKNSK QGKVKGRKAR WSVKPSEMSN KTFNPIRTIV DGMKVKPNPN KTMISLSIGD PTVFGNLPTD PEVTQAMKDA LDSGKYNGYA PSIGYLSSRE [120]
Hipposideros pratti        MDPYVIQMNS NGNFPSVLDV HVNIGGKNSK QGKVKGRKAR WSVKPSEMSN KTFNPIRTIV DGMKVKPNPN KTMISLSIGD PTVFGNLPTD PEVTQAMKDA LDSGKYNGYA PSIGYLSSRE [120]
Megaderma lyra             MDPYVIQMN- -GTLPSVLDV HVNIGGRSSM PGKVKGRKAR WSVKPSDMSN KTFNPIRTIV DSMKVKPNPD KAMIALSIGD PTVFGNLPTD PEVTQAMKDA LDSGKYNGYA PSIGYLSSRE [120]
Megaderma spasma           MDPYVIQMN- -GTLPSVLDV HVNVDGRSSM PGKARGRKAR WSVKPSDMSN NTFNPIRTIV DSMKVKPNPD KAMIALSIGD PTVFGNLPTD PEVTQAMKDA LDSGKYNGYA PSIGYLSSRE [120]
Myotis ricketti            MDPYVIEMND SGDLPSVLDV HVNVGGRSST PGKAKGRKAR WSVKPSDMSN NTFNPIRAIV DSMKVKPNPD KTMISLSIGD PTVFGNLPTD PEVTQAMKDA LDSGKYNGYA PSIGYLSSRE [120]
Pipistrellus abramus       MDPYVIEMNG SGD------- HVSVGGRSST LGKAKGRKAR WSVKPSDMAN NTFNPIRAIV DSMKVKPNPD KTMISLSIGD PTVFGNLPTD PEVTQAMKDA LDSGKYNGYA PSIGFLSSRE [120]
Scotophilus kuhlii         MDPYMIETNG SGDLPSVLDV H--VGGRSST PGKGRGRKAR WSVKPSDMSN NTFNPIRAIV DSMKVKPNPD KAMISLSIGD PTVFGNLPTD PEVTQAMKDA LDSGKYNGYA PSIGYLSSRE [120]
Miniopterus fuliginosus    MDPYVIQMNG SGNLPSVVDV HVNIGGRSSM PGKAKGRKAR WSVKPSDMSN KTFNPIRAIV ESMKVKPNPD KTMISLSIGD PTVFGNLPTD PEVTQAMKDA LDSGKYNGYA PSIGYLSSRE [120]
Tadarida plicata           MDPYVIQMNG KSPLPSVLDV PVNIGGRSSM PGKAKGRKAR WSVKPSEMSK NTFNPIRAIV DSMKVKPNPD KTMISLSIGD PTVFGNLPTD PEVIQAMKDA LDSGKYNGYA PSIGYLSSRE [120]
Mormoops megalophylla      MDLYVIQMNG KGSLPPVLDV HVNIGGKNSL PGKGKGRKAR WSVKPSDMSN NTFNPIRAIV DSMKVKPNPD KPMISLSIGD PTVFGNLPTD PEVTQAMKDA LDSGKYNGYA PSIGYLSSRE [120]
Pteronotus parnellii       MDPYLIQMNG MGNLPSVLDV HVNVGGRNST LGKGKGRKAR WSVKPSDMSN NTFNPIRAIV DSMKVKPNPD KTMISLSIGD PTVFGNLPTD PEVTQAMKDA LDSGKYNGYA PSIGYLSSRE [120]
Desmodus rotundus          MDPYVIQMNG KGSLPSLLDM HVNVGGKNSQ PGKGKGRKAR WSVKPSDMAN NTFNPIRDIV DGMKVKPNPA KAMISLSIGD PTVFGNLPTD PEVIQAMKDA LDSGKYNGYA PSIGYLSSRE [120]
Leptonycteris yerbabuena   MDPYVIQMNR KGSLPSLLDM HVNVGGKNSL PGKGKGRKAR WSVKPSDMAN NTFNPIRAIV DNMKVKPNPD KTMISLSIGD PTVFGNLPTD PEVTQAMKDA LDSGKYNGYA PSIGYLCSRE [120]
Artibeus lituratus         MDPYVIQMNR KGSLPSLLNV HVNIGGKNSL PGKGKGRKAR WSVKPSAMAN NTFNPIRAIV DSMKVKPNPD KTMISLSIGD PTVFGNLPTD PEVTQAMKDA LDSGKYNGYA PSIGYLSSRE [120]
                                                            C151Y *    V164A      L171P                                  L201R *               P220S *               K232D
human                      EIASYYHCPE APLEAKDVIL TSGCSQAIDL CLAVLANPGQ NILVPRPGFS LYKTLAESMG IEVKLYNLLP EKSWEIDLKQ LEYLIDEKTA CLIVNNPSNP CGSVFSKRHL QKILAVAARQ [240]
mouse                      EVASYYHCPE APLEAKDVIL TSGCSQAIEL CLAVLANPGQ NILIPRPGFS LYRTLAESMG IEVKLYNLLP EKSWEIDLKQ LESLIDEKTA CLVVNNPSNP CGSVFSKRHL QKILAVAERQ [240]
rat                        EVASYYHCHE APLEAKDVIL TSGCSQAIEL CLAVLANPGQ NILIPRPGFS LYRTLAESMG IEVKLYNLLP EKSWEIDLKQ LESLIDEKTA CLVVNNPSNP CGSVFSKRHL QKILAVAERQ [240]
cow                        EVASYYHCPE APLEAKDVIL TSGCSQAIEL CLAVLANPGQ NILVPRPGFS LYRTLAESMG IEVKLYNLLP EKNWEIDLKQ LESLIDEKTV CLIVNNPSNP CGSVFSRRHL QKILAVAARQ [240]
dog                        EIASYYHRPE APLEAKDVIL TSGCSQAIEL CLAVLANPGQ NILVPRPGFS LYRTLAESMG IEVKLYNLLP EKSWEIDLKQ LESLIDEKTA CLIVNNPSNP CGSVFSKSHL QKILAVAARQ [240]
pig                        EVASYYHCPE APLEAKDVIL TSGCSQAIEL CLAVLANPGQ NILVPRPGFP LYRTLAESMG IEVKLYNLLP EKSWEIDLKH LESLIDEKTA CLVVNNPSNP CGSVFSRNHL QKILAVATRQ [240]
panda                      EIASYYHRPE APLEAKDVIL TSGCSQAIEL CLAVLANPGQ NILVPRPGFS LYRTLAESMG IEVKLYNLLP EKSWEIDLKQ LESLIDEKTA CLIVNNPSNP CGSVFSKSHL QKILAVAAKQ [240]
horse                      EIASYYHHPE APLEAKDVIL TSGCSQAIEL CLAVLANPGQ NILVPRPGFA LYRTLAESMG IEVKCYNLLP EKSWEIDLKQ VESLVDEKTA CLIVNNPSNP CGSVFSKSHL QKILAVAARQ [240]
Cynopterus sphinx          EVASYFRCPE APLEAKDVIL TSGCSQAVEL CLAVLANPGQ NILAPRPGFS PYRTLAESMG IKIKLYNLLP EKSWEIDLKQ LESLIDEKTA CLIVNNPSNP CGSVFSKSHL QDILAVAARQ [240]
Rousettus leschenaultii    EIASYYHHPK APLEAKDVIL TCGCSQAIEL CLAVLANPGQ NILAPRPGFS PYRTHAESMG IELKLYNLLP EKSWEIDLKQ MESLIDEKTA CLIVNNPSNP CGSVFSKSHL QDILAVAARK [240]
Eonycteris spelaea         EVASYYHHPK APLEAKDVIL TCGCSQAIEL CLTVLANPGQ NILAPRPGFS PYRTLAESLG VEVKLYNLLP EKYWEIDLEQ LESLIDEKTA CLIVNNPSNP CGSVFSKSHL QDILAVAARK [240]
Pteropus vampyrus          EIASYYHRPE APLEAKDVIL TSGCSQAIEL CLAVLANPGQ NILAPRPGFS PYRTLAESLG IEIKLYNLLP EKSWEVDLKQ LESLIDEKTA CLIVNNPSNP CGSVFSKSHL QDILAVAARQ [240]
Rhinolophus ferrumequinum  EIASYYHCPE APLEANDVIL TSGCGQAIEL CLAVLANPGQ NILLPRPGFS LYRTLAESMG IEVKLYNLLP EKSWEIDLKQ LKSLIDEKTA CLIVNNPSNP CGSVFSKNHL QKILAVAARQ [240]
Rhinolophus pusillus       EIASYYHCPE APLEAKDVIL TSGCSQAIEL CLAVLANPGQ NILIPRPGFS LYRTLAESMG IEVKLYNLLP EKSWEIDLKQ LESLIDEKTA CLIVNNPSNP CGSVFSTNHL QKILAVAARQ [240]
Hipposideros armiger       EVASYYHCPE APLEAKDVIL TSGCSQAIEL CLAVLANPGQ NILVPRPGFS LYRTLAESMG IEVKLYNLLP EKSWKIDLKQ LEALIDEKTA CLIVNNPSNP CGSVFSKSHL QKILAVAARQ [240]
Hipposideros pratti        EVASYYHCPE APLEAKDVIL TSGCSQAIEL CLAVLANPGQ NILVPRPGFS LYRTLAESMG IEVKLYNLLP EKSWKIDLKQ LEALIDEKTA CLIVNNPSNP CGSVFSKSHL QKILAVAARQ [240]
Megaderma lyra             EIASYYHCPE APLEAKDIIL TSGCSQAIEL CLAVLANPGQ NILVPRPGFS LYKTLAESMG IEVKLYNLLP EKSWEIDLKQ LESLIDEKTA CLIVNNPSNP CGSVFSKSHL QKILAVAARQ [240]
Megaderma spasma           EIASYYHCPE APLEAKDVIL TSGCSQAIEL CLAVLANPGQ NILVPRPGFS LYKTLAESMG IEVKLYNLLP EKSWEIDLKQ LESLIDEKTA CLIVNNPSNP CGSVFSKSHL QKILAVAARQ [240]
Myotis ricketti            EVASYYHCPE APLEAKDVIL TSGCSQAIEL CLAVLANPGQ NILVPRPGFS LYRTLAESMG IEIKLYNLLP EKSWEIDLKQ LESLIDEKTA CLIVNNPSNP CGSVFSRSHL QKILAVAARQ [240]
Pipistrellus abramus       EVASYYHCPE APLEAKDVIL TSGCSQAIEL CLAVLANPGQ NILVPRPGFS LYRTLAESMG IEVKLYNLLP EKSWEIDLKQ LESLIDEKTA CLIVNNPSNP CGSVFSRSHL QKILAVAARQ [240]
Scotophilus kuhlii         EVASYYHCPE APLEAEDVIL TSGCSQAIEL CLAVLANPGQ NILVPRPGFS LYRTLAESMG IEVKLYNLLP EKSWEIDLKQ LESLIDEKTA CLIVNNPSNP CGSVFSRSHL QKILAVAARQ [240]
Miniopterus fuliginosus    EIASYYHCPE APLEAKDVIL TSGCSQAIEL CLAVLANPGQ NILVPRPGFS LYRTLAESMG IEIKLYNLLP EKSWEADLKQ LESLIDEKTA CLIVNNPSNP CGSVFSRSHL QKILAVAARQ [240]
Tadarida plicata           EIASYYHCPE APLEAKDVIL TSGCSQAIEL CLAVLANPGQ NILVPRPGFS LYRTLAESMG IEIKLYNLLP EKSWEIDLKQ LESLIDEKTA CLIVNNPSNP CGSVFSRSHL QKILAVAARQ [240]
Mormoops megalophylla      EIASYYHCPE APLEAKDVIL TSGCSQAIEL CLAVLANPGQ NILVPRPGFS LYRTLAESLG VEVRFYNLLP EKSWEIDLKQ LESLIDEKTA CLIINNPSNP CGSVFRRSHL QKILAVAARQ [240]
Pteronotus parnellii       EIASFYHCPE APLEAKDVIL TSGCSQAIEL SLAVLANPGQ NILVPRPGFS LYRTLAESMG IEVKLYNLLP EKSWEVDLKQ LESLIDEKTA CLIVNNPSNP CGSVFSRSHL QEILAVAAKQ [240]
Desmodus rotundus          EIVSYHHCPE APLEAKDVIL TSGCSQAIEL CLAVLANPGQ NILVPRPGFS LYRTLTESMG IEIKLYNLLP EKSWEIDLKQ LESLIDEKTA CLVVNNPSNP CGSVFSRSHL QKILAVAARQ [240]
Leptonycteris yerbabuena   DIASYYHCPE APLEAKDVLL TSGCSQAIEL CLAVLANPGQ NILVPRPGFS LYRTLAESMG IEVKLYNLLP EKSWEIDLKQ LESLIDEKTA CLIVNNPSNP CGSVFSRSHL QKILAVAARQ [240]
Artibeus lituratus         EIASYYHCPE APLEAKDVIL TSGCSQAIEL CLAVLASPGQ NILVPRPGFS LYRTLAESMG IEVKLYNLLP EKSWEIDLKQ LESLIDEKTA CLIVNNPSNP CGSVFSRSHL QKILTVAARQ [240]
                                                  S258T  L263I S268R L273P *                           D307N      L317M                                  G355E
human                      CVPILADEIY GDMVFSDCKY EPLATLSTDV PILSCGGLAK RWLVPGWRLG WILIHDRRDI FGNEIRDGLV KLSQRILGPC TIVQGALKSI LCRTPGEFYH NTLSFLKSNA DLCYGALAAI [360]
mouse                      CVPILADEIY GDMVFSDCKY EPMATLSTNV PILSCGGLAK RWLVPGWRLG WILIHDRRDI FGNEIRDGLV KLSQRILGPC TIVQGALKSI LQRTPQEFYQ DTLSFLKSNA DLCYGALSAI [360]
rat                        CVPILADEIY GDMVFSDCKY EPLANLSTNV PILSCGGLAK RWLVPGWRLG WILIHDRRDI FGNEIRDGLV KLSQRILGPC TIVQGALKSI LQRTPQEFYH DTLSFLKSNA DLCYGALAAI [360]
cow                        CVPILADEIY GDMVFSDSKF EPLATLSSKV PILSCGGLAK RWLVPGWRMG WILIHDRRDI FGNEIRDGLT KLSQRILGPC TLVQGALKSI LCRTPRVFYH NTLSFLKSNA DLCYGALAAI [360]
dog                        CVPILADEIY GDMVFSDSKF EPLATLSSNV PILSCGGLAK RWLVPGWRLG WILIHDRRDI FGNEIRDGLV KLSQRILGPC TIVQGALKSI LRRTPQEFYQ NTLSFLKSNA DLCYGALAAI [360]
pig                        CVPILADEIY GDMVFSDSKF EPLATLSSNV PILSCGGLAK RWLVPGWRLG WILIHDRRDI FGNEIRDGLV KLSQRILGPC TLVQGALKSI LRRTPQEFYH NTLSFLKTNA DLCYGALAAV [360]
panda                      CVPILADEIY GDMVFSDSKF EPLATLSSNV PILSCGGLAK RWLVPGWRLG WILIHDRRDI FGNEIRDGLV KLSQRILGPC TIVQGALKSI LHRTPQEFYH NTLSFLKSNA DLCYGALAAI [360]
horse                      CVPIIADEIY GDMVFSDSKF EPLATLSSNV PILSCGGLAK RWLVPGWRLG WILIHDRRDI FGNEIRDGLV KLSQRILGPC TIVQGALKNI LRRTPQEFYH NTLSLLKSNA DLCYGALAAI [360]
Cynopterus sphinx          CVPIVADEIY GDMVFSDTKF EPIATLSRSV PILCCGGLTK RWLVPGWRLG WILIHDRGDI FGNEIRNGLV KLSQRIMGPC TLVQGALKSI LRRTPQEFYH NTLSFLKSSA DLCYVALSAI [360]
Rousettus leschenaultii    YVPILADEIY GDMVFSDTKF EPIATLSRNV PILTCGGLAK RWLVPGWRLG WILIHDRRDI FGNEIRNGLV KLCQRIMGPC TIVQGALKSI LRRTPQEFYH NTLSFLKSNA DLCYEALAAI [360]
Eonycteris spelaea         YVPILADEIY GDMVFSDTKF VPIATLSRNV PILTCGGLAK RWLVPGWRLG WIFIHDRKDI FGNEIRNGLV RLCQRIMGPC TIVQGALKSI LRRTPQEFYH NTLSFLKSSA DLCYEALAAI [360]
Pteropus vampyrus          CVPILADEIY GDMMFSDTKF EPIATLSHNV PILSCGGLAK RWLVPGWRLG WILIHDRGDI FGNEIPNGLV KLSQRIMGPC TVVQGALKSI LRRTPQEFYR NTLSVLKSSA DLCYEALSAI [360]
Rhinolophus ferrumequinum  CVPILADEIY GDMVFSDSKF EPLATLSSNV PILSCGGLAK RWLVPGWRLG WILIHDRKDI FGNEIRDGLV KLSQRILGPC TIVQGALKSI LHRTPQEFYH NTLSFLKSNA DLCYGALAAI [360]
Rhinolophus pusillus       CVPILADEIY GDMVFSDSKF EPLATLSSNV PILSCGGLAK RWLVPGWRLG WILIHDRKDI FGNEIRDGLV KLSQRILGPC TIVQGALKSI LHRTPQEFYH NTLSFLKSNA DLCYGALAAI [360]
Hipposideros armiger       CVPILADEIY GDMVFSDSKF EPLANLSTNV PILSCGGLAK RWLVPGWRLG WILIHDRRNI FGNEIRDGLV KLSQRILGPC TLVQGALKNI LRRTPQEFYH NTLSFLKSNA DLCYGALSAI [360]
Hipposideros pratti        CVPILADEIY GDMVFSDSKF EPLANLSTNV PILSCGGLAK RWLVPGWRLG WILIHDRRNI FGNEIRDGLV KLSQRILGPC TLVQGALKNI LRRTPQEFYH NTLSFLKSNA DLCYGALSAI [360]
Megaderma lyra             CVPILADEIY GDMVFSDSKF EPLATLSSNV PILSCGGLAK RWLVPGWRLG WILIHDRRDI FGNEIRDGLV KLSQRILGPC TIVQGALKSI LRRTPQEFYH NTLSFLKSNA DLCYGALAAI [360]
Megaderma spasma           CVPILADEIY GDMVFSDSKF EPLATLSGHV PILSCGGLAK RWLVPGWRLG WILIHDRRDI FGNEIRDGLV KLSQRILGPC TIVQGALKSI LRRTPQEFYH NTLSFLKSNA DLCYGALAAI [360]
Myotis ricketti            CVPILADEIY GDMVFSGSKF EPLATLSSNV PILSCGGLAK RWLVPGWRLG WILIHDRRDI FGNEIRDGLV KLSQRILGPC TIVQGALKSI LRRTPQEFYH DTLSFLKSNA DLCYGALAAI [360]
Pipistrellus abramus       CVPILADEIY GDMVFSGSKF EPLATLSTNV PILSCGGLAK RWLVPGWRLG WILIHDRRDI FGNEIRDGLV KLSQRILGPC TIVQGALKSI LRRTPQEFYH DTLSFLRSNA DLCYGALAAI [360]
Scotophilus kuhlii         CVPILADEIY GDMVFSGSKF EPLATLSSNV PILSCGGLAK RWLVPGWRLG WILIHDRRDI FGNEIRDGLV KLSQRILGPC TIVQGALKSI LRRTPQEFYH DTLSFLKSNA DLCYGALAAI [360]
Miniopterus fuliginosus    CVPILADEIY GDMVFSDSKF EPLATLSSNV PILSCGGLAK RWLVPGWRLG WILIHDRKGI FGNEIRDGLV KLSQRILGPC TIVQGALKSI LHRTTQEFFQ DTLSFLKSNA DLCYGALAAI [360]
Tadarida plicata           CVPILADEIY GDMVFSDSKF EPLATLSSNV PILSCGGLAK RWLVPGWRLG WILIHDRRDI FGNEIRDGLV KLSQRILGPC TIVQGALKSI LRRTPQEFYH NTLSFLKSNA DLCYGALAAI [360]
Mormoops megalophylla      CVPILADEIY ADMVFSDSKF EPLANLSSDV PILSCGGLAK RWLVPGWRLG WILIHDRRDI FGNEIRDGLV KLSQRILGPC TIVQGALKSI LRRTPREFYH NTLSFLKSSA DLCYGALAAI [360]
Pteronotus parnellii       CVPILADEIY GDMVFSDSKF EPLANLSSNV PILSCGGLAK RWLVPGWRLG WILIHDRRDI FGNEIRDGLV KLSQRILGPC TIVQGALKSI LRRTPQEFYH NTLSFLKSNA DLCYGALAAV [360]
Desmodus rotundus          CVPILADEIY GDMVFSDSKF EPLASLSSNV PILSCGGLAK RWLVPGWRMG WILIHDRRDI FGNEIRDGLV ELSQRILGPC TIVQGALKSI LRRTPQEFYQ NTLSFLKSNG DLCYGALAAI [360]
Leptonycteris yerbabuena   CVPILADEIY GDMVFSNSKF EPLANLSSNV PILSCGGLAK RWLVPGWRLG WILVHDRRDI FGNEIRDGLV KLSQRILGPC TIVQGALKSI LRRTPQEFYH NTLSFLKSNA DLCYGALAAI [360]
Artibeus lituratus         CVPILADEIY GDMVFSDSKF EPLANLSSNV PILSCGGLAK RWLVPGWRLG WILIHDRRDI FGNEIRDGLV KLSQRILGPC TIVQGALKSI LRRTPQEFYH NTLSFLKSNA DLCYGALAAI [360]
                           G362V *                          R395Q V397F A398V                                  R433Q/W *
human                      PGLRPVRPSG AMYLMVGIEM EHFPEFENDV EFTERLVAEQ SVHCLPATCF EYPNFIRVVI TVPEVMMLEA CSRIQEFCEQ HYHCAEGSQE ECDK [454]
mouse                      PGLQPVRPSG AMYLMVGIEM EHFPEFENDV EFTERLIAEQ SVHCLPATCF EYPNFFRVVI TVPEVMMLEA CSRIQEFCEQ HYHCAEGSQE ECDK [454]
rat                        PGLQPVRPSG AMYLMVGIEM EHFPEFENDV EFTERLIAEQ AVHCLPATCF EYPNFFRVVI TVPEVMMLEA CSRIQEFCEQ HYHCAEGSQE ECDK [454]
cow                        PGLRPIHPSG AMYLMVGIEM EHFPEFENDV EFTEQLVAEQ SVHCLPATCF EYPNFFRVVI TVPEVMMLEA CSRIQEFCEQ HYHCAEGSQE ECDK [454]
dog                        PGLRPVRPSG AMYLMVGIEM EHFPEFENDV EFTERLVAEQ SVHCLPATCF EYPNFFRVVI TVPKVMMLEA CSRIQEFCEL HYHCAEGSQE ECDK [454]
pig                        PGLRPIRPCG AMYLMVGIEM EQFPEFENDV EFTERLVAEQ SVHCLPGMCF EYRNFFRVVI TVPKVMMLEA CSRIQEFCEQ HYQCAEGTQE ECDK [454]
panda                      PGLQPVRPSG AMYLMVGIEM EHFPEFENDV EFTERLVAEQ SVHCLPATCF EYPNFFRVVI TVPKVMMLEA CSRIQEFCEL HYHCAEGSQE ECDK [454]
horse                      PGLRPVRPSG AMYLMVGIEM EHFPEFENDV EFTERLVAEQ SVHCLPGTCF EYLNFFRVVI TVPKVMMLEA CSRIQEFCEQ HYHCAEGSQE ECDK [454]
Cynopterus sphinx          PGLRPVRPSG AMYLMVEIEM EHFPEFENDV EFTERLFVEQ SVHCLPAMCF EYPNFFRVVI AVPKVTILEA CSRIQEFCEQ HYHHAEGIQE ECDK [454]
Rousettus leschenaultii    PGLRPVRSSG AMYIMVEIQM EHFPEFENDV EFTEQLFVEQ SVQCLPAKCF EYPNFFRVVI TVPKVMMLEA CSRIQEFCEQ HYHLAEGSQE ECDK [454]
Eonycteris spelaea         PGLRPVHSSG AMYLMVEIEM EHFPEFENDV EFTEQLFVEQ SVHCLPAKCF EYPNFFRVVI TVPKVMMLEA CSRIQEFCEQ HYHHAEGTQE ECDK [454]
Pteropus vampyrus          PGLRPVRPSG AMYLMVEIEM EHFPEFENDV EFTEQLFVEQ SVLCLPAICF EYPNFFRVVI AVPKVMILEA CSRIQEFCEQ HYHHAEGIQE ECDK [454]
Rhinolophus ferrumequinum  PGLRPVRPSG AMYLMVEIEM EHFPEFENDV EFTERLVAEQ SVHCLPATCF EYPNFFRVVI TVPEVMMLEA CSRIQEFCEK HYHCAEGSQE ECDK [454]
Rhinolophus pusillus       PGLRPVRPSG AMYLMVEIEM EHFPEFKNDV EFTERLVAEQ SVHCLPATCF EYPNFFRVVI TVPEVMMLEA CSRIQEFCEK HYHCAEGSQE ECDK [454]
Hipposideros armiger       PGLRPVRPSG AMYLMVEIEM EHFPEFENDV EFTERLVAEQ SVHCLPATCF EYPNFFRVVI TVPEVMMLEA CGRIQEFCEQ HYHCAEGSQE ECDK [454]
Hipposideros pratti        PGLRPVRPSG AMYLMVEIEM EHFPEFENDV EFTERLVAEQ SVHCLPATCF EYPNFFRVVI TVPEVMMLEA CGRIQEFCEQ HYHCAEGSQE ECDK [454]
Megaderma lyra             PGLRPVRPSG AMYLMVEIEM EHFPEFKDDV EFTERLVAEQ SVHCLPATCF EYPNFFRVVI TVPEVMMLEA CSRIQEFCER HYHGAEGSQE ECDK [454]
Megaderma spasma           PGLRPVRPSG AMYLMVEIEM EHFPEFKDDV EFTERLVAEQ SVHCLPATCF EYPNFFRVVI TVPEVMMLEA CSRIQEFCER HYHGAEGSQE ECDK [454]
Myotis ricketti            PGLRPVRPSG AMYLMVEIEM EHFPEFENDV EFTERLVAEQ SVHCLPATCF EYPNFFRVVI TVPEVMILEA CSRIQEFCEQ HYHCAEGSPE ECDK [454]
Pipistrellus abramus       PGLRPVRPSG AMYLMVEIEM EHFPEFENDV EFTERLIAEQ SVHCLPATCF EYPNFFRVVI TVPEVMMLEA CSRIQEFCEQ HYHHAEGSPE ECDK [454]
Scotophilus kuhlii         PGLRPVRPSG AMYLMVEIEM EHFPEFENDV EFTERLVAEQ SVHCLPATCF EYPNFFRVVI TVPEVMMLEA CSRIQEFCEQ HYHCAEGSPE ECDK [454]
Miniopterus fuliginosus    PGLRPVRPSG AMYLMVEIEM EHFPEFENDV EFTERLVAEQ SVHCLPATCF EYPNFFRVVI TVPEVMMLEA CSRIQEFCEQ HYHSAEGSHE ECDK [454]
Tadarida plicata           PGLRPVRPSG AMYLMVEIEM EHFPEFENDV DFTERLVAEQ SVHCLPATCF EYPNFFRVVI TVPEVMMLEA CSRIQEFCEQ HYHCAEGSQE ECDK [454]
Mormoops megalophylla      PGLRPIRPLG AMYLMVEIEM EHFPEFENDE EFTERLIAEQ SVQLLPAKCF EYPNFFRVVI TVPEVMMLEA CSRIQEFCEQ HYHCAEGSQE ECDK [454]
Pteronotus parnellii       PGLRPVRPSG AMYLMVEIQM EHFPEFENDV EFTERLVAEQ SVHCLPATCF EYPNFFRVVI TVPEVMMLEA CSRIQEFCEQ HYHCAEGSQE ECDK [454]
Desmodus rotundus          PGLRPVRPSG AMYLMVEIEM EHFPEFGNDV EFTERLVAEQ SVHCLPATCF GYPNFFRVVI TVPEVMILEA CSRIQKFCEQ HYHSAEGGQE ECDK [454]
Leptonycteris yerbabuena   PGLRPVRPSG TMYLMVEIEM EHFPEFENDV EFTERLVAEQ SVHCLPATCF EYPNFFRVVI TVPKVMMLEA CSRIQEFCEQ HYHCAEGSQE ECDK [454]
Artibeus lituratus         PGLWPVRPSG AMYLMVKIEM EHFPEFENDV EFTERLVAEQ SVHCLPATCF EYPNFFRVVI TAPEVMMLEA CSRIQEFCEQ HYHCAAGSQE ECDK [454]
```
